# The effect of child access prevention laws on adolescent suicide: a negative control approach

**DOI:** 10.1186/s40621-025-00577-x

**Published:** 2025-04-23

**Authors:** Sean MacAllister, Matthew Miller, Sonja Swanson

**Affiliations:** 1 Department of Epidemiology, University of Pittsburgh School of Public Health, 130 De Soto St, Pittsburgh, PA 15261 USA; 2 Department of Public Health and Health Sciences, Northeastern University Bouvé College of Health Sciences, 360 Huntington Avenue, Boston, MA 02115 USA

**Keywords:** Bias analysis, Child access prevention laws, Adolescent suicide, Firearm suicide, Youth firearm suicide, Negative controls

## Abstract

**Background:**

Recent publications on Child Access Prevention (CAP) laws suggest substantial protective effects on adolescent firearm suicide. However, these studies have also found comparable protective effect estimates on adolescent non-firearm suicide and adult firearm suicide, which may indicate residual confounding. Here we apply bias analysis techniques to assess the effects of CAP laws while accounting for potential unmeasured sources of bias using a negative control approach.

**Method:**

Using established bias formulas, we bias-adjust previously published point estimates and their 95% confidence intervals (CI) assuming that an arbitrary confounder biases all suicide-related effect estimates and that adolescent non-firearm suicide and adult firearm suicide are negative controls. Negative controls are outcomes or populations that prior subject matter suggests should not be meaningfully affected by the exposure and can be used to better understand and sometimes account for bias in the primary exposure-outcome relationship.

**Results:**

After bias adjustments, effect estimates were attenuated, with many of the confidence intervals including the null. Assuming that adolescent non-firearm suicide is a negative control outcome and taking a published point estimate as the bias parameter, the bias-adjusted effect estimate for adolescent firearm suicide decreased from an incidence rate ratio of 0.87 (95% CI: 0.78, 0.97) to 0.95 (95% CI: 0.85, 1.07). When adult firearm suicide was used as the negative control, the bias-adjusted estimate was 0.92 (95% CI: 0.82, 1.03).

**Conclusion:**

Our findings suggest that CAP laws may have had a smaller public health impact on adolescent suicide than previously estimated. Given the strong evidence that reducing *access* to firearms can prevent suicide deaths, and that secure storage helps reduce access for many children, our findings underscore the need to continue to identify and promote effective ways to motivate adults to make household firearms inaccessible to children.

**Supplementary Information:**

The online version contains supplementary material available at 10.1186/s40621-025-00577-x.

## Introduction

Child Access Prevention (CAP) laws hold firearm owners legally responsible for harm or potential harm caused by minors who gain access to firearms that were not stored securely. As of January 1, 2025, 35 states and the District of Columbia have implemented some version of CAP laws [1]. The strictest laws impose criminal penalties for negligent storage regardless of whether a child gains access to a gun owner’s firearms.

CAP laws are motivated by the hope that legislation can meaningfully induce gun owners to make their firearms inaccessible to children, and by research suggesting that locking up all the firearms in a child’s home will substantially mitigate the heightened risk of firearm injury and death that children face when they live in a home with guns [2]. The extent to which CAP laws reduce the risk of firearm injury to youth, however, remains an unsettled question. For example, a comprehensive report by RAND, published in July of 2024, concluded that the totality of evidence from ecologic studies was “supportive” of a protective effect of these laws on youth firearm suicide (and youth firearm homicide), though the studies contributing to their review vary considerably on what the size of that effect may be (estimated incidence rate ratios (IRR) of 0.24 to 0.90) [3]. In addition, survey-based research has raised questions about whether most gun owners know if they live in a state with a CAP law and, more generally, about whether CAP laws meaningfully affect firearm storage practices in households with children [4].

Neither RAND’s review of CAP law studies, nor the studies included in the review themselves, explicitly applied “negative control” approaches– defined below– to qualitatively or quantitatively inform their conclusions [3]. Fortunately, one of the recent studies cited in the RAND review, by Kivisto and colleagues [5], published sufficient information to derive bias-adjusted point estimates under the assumption that *non*-firearm suicide is a negative control outcome (i.e., the effect of CAP laws on non-firearm suicide should be null). In that study, Kivisto and colleagues estimated that CAP laws produce a 13% reduction in the adolescent firearm suicide rate but also a 9% reduction in the adolescent non-firearm suicide rate (IRR for firearm suicide: 0.87 [95% confidence interval (CI): 0.78, 0.97]; IRR for non-firearm suicide: 0.91 [95% CI: 0.84, 0.99]) [5]. In the same study, Kivisto and colleagues [5] also published information about the effect of CAP laws on *adult* firearm suicide, IRR: 0.94 (95% CI: 0.92, 0.97), allowing a separate bias-adjusted estimate to be derived under the assumption that *adult* firearm suicide is a negative control population (i.e., that the effect of CAP laws on adult firearm suicides should be approximately null).

The current study extends prior work by proposing negative controls to guide inference and leveraging these negative controls to bias-adjust existing estimates from the CAP literature [6]. Specifically, we produce two sets of bias-adjusted estimates of the effect of CAP laws on youth suicide. The first set assumes that non-firearm suicide is a negative control outcome. The second set assumes that the adult population is a negative control population.

The bias-adjusted analyses we pursue in this paper are motivated by the concern that published estimates of the effect of CAP laws on suicide likely overestimate the causal effect of CAP laws on adolescent firearm suicide. This concern stems from the observation that published estimates to date have not attempted to account for the relatively large “protective” effect of CAP laws on non-firearm suicide and on adult firearm suicide that have been reported in the few studies that have publish such estimates alongside estimates of the effect of CAP laws on adolescent firearm suicide [6]. In the methods section below we formalize this intuition and elaborate our rationale for choosing the negative controls we use. Informally, this intuition rests on two conjectures: (1) that CAP laws exert their effect primarily on firearm suicide, and (2) that firearm owners, who as a group constitute the great majority of firearm suicide decedents, can generally access their own firearms even when their guns are secured against a child gaining access [7, 8 ].

## Methods

### Proposing negative controls in firearm policy evaluation

Negative control approaches interrogate assumptions underlying a causal inquiry (here, CAP laws’ effect on adolescent firearm suicide) by conducting or conceptualizing an analysis that mirrors the primary analysis but substitutes a population (e.g., adults), an exposure (e.g., an unrelated law), or an outcome (e.g., non-firearm suicide) that prior subject matter suggests should result in a null (or near null) effect [9–11]. If a substantively meaningful non-null result is found, it can suggest residual confounding in the primary analysis. If uncontrolled confounding is identified through a negative control, corrections to the primary effect estimate can be made using previously developed bias formulas [12–14] examples of which can be found in the firearm-suicide literature [15, 16]. Such bias formulas can either postulate a specific source of uncontrolled confounding or formulate bias due to failing to control for an arbitrary unknown confounder. As previewed in the introduction, non-firearm suicides constitute a reasonable negative control outcome in this setting as CAP laws target access to firearms, not other means commonly used in suicidal acts. This expectation follows from dozens of individual-level studies that consistently show a largely method-specific effect of firearm access on firearm suicide [7, 17–23].

Additionally, one may propose firearm suicide in adults as a negative control outcome or population in this setting. CAP laws target children and, as such, any spillover effect they may have on adults is likely to be much smaller in magnitude than the effect they have on youth. Indeed, any collateral benefit to adults is likely to be conferred primarily on adults who live in a home with firearms but do not personally own any of the household’s guns. This group comprises approximately 10% of all US adults (mostly women)– and only approximately 3% of adults in households with children and firearms [23, 24].

## Bias analysis methods

We begin with the effect estimates and 95% CIs reported by Kivisto and colleagues [5] specific to CAP laws mandating safe storage regulations and standards, and present bias-adjusted estimates under two sets of assumptions about our proposed negative controls. We used the study by Kivisto and colleagues [5] because all the point estimates needed for the current study are reported in the published manuscript. The premise of each assumption set is outlined below (formulas and derivations are provided in the supplementary material under Supplement 1). For both approaches, we expand upon previously derived bias formulas developed under the assumptions that (i) an unnamed binary confounder biases all suicide-related effect estimates (i.e., adolescent firearm suicide, adolescent non-firearm suicide, and adult firearm suicide), and (ii) that this confounder’s association with each of these suicide outcomes is constant, on the relative scale, within levels of exposure to CAP laws [12–14]. Of note, this hypothetical unmeasured confounder is confounding above and beyond what is already adjusted for in the original study, namely race and ethnicity, high school completion, poverty, unemployment, alcohol consumption, and region [5]. All bias analyses were conducted in R version 4.3.1 and all graphs were created using the R package ggplot2 version 3.5.1. Full code is provided in the supplementary material.

For our first set of bias analyses, we assume that adolescent non-firearm suicide is a negative control outcome such that the effect estimate of CAP laws on adolescent non-firearm suicide should be null. We further assume the non-null effect estimate reported by Kivisto and colleagues [5] (i.e., IRR: 0.913 [95% CI: 0.843, 0.988]) results from failing to adjust for a hypothetical confounder that also confounds the primary result. We bias-adjust the primary estimate and its corresponding 95% CI using the bias adjustment formula available in the supplementary material (Supplement 1). As our bias adjustment depends on the specific bias parameter input from the adolescent non-firearm suicide rate ratio, we demonstrate how uncertainty due to sampling error affects the bias parameter by repeating our bias adjustment for every value, to the third decimal, contained within the reported confidence limits (i.e., 0.843, 0.844, 0.845…., 0.988).

For our second set of bias analyses, we assume that *adult* firearm suicide is a negative control population such that the effect of CAP laws on adult firearm suicides should be null. We further assume that the non-null estimate reported by Kivisto and colleagues [5] (i.e., IRR: 0.944 [95% CI: 0.923, 0.968]) results from failing to adjust for a hypothetical confounder that also confounds the primary result. As with the first set of bias analyses, we bias-adjust the primary estimate and its corresponding CI with the bias parameter iterating over the range of the adult firearm suicide confidence limits (i.e., 0.923, 0.924, 0.925,…, 0.968).

Finally, as Kivisto and colleagues [5] further investigated specific variants of CAP laws, we repeat the negative control approaches described above for each CAP law variants for which Kivisto and colleagues provided point estimates and 95% CIs. For example, for the CAP law variant “safety lock is required for handguns sold through licensed dealers”, we bias-adjust the adolescent firearm suicide effect estimate using the corresponding adolescent non-firearm suicide effect estimate under the assumption that it should be null and also using the corresponding adult firearm suicide effect estimate under the assumption that it should be null.

## Results

**Fig. 1:**
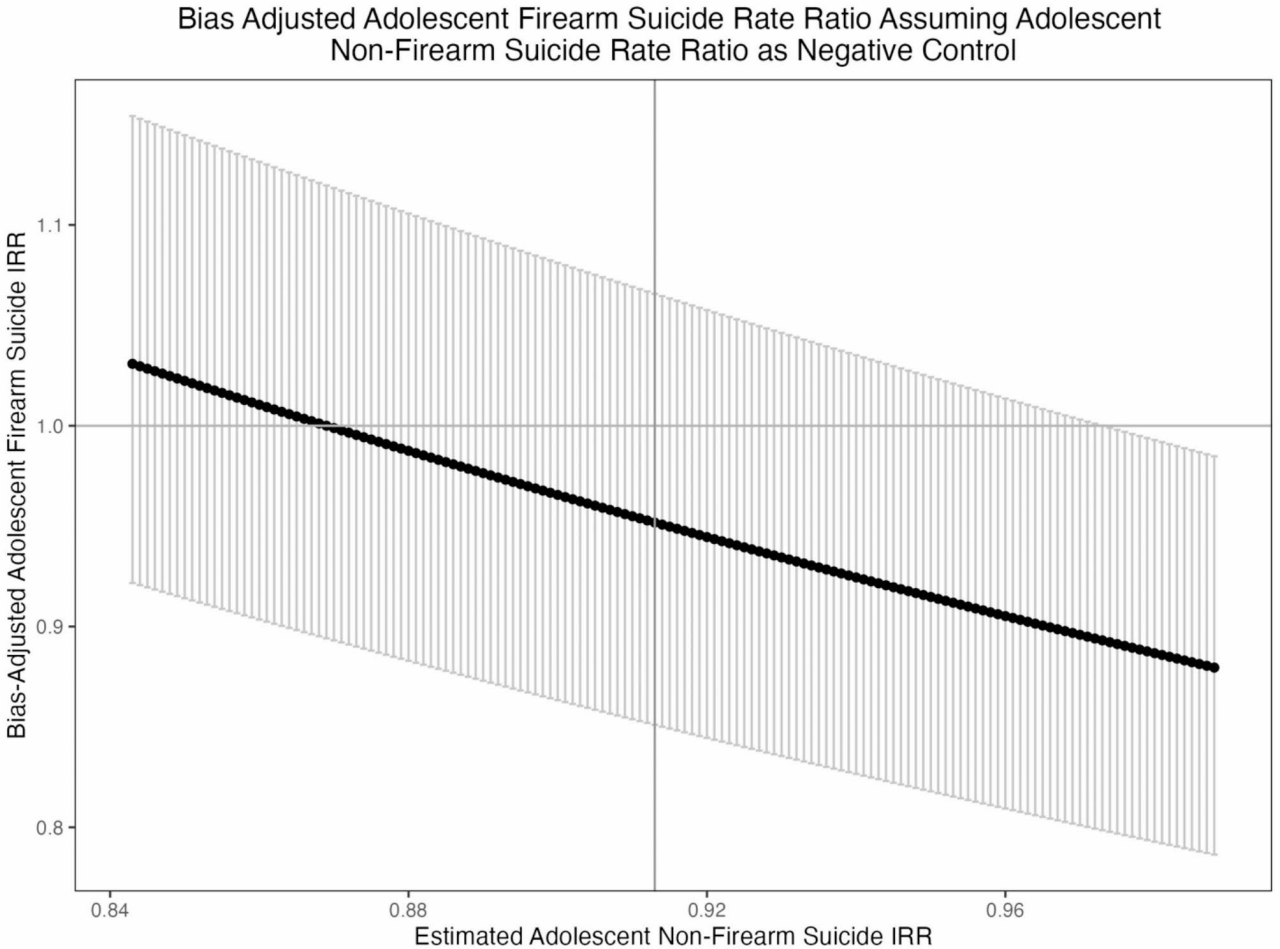
Bias Adjusted Adolescent Firearm Suicide Rate Ratio Assuming Adolescent Non-Firearm Suicide Rate Ratios as the Negative Control: Black dots represent the bias-adjusted point estimates. Error bars represent the bias-adjusted 95% confidence intervals. The vertical line originating at 0.913 on the x-axis represents the Kivisto and colleagues estimated adolescent non-firearm suicide IRR. The horizontal line on the y-axis represents the null value. IRR = Incident Rate Ratio

The first set of bias adjustments assume that adolescent non-firearm suicides is a negative control outcome (Fig. 1). Taking the point estimate for non-firearm suicides (IRR: 0.913) estimated by Kivisto and colleagues [5] as the bias parameter, our bias-adjusted effect estimate for adolescent firearm suicides is 0.952 (95% CI: 0.851, 1.066). Generally, across the whole range of bias parameters considered, the bias-adjusted effect estimate was attenuated with a majority of the bias-adjusted adolescent firearm suicide 95% CIs containing the null value. As such, CAP laws appear to have a protective effect that ranges from 0 to 15% with 5% being the most likely (taking the previously published point estimate as the bias parameter). This pattern was also predominantly evident when examining specific versions of CAP laws (Supplement 2-Figures S1 − 12).

**Fig. 2:**
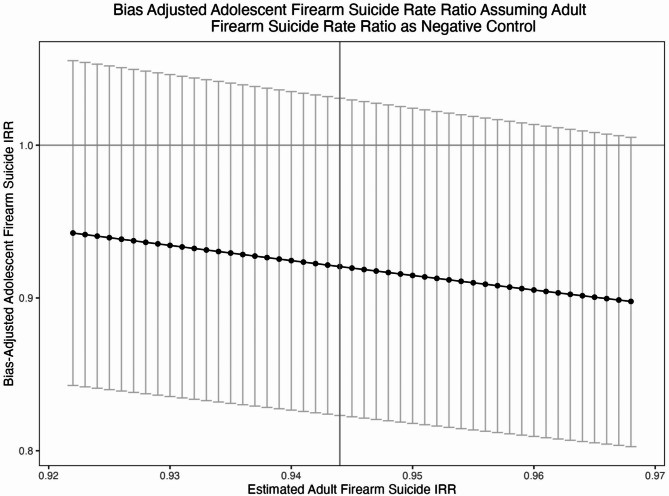
Bias Adjusted Adolescent Firearm Suicide Rate Ratio Assuming Adult Firearm Suicide Rate Ratios as the Negative Control: Black dots represent the bias-adjusted point estimates. Error bars represent the bias-adjusted 95% confidence intervals. The vertical line originating at 0.944 on the x-axis represents the Kivisto and colleagues estimated adolescent non-firearm suicide IRR. The horizontal line on the y-axis represents the null value. IRR = Incident Rate Ratio

The second set of bias adjustments assume that adult firearm suicide is a negative control population (Fig. 2). Taking the point estimate for adult firearm suicides (IRR: 0.944) estimated by Kivisto and colleagues [5] as the bias parameter, our bias-adjusted effect estimate for adolescent firearm suicide is 0.921 (95% CI: 0.823, 1.031). Across the whole range of bias parameters considered, the bias-adjusted effect estimate was attenuated with all of the bias-adjusted adolescent firearm suicide 95% CIs containing the null value. As such, CAP laws appear to have a protective effect that ranges from 0 to 18% with 8% being the most likely (taking the previously published point estimate as the bias parameter). This pattern was also evident when examining specific versions of CAP laws (Supplement 3-Figures S13 − 24).

## Discussion

In this study, we provide updated effect estimates for the CAP law-adolescent firearm suicide relationship reported by Kivisto et al. [5] under the assumptions that adolescent non-firearm suicide and adult firearm suicide serve as reasonable negative controls. Our analyses find generally attenuated effect estimates, often with 95% CIs that contained the null. Because Kivisto and colleagues [5] was the only manuscript cited in the recent RAND review that had internally consistent point estimates and also published all the effect estimates needed for the approach used in this study, we did not replicate our bias-adjusted analyses using other previously published effect estimates. However, given the broad range of bias parameters across the range of confidence intervals in Kivisto et al.‘s [5] work, our findings provide bias-adjusted results that may be reasonable proxies of how other studies’ effect estimates may change if bias-adjusted under similar assumptions.

For example, Schell and colleagues [25] estimated that across all age groups, 1,075 firearm suicides and 805 non-firearm suicides could have been avoided over a 6-year period had all states implemented CAP laws, compared to what would have occurred had no states implemented CAP laws. Although Schell et al. [25] did not publish sufficient data to allow the methodology used in this study to bias-adjust their point estimates, our bias-adjusted findings based on Kivisto and colleagues [5] suggest the potential magnitude of unaddressed bias may be reasonably similar in these studies, though our approach further points to additional analyses that investigators could perform on the original data to bias-correct published effect estimates.

We present results across a range of assumption sets and with different perspectives on sampling variability, allowing readers to consider which assumption sets they find most plausible and the estimates that align with those assumptions. On the one extreme, if a reader does not think either proposed negative control is appropriate, and also believes the prior study appropriately controlled for all confounding and other sources of bias, then they would expect CAP laws to lead to a 13% reduction in adolescent suicide rates (95% CI: 0.78, 0.97) [5]. On the other hand, if a reader believes adolescent non-firearm suicide or adult firearm suicide to be a reasonable negative control and takes the point estimates as the most likely value from the reported CIs to inform the bias analysis, the effect sizes and confidence intervals are consistent with a 8% and 5% reduction in the protection conferred by CAP laws, respectively (IRR using adolescent non-firearm suicide as the negative control: 0.952 [95% CI: 0.85, 1.066]; IRR using adult firearm suicide as the negative control: 0.921 [95% CI: 0.823, 1.031]). Because the particular effect size is dependent on assumptions, we now turn to the empirical evidence supporting the plausibility of our negative control assumptions.

Our first set of bias analyses rely on the assumption that non-firearm suicide serves as a negative control outcome for analyses that assess the effect of CAP laws on firearm suicide. The plausibility of this assumption is supported by consistent and robust findings from individual-level and ecologic studies of firearm ownership in relation to both firearm and non-firearm suicide in conjunction with the fact that CAP laws do not target access to other lethal methods commonly used in suicides [7, 17, 19–22, 26, 27]. For example, when adults become firearm owners, their risk of dying by firearm suicide increases -- as does the risk of dying by firearm suicide for non-firearm owning members of the household, including children [19–21, 23, 28]. However, the rate of non-firearm suicide among firearm owners (and among the non-owner with whom they live) remains unchanged or *declines* minimally–i.e., it does not increase [20–23, 26, 27]. These empirical observations should be borne in mind when weighing the theoretically plausible, but empirically unsubstantiated possibility that CAP laws could hypothetically lead to decreased non-firearm suicide rates by way of indirect effects from suicide contagion. That is, if CAP laws led to decreases in firearm suicides, in time this might lead to decreases in non-firearm suicides by minimizing suicide contagion by all methods. Even so, the non-firearm suicide effect should be much smaller than the effect on firearm suicide.

Our second set of bias analyses rely on the assumption that adults are a negative control population for analyses that assess the effect of CAP laws on adolescent firearm suicide. An argument against this assumption is that changes in storage practices may indeed affect adult suicide rates, even if the storage change is motivated by child access. Nonetheless, the adult firearm suicide rate is largely driven by the firearm suicide rate among men, and men who live in households with firearms are almost always the firearm owner [24, 29, 30]. As such, most adults dying by firearm suicide are unlikely to benefit from any protective effect conferred by CAP laws as they likely have direct and unimpeded access to their own firearms even if stored appropriately [7]. Kivisto et al. as [5] well as Schell et al. [25] found nearly identical protective effects for adult firearm suicide and adolescent firearm suicide, suggesting that at least some amount of bias is occurring even if adults do not meet the precise definition of a negative control population. If estimates of the effect of CAP laws on suicide risk among adult men were available, or even more specifically among handgun owners, these estimates could be incorporated into future bias analyses.

Our bias-adjusted effect estimates are consistent with studies comparing firearm storage practices among firearm owners living in states with versus without CAP laws, and with studies that have documented large inaccuracies in knowledge about CAP laws among firearm owners in states with and without CAP laws [4, 31]. For example, approximately half of firearm owners do not know if they live in a state with a CAP law and firearm owners who live in states with a CAP law are no more likely to secure their firearms than are firearm owners living in states without CAP laws [4]. In addition, locking up firearms may not be as effective a barrier to an adolescent gaining access to a household firearm as many parents think. For example, a recent study found that nearly one-quarter of US adolescents who resided in homes where all firearms were locked could gain access to a loaded household gun in less than 5 min -- and, moreover, that most of their parents report that their child could never gain access without help from an adult [32].

We hope our findings inform future studies that extend what we have presented, such as by adapting our bias-analytic approach to one that names a specific confounder (e.g., firearm prevalence) or considers multiple confounders simultaneously, non-binary confounders, or confounders that differentially affect the outcome of interest [12–14, 33]. We also hope future investigations of the CAP law-adolescent firearm suicide relationship will routinely apply the methodology used here, or extensions of it, when information on our proposed negative controls is available. Doing so will be helpful, even if the investigators do not agree that non-firearm suicide among adolescents and firearm suicide among adults satisfy the assumptions of negative controls, as it would nonetheless provide context for those readers who believe they do. More broadly, we hope that the methodology we used in the current study proves helpful beyond studying CAP laws and will be applied to other firearm-related legislation research [6].

## Conclusion

Considered as a whole, our results suggest that CAP laws have historically had a smaller public health impact on adolescent suicide than has been previously estimated. This does not mean that CAP laws are inherently without merit, currently have no protective effects whatsoever, or that CAP laws may not have larger protective effects in the future. Indeed, the public health impact of CAP laws might increase if awareness of these laws increased, especially if the scientific basis for passing these laws -- that reducing *access* to firearms can prevent suicide deaths, especially among children -- was more broadly acknowledged, discussed, and understood.

## Electronic supplementary materials

Supplementary Material 1

Supplementary Material 2

## Data Availability

No datasets were generated or analysed during the current study.

## Abbreviations

CAP: Child Access Prevention Laws
CI: Confidence Interval
IRR: Incident Rate Ratio
US: United States
