# Supplementary material for: The effect of child access prevention laws on adolescent suicide: a negative control approach
Source: Inj Epidemiol. 2025 Apr 23;12:21. doi: 10.1186/s40621-025-00577-x (PMC12020014; doi:10.1186/s40621-025-00577-x)
Supplement: Supplementary file 1 — Supplementary Material 1 [file 40621_2025_577_MOESM1_ESM.docx]

**Supplementary Material 1 – Full R Code:**

###############################################################################

#' Sean MacAllister

#' Kivisto Bias-adjusted estimates of CAP laws on adolescent firearm suicide rates

#' Originally Authored: April 17th, 2024

###############################################################################

###########################################

#' Load required graphing package

###########################################

pacman::p_load(

ggplot2

)

###############################################################################

###############################################################################

###############################################################################

# ANALYSIS #1

###############################################################################

###############################################################################

###############################################################################

###############################################################################

#' Bias adjustment of adolescent fire-arm suicide rates (FRS) compared to

#' the assumed negative control adolescent non-fire-arm suicide rates (NFS)

###############################################################################

###########################################

#' Input Kivisto's point estimate, lower CI, and upper CI

#' estimates that come from Table S4 as bias parameters for firearm suicide rates

###########################################

frs_irr.pte <- 0.869 # Point estimate

frs_irr.lci <- 0.777 # Lower CI

frs_irr.uci <- 0.973 # Upper CI

###########################################

#' Create First Function For Analysis

###########################################

#' Function for bias-adjusted estimate and CI, assuming non-firearm suicide

#' is a negative control (named biasadj.frs_nfs)

###############################################################################

#' First QBA where we assume NFS is a negative control where NFS IRR = 1

#' and use the bias formula FRS true IRR = FRS estimated IRR/NFS estimated IRR.

#' We use all values contained within the NFS IRR estimated 95% CI to examine

#' how the FRS estimated IRR changes depending on what bias parameter is used.

#' Since the NFS IRR estimated 95% CI contains reasonable estimates, we compare

#' across all values.

###############################################################################

#######################

#' Key for below function:

#' biasadj.frs_nfs = function name

#' adjirr.pte = bias adjusted IRR using the point estimate as bias parameter

#' adjirr.lci = bias adjusted IRR using the lower confidence limit as the bias parameter

#' adjirr.uci = bias adjusted IRR using the upper confidence limit as the bias parameter

#' frs_irr.pte = come from above input as the estimated point estimate bias parameter for firearm suicide

#' frs_irr.lci = come from above input as the estimated lower confidence limit bias parameter for firearm suicide

#' frs_irr.uci = come from above input as the estimated upper confidence limit bias parameter for firearm suicide

#' nfs = NFS suicide IRR negative control bias parameter

#' all equations comes from bias formula of estimated IRR of firearm suicide rates divided by NFS IRR values

#######################

biasadj.frs_nfs <- function(nfs)

{

adjirr.pte <- frs_irr.pte/nfs

adjirr.lci <- frs_irr.lci/nfs

adjirr.uci <- frs_irr.uci/nfs

return(c(nfs,adjirr.pte,adjirr.lci,adjirr.uci))

}

#######################

#' Create blank data set in order to return values using the above function

#######################

dat.frs_nfs <- as.data.frame(matrix(nrow=0,ncol=4))

colnames(dat.frs_nfs) <- c("nfs","adjirr.pte","adjirr.lci","adjirr.uci")

#' Call above function over the range of the NFS IRR estimated 95% CI bias parameter

###' range: 0.843, 0.988 (from Table S4)

biasparam_frs_nfs <- seq(from=0.843,to=0.988,by=0.001) # create vector with all values from NFS IRR estimated 95% CI

for(i in 1:length(biasparam_frs_nfs)) # create loop to be ran through function created earlier for each value from NFS IRR estimated 95% CI

{

dat.frs_nfs[i,] <- biasadj.frs_nfs(biasparam_frs_nfs[i])

}

#' Return dataset to examine bias adjusted estimates

print(dat.frs_nfs)

###############################################################################

#' Graph results

###############################################################################

figure_1 <- ggplot(dat.frs_nfs, aes(x=nfs, y=adjirr.pte)) +

geom_errorbar(aes(y=adjirr.pte, ymin=adjirr.lci, ymax=adjirr.uci), color="grey")+

geom_line()+

geom_point()+

ggtitle("Bias Adjusted Adolescent Firearm Suicide Rate Ratio Assuming Adolescent

Non-Firearm Suicide Rate Ratio as Negative Control")+

theme(plot.title = element_text(hjust = 0.5))+

xlab("Estimated Adolescent Non-Firearm Suicide IRR")+

ylab("Bias-Adjusted Adolescent Firearm Suicide IRR")+

geom_hline(yintercept = 1, color = 'darkgrey')+

geom_vline(xintercept = 0.913,color="azure4")+

theme(panel.background = element_blank(),

strip.background = element_rect(colour=NA, fill=NA),

panel.border = element_rect(fill = NA, color = "black")

)

ggsave("Figure_1.JPEG", plot = figure_1, width = 8, height = 6, dpi = 300)

###############################################################################

###############################################################################

###############################################################################

# ANALYSIS #2

###############################################################################

###############################################################################

###############################################################################

###############################################################################

#' Bias adjustment of child fire-arm suicide rates (CFS) compared to

#' the assumed negative control of adult fire-arm suicide rates (AFS)

###############################################################################

###########################################

#' Input Kivisto's point estimate, lower CI, and upper CI

#' estimates as bias parameters for child firearm suicide rates

###########################################

cfs_irr.pte <- 0.869 # Point estimate

cfs_irr.lci <- 0.777 # Lower CI

cfs_irr.uci <- 0.973 # Upper CI

###########################################

#' Create Second Function For Analysis

###########################################

#' Function for bias-adjusted estimate and CI, assuming adult firearm suicide

#' is a negative control (named biasadj.cfs_afs).

###############################################################################

#' Second QBA where we assume AFS rate is a negative control where AFS IRR = 1

#' and use the bias formula CFS true IRR = CFS estimated IRR/AFS estimated IRR.

#' We use all values contained within the AFS IRR estimated 95% CI to examine

#' how the CFS estimated IRR changes depending on what bias parameter is used.

#' Since the AFS IRR estimated 95% CI contains reasonable estimates, we compare

#' across all values.

###############################################################################

#######################

#' Key for below function:

#' biasadj.cfs_afs = function name

#' adjirr.pte = bias adjusted IRR using the point estimate as bias parameter

#' adjirr.lci = bias adjusted IRR using the lower confidence limit as the bias parameter

#' adjirr.uci = bias adjusted IRR using the upper confidence limit as the bias parameter

#' cfs_irr.pte = come from above input as the estimated point estimate bias parameter for child firearm suicide

#' cfs_irr.lci = come from above input as the estimated lower confidence limit bias parameter for child firearm suicide

#'cfs_ irr.uci = come from above input as the estimated upper confidence limit bias parameter for child firearm suicide

#' afs = afs suicide IRR negative control bias parameter

#' all equations comes from bias formula of estimated IRR of child firearm suicides divided by estimated IRR for adult firearm suicides

#######################

biasadj.cfs_afs <- function(afs)

{

adjirr.pte <- cfs_irr.pte/afs

adjirr.lci <- cfs_irr.lci/afs

adjirr.uci <- cfs_irr.uci/afs

return(c(afs,adjirr.pte,adjirr.lci,adjirr.uci))

}

#######################

#' Create blank data set in order to return values using the above function

#######################

dat.cfs_afs <- as.data.frame(matrix(nrow=0,ncol=4))

colnames(dat.cfs_afs) <- c("afs","adjirr.pte","adjirr.lci","adjirr.uci")

#' Call above function over the range of the AFS IRR estimated 95% CI bias parameter

###' range: 0.923, 0.968

biasparam_cfs_afs <- seq(from=0.922,to=0.968,by=0.001) # create vector with all values from AFS IRR estimated 95% CI

for(i in 1:length(biasparam_cfs_afs)) # create loop to be ran through function created earlier for each value from AFS IRR estimated 95% CI

{

dat.cfs_afs[i,] <- biasadj.cfs_afs(biasparam_cfs_afs[i])

}

#' Return dataset to examine bias adjusted estimates

print(dat.cfs_afs)

###############################################################################

#' Graph results

###############################################################################

figure_2 <- ggplot(dat.cfs_afs, aes(x=afs, y=adjirr.pte)) +

geom_errorbar(aes(y=adjirr.pte, ymin=adjirr.lci, ymax=adjirr.uci), color="grey")+

geom_line()+

geom_point()+

ggtitle("Bias Adjusted Adolescent Firearm Suicide Rate Ratio Assuming Adult

Firearm Suicide Rate Ratio as Negative Control") +

theme(plot.title = element_text(hjust = 0.5))+

xlab("Estimated Adult Firearm Suicide IRR")+

ylab("Bias-Adjusted Adolescent Firearm Suicide IRR")+

geom_hline(yintercept = 1, color = 'darkgrey')+

geom_vline(xintercept = 0.944,color="azure4")+

theme(panel.background = element_blank(),

strip.background = element_rect(colour=NA, fill=NA),

panel.border = element_rect(fill = NA, color = "black")

)

ggsave("Figure_2.JPEG", plot = figure_2, width = 8, height = 6, dpi = 300)

###############################################################################

###############################################################################

###############################################################################

#' Supplemental Material For Other Point Estimates Obtained By Kivisto

###############################################################################

###############################################################################

###############################################################################

##############################################################

#' KEY FOR CAP LAW TYPE NAMING CONVENTION:

#' #1 = Safety lock is required for handguns sold through licensed dealers

#' #2 = Safety lock is required for handguns sold through all dealers

#' #3 = Safety locks must meet state-specified standards or be otherwise approved by the state

#' #4 = All firearms in a household must be stored securely (locked away) at all times

#' #5 = Criminal liability for child access or use

#' #6 = Owner of gun is criminally liable if a gun is not stored properly, regardless of whether a child actually gains access to the gun

#' #7 = Owner of gun is criminally liable if a gun is not stored properly and a child gains access to the gun

#' #8 = Owner of gun is criminally liable if a gun is not stored properly and the child uses or carries the gun

#' #9 = Owner of gun is criminally liable regardless of whether gun is loaded or unloaded

#' #10 = Criminal liability based on the age of the child

#' #11 = Owner of gun is criminally liable if child under age 14 gains access to the gun

#' #12 = Owner of gun is criminally liable if child under age 16 gains access to the gun

#' #13 = Owner of gun is criminally liable if child under age 18 gains access to the gun

##############################################################

###############################################################################

###############################################################################

#' ADOLESCENT FIREARM VS. NON-FIREARM

###############################################################################

###############################################################################

###############################################################################

#' CAP LAW #1: Safety lock is required for handguns sold through licensed dealers

###############################################################################

###########################################

#' Input Kivisto's point estimate, lower CI, and upper CI

#' estimates that come from Table S4 as bias parameters for firearm suicide rates

###########################################

frs_CAP1_irr.pte <- 0.812 # Point estimate

frs_CAP1_irr.lci <- 0.649 # Lower CI

frs_CAP1_irr.uci <- 1.015 # Upper CI

###########################################

#' Create Function For Analysis

###########################################

#######################

#' Key for below function:

#' biasadj.frs_nfs_CAP1 = function name

#' adjirr.pte = bias adjusted IRR using the point estimate as bias parameter

#' adjirr.lci = bias adjusted IRR using the lower confidence limit as the bias parameter

#' adjirr.uci = bias adjusted IRR using the upper confidence limit as the bias parameter

#' dat.frs_nfs_CAP1.pte = come from above input as the estimated point estimate bias parameter for firearm suicide

#' dat.frs_nfs_CAP1.lci = come from above input as the estimated lower confidence limit bias parameter for firearm suicide

#' dat.frs_nfs_CAP1.uci = come from above input as the estimated upper confidence limit bias parameter for firearm suicide

#' nfs = NFS suicide IRR negative control bias parameter

#' all equations comes from bias formula of estimated IRR of firearm suicide rates divided by NFS IRR values

#######################

biasadj.frs_nfs_CAP1 <- function(nfs)

{

adjirr.pte <- frs_CAP1_irr.pte/nfs

adjirr.lci <- frs_CAP1_irr.lci/nfs

adjirr.uci <- frs_CAP1_irr.uci/nfs

return(c(nfs,adjirr.pte,adjirr.lci,adjirr.uci))

}

#######################

#' Create blank data set in order to return values using the above function

#######################

dat.frs_nfs_CAP1 <- as.data.frame(matrix(nrow=0,ncol=4))

colnames(dat.frs_nfs_CAP1) <- c("nfs","adjirr.pte","adjirr.lci","adjirr.uci")

#' Call above function over the range of the NFS IRR estimated 95% CI bias parameter

###' range: 0.760, 1.084 (from Table S4)

biasparam_frs_nfs_CAP1 <- seq(from=0.760,to=1.084,by=0.001) # create vector with all values from NFS IRR estimated 95% CI

for(i in 1:length(biasparam_frs_nfs_CAP1)) # create loop to be ran through function created earlier for each value from NFS IRR estimated 95% CI

{

dat.frs_nfs_CAP1[i,] <- biasadj.frs_nfs_CAP1(biasparam_frs_nfs_CAP1[i])

}

#' Return dataset to examine bias adjusted estimates

print(dat.frs_nfs_CAP1)

###############################################################################

#' Graph results

###############################################################################

ggplot(dat.frs_nfs_CAP1, aes(x=nfs, y=adjirr.pte)) +

geom_errorbar(aes(y=adjirr.pte, ymin=adjirr.lci, ymax=adjirr.uci), color="grey")+

geom_line()+

geom_point()+

ggtitle("Bias Adjusted Adolescent Firearm Suicide Rate Ratio Assuming Adolescent

Non-Firearm Suicide Rate Ratio as Negative Control For CAP Law: Safety Lock is

Required for Handguns Sold Through Licensed Dealers")+

theme(plot.title = element_text(hjust = 0.5))+

xlab("Estimated Adolescent Non-Firearm Suicide IRR")+

ylab("Bias-Adjusted Adolescent Firearm Suicide IRR")+

geom_hline(yintercept = 1, color = 'darkgrey')+

geom_vline(xintercept = 0.908,color="azure4")+

theme(panel.background = element_blank(),

strip.background = element_rect(colour=NA, fill=NA),

panel.border = element_rect(fill = NA, color = "black")

)

ggsave("FS_NFS_CAP_1.JPEG")

###############################################################################

#' CAP LAW #2: Safety lock is required for handguns sold through all dealers

###############################################################################

###########################################

#' Input Kivisto's point estimate, lower CI, and upper CI

#' estimates that come from Table S4 as bias parameters for firearm suicide rates

###########################################

frs_CAP2_irr.pte <- 0.654 # Point estimate

frs_CAP2_irr.lci <- 0.453 # Lower CI

frs_CAP2_irr.uci <- 0.946 # Upper CI

###########################################

#' Create Function For Analysis

###########################################

#######################

#' Key for below function:

#' biasadj.frs_nfs_CAP2 = function name

#' adjirr.pte = bias adjusted IRR using the point estimate as bias parameter

#' adjirr.lci = bias adjusted IRR using the lower confidence limit as the bias parameter

#' adjirr.uci = bias adjusted IRR using the upper confidence limit as the bias parameter

#' dat.frs_nfs_CAP2.pte = come from above input as the estimated point estimate bias parameter for firearm suicide

#' dat.frs_nfs_CAP2.lci = come from above input as the estimated lower confidence limit bias parameter for firearm suicide

#' dat.frs_nfs_CAP2.uci = come from above input as the estimated upper confidence limit bias parameter for firearm suicide

#' nfs = NFS suicide IRR negative control bias parameter

#' all equations comes from bias formula of estimated IRR of firearm suicide rates divided by NFS IRR values

#######################

biasadj.frs_nfs_CAP2 <- function(nfs)

{

adjirr.pte <- frs_CAP2_irr.pte/nfs

adjirr.lci <- frs_CAP2_irr.lci/nfs

adjirr.uci <- frs_CAP2_irr.uci/nfs

return(c(nfs,adjirr.pte,adjirr.lci,adjirr.uci))

}

#######################

#' Create blank data set in order to return values using the above function

#######################

dat.frs_nfs_CAP2 <- as.data.frame(matrix(nrow=0,ncol=4))

colnames(dat.frs_nfs_CAP2) <- c("nfs","adjirr.pte","adjirr.lci","adjirr.uci")

#' Call above function over the range of the NFS IRR estimated 95% CI bias parameter

###' range: 0.617, 1.018 (from Table S4)

biasparam_frs_nfs_CAP2 <- seq(from=0.617,to=1.018,by=0.001) # create vector with all values from NFS IRR estimated 95% CI

for(i in 1:length(biasparam_frs_nfs_CAP2)) # create loop to be ran through function created earlier for each value from NFS IRR estimated 95% CI

{

dat.frs_nfs_CAP2[i,] <- biasadj.frs_nfs_CAP2(biasparam_frs_nfs_CAP2[i])

}

#' Return dataset to examine bias adjusted estimates

print(dat.frs_nfs_CAP2)

###############################################################################

#' Graph results

###############################################################################

ggplot(dat.frs_nfs_CAP2, aes(x=nfs, y=adjirr.pte)) +

geom_errorbar(aes(y=adjirr.pte, ymin=adjirr.lci, ymax=adjirr.uci), color="grey")+

geom_line()+

geom_point()+

ggtitle("Bias Adjusted Adolescent Firearm Suicide Rate Ratio Assuming Adolescent

Non-Firearm Suicide Rate Ratio as Negative Control For CAP Law: Safety Lock is Required

for Handguns Sold Through All Dealers")+

theme(plot.title = element_text(hjust = 0.5))+

xlab("Estimated Adolescent Non-Firearm Suicide IRR")+

ylab("Bias-Adjusted Adolescent Firearm Suicide IRR")+

geom_hline(yintercept = 1, color = 'darkgrey')+

geom_vline(xintercept = 0.793,color="azure4")+

theme(panel.background = element_blank(),

strip.background = element_rect(colour=NA, fill=NA),

panel.border = element_rect(fill = NA, color = "black")

)

ggsave("FS_NFS_CAP_2.JPEG")

###############################################################################

#' CAP LAW #3: Safety locks must meet state-specified standards or be otherwise approved by the state

###############################################################################

###########################################

#' Input Kivisto's point estimate, lower CI, and upper CI

#' estimates that come from Table S4 as bias parameters for firearm suicide rates

###########################################

frs_CAP3_irr.pte <- 0.680 # Point estimate

frs_CAP3_irr.lci <- 0.462 # Lower CI

frs_CAP3_irr.uci <- 1.000 # Upper CI

###########################################

#' Create Function For Analysis

###########################################

#######################

#' Key for below function:

#' biasadj.frs_nfs_CAP3 = function name

#' adjirr.pte = bias adjusted IRR using the point estimate as bias parameter

#' adjirr.lci = bias adjusted IRR using the lower confidence limit as the bias parameter

#' adjirr.uci = bias adjusted IRR using the upper confidence limit as the bias parameter

#' dat.frs_nfs_CAP3.pte = come from above input as the estimated point estimate bias parameter for firearm suicide

#' dat.frs_nfs_CAP3.lci = come from above input as the estimated lower confidence limit bias parameter for firearm suicide

#' dat.frs_nfs_CAP3.uci = come from above input as the estimated upper confidence limit bias parameter for firearm suicide

#' nfs = NFS suicide IRR negative control bias parameter

#' all equations comes from bias formula of estimated IRR of firearm suicide rates divided by NFS IRR values

#######################

biasadj.frs_nfs_CAP3 <- function(nfs)

{

adjirr.pte <- frs_CAP3_irr.pte/nfs

adjirr.lci <- frs_CAP3_irr.lci/nfs

adjirr.uci <- frs_CAP3_irr.uci/nfs

return(c(nfs,adjirr.pte,adjirr.lci,adjirr.uci))

}

#######################

#' Create blank data set in order to return values using the above function

#######################

dat.frs_nfs_CAP3 <- as.data.frame(matrix(nrow=0,ncol=4))

colnames(dat.frs_nfs_CAP3) <- c("nfs","adjirr.pte","adjirr.lci","adjirr.uci")

#' Call above function over the range of the NFS IRR estimated 95% CI bias parameter

###' range: 0.536, 0.9144 (from Table S4)

biasparam_frs_nfs_CAP3 <- seq(from=0.536,to=0.914,by=0.001) # create vector with all values from NFS IRR estimated 95% CI

for(i in 1:length(biasparam_frs_nfs_CAP3)) # create loop to be ran through function created earlier for each value from NFS IRR estimated 95% CI

{

dat.frs_nfs_CAP3[i,] <- biasadj.frs_nfs_CAP3(biasparam_frs_nfs_CAP3[i])

}

#' Return dataset to examine bias adjusted estimates

print(dat.frs_nfs_CAP3)

###############################################################################

#' Graph results

###############################################################################

ggplot(dat.frs_nfs_CAP3, aes(x=nfs, y=adjirr.pte)) +

geom_errorbar(aes(y=adjirr.pte, ymin=adjirr.lci, ymax=adjirr.uci), color="grey")+

geom_line()+

geom_point()+

ggtitle("Bias Adjusted Adolescent Firearm Suicide Rate Ratio Assuming Adolescent

Non-Firearm Suicide Rate Ratio as Negative Control For CAP Law: Safety Locks Must

Meet State-Specified Standards or Be Otherwise Approved by the State")+

theme(plot.title = element_text(hjust = 0.5))+

xlab("Estimated Adolescent Non-Firearm Suicide IRR")+

ylab("Bias-Adjusted Adolescent Firearm Suicide IRR")+

geom_hline(yintercept = 1, color = 'darkgrey')+

geom_vline(xintercept = 0.700,color="azure4")+

theme(panel.background = element_blank(),

strip.background = element_rect(colour=NA, fill=NA),

panel.border = element_rect(fill = NA, color = "black")

)

ggsave("FS_NFS_CAP_3.JPEG")

###############################################################################

#' CAP LAW #4: All firearms in a household must be stored securely (locked away) at all times

###############################################################################

###########################################

#' Input Kivisto's point estimate, lower CI, and upper CI

#' estimates that come from Table S4 as bias parameters for firearm suicide rates

###########################################

frs_CAP4_irr.pte <- 0.534 # Point estimate

frs_CAP4_irr.lci <- 0.240 # Lower CI

frs_CAP4_irr.uci <- 1.189 # Upper CI

###########################################

#' Create Function For Analysis

###########################################

#######################

#' Key for below function:

#' biasadj.frs_nfs_CAP4 = function name

#' adjirr.pte = bias adjusted IRR using the point estimate as bias parameter

#' adjirr.lci = bias adjusted IRR using the lower confidence limit as the bias parameter

#' adjirr.uci = bias adjusted IRR using the upper confidence limit as the bias parameter

#' dat.frs_nfs_CAP4.pte = come from above input as the estimated point estimate bias parameter for firearm suicide

#' dat.frs_nfs_CAP4.lci = come from above input as the estimated lower confidence limit bias parameter for firearm suicide

#' dat.frs_nfs_CAP4.uci = come from above input as the estimated upper confidence limit bias parameter for firearm suicide

#' nfs = NFS suicide IRR negative control bias parameter

#' all equations comes from bias formula of estimated IRR of firearm suicide rates divided by NFS IRR values

#######################

biasadj.frs_nfs_CAP4 <- function(nfs)

{

adjirr.pte <- frs_CAP4_irr.pte/nfs

adjirr.lci <- frs_CAP4_irr.lci/nfs

adjirr.uci <- frs_CAP4_irr.uci/nfs

return(c(nfs,adjirr.pte,adjirr.lci,adjirr.uci))

}

#######################

#' Create blank data set in order to return values using the above function

#######################

dat.frs_nfs_CAP4 <- as.data.frame(matrix(nrow=0,ncol=4))

colnames(dat.frs_nfs_CAP4) <- c("nfs","adjirr.pte","adjirr.lci","adjirr.uci")

#' Call above function over the range of the NFS IRR estimated 95% CI bias parameter

###' range: 0.314, 0.891 (from Table S4)

biasparam_frs_nfs_CAP4 <- seq(from=0.314,to=0.891,by=0.001) # create vector with all values from NFS IRR estimated 95% CI

for(i in 1:length(biasparam_frs_nfs_CAP4)) # create loop to be ran through function created earlier for each value from NFS IRR estimated 95% CI

{

dat.frs_nfs_CAP4[i,] <- biasadj.frs_nfs_CAP4(biasparam_frs_nfs_CAP4[i])

}

#' Return dataset to examine bias adjusted estimates

print(dat.frs_nfs_CAP4)

###############################################################################

#' Graph results

###############################################################################

ggplot(dat.frs_nfs_CAP4, aes(x=nfs, y=adjirr.pte)) +

geom_errorbar(aes(y=adjirr.pte, ymin=adjirr.lci, ymax=adjirr.uci), color="grey")+

geom_line()+

geom_point()+

ggtitle("Bias Adjusted Adolescent Firearm Suicide Rate Ratio Assuming Adolescent

Non-Firearm Suicide Rate Ratio as Negative Control For CAP Law: All Firearms in a Household

Must be Stored Securely (Locked Away) at all Times")+

theme(plot.title = element_text(hjust = 0.5))+

xlab("Estimated Adolescent Non-Firearm Suicide IRR")+

ylab("Bias-Adjusted Adolescent Firearm Suicide IRR")+

geom_hline(yintercept = 1, color = 'darkgrey')+

geom_vline(xintercept = 0.529,color="azure4")+

theme(panel.background = element_blank(),

strip.background = element_rect(colour=NA, fill=NA),

panel.border = element_rect(fill = NA, color = "black")

)

ggsave("FS_NFS_CAP_4.JPEG")

###############################################################################

#' CAP LAW #5: Criminal liability for child access or use

###############################################################################

###########################################

#' Input Kivisto's point estimate, lower CI, and upper CI

#' estimates that come from Table S4 as bias parameters for firearm suicide rates

###########################################

frs_CAP5_irr.pte <- 0.949 # Point estimate

frs_CAP5_irr.lci <- 0.873 # Lower CI

frs_CAP5_irr.uci <- 1.032 # Upper CI

###########################################

#' Create Function For Analysis

###########################################

#######################

#' Key for below function:

#' biasadj.frs_nfs_CAP5 = function name

#' adjirr.pte = bias adjusted IRR using the point estimate as bias parameter

#' adjirr.lci = bias adjusted IRR using the lower confidence limit as the bias parameter

#' adjirr.uci = bias adjusted IRR using the upper confidence limit as the bias parameter

#' dat.frs_nfs_CAP5.pte = come from above input as the estimated point estimate bias parameter for firearm suicide

#' dat.frs_nfs_CAP5.lci = come from above input as the estimated lower confidence limit bias parameter for firearm suicide

#' dat.frs_nfs_CAP5.uci = come from above input as the estimated upper confidence limit bias parameter for firearm suicide

#' nfs = NFS suicide IRR negative control bias parameter

#' all equations comes from bias formula of estimated IRR of firearm suicide rates divided by NFS IRR values

#######################

biasadj.frs_nfs_CAP5 <- function(nfs)

{

adjirr.pte <- frs_CAP5_irr.pte/nfs

adjirr.lci <- frs_CAP5_irr.lci/nfs

adjirr.uci <- frs_CAP5_irr.uci/nfs

return(c(nfs,adjirr.pte,adjirr.lci,adjirr.uci))

}

#######################

#' Create blank data set in order to return values using the above function

#######################

dat.frs_nfs_CAP5 <- as.data.frame(matrix(nrow=0,ncol=4))

colnames(dat.frs_nfs_CAP5) <- c("nfs","adjirr.pte","adjirr.lci","adjirr.uci")

#' Call above function over the range of the NFS IRR estimated 95% CI bias parameter

###' range: 0.929, 1.080 (from Table S4)

biasparam_frs_nfs_CAP5 <- seq(from=0.929,to=1.080,by=0.001) # create vector with all values from NFS IRR estimated 95% CI

for(i in 1:length(biasparam_frs_nfs_CAP5)) # create loop to be ran through function created earlier for each value from NFS IRR estimated 95% CI

{

dat.frs_nfs_CAP5[i,] <- biasadj.frs_nfs_CAP5(biasparam_frs_nfs_CAP5[i])

}

#' Return dataset to examine bias adjusted estimates

print(dat.frs_nfs_CAP5)

###############################################################################

#' Graph results

###############################################################################

ggplot(dat.frs_nfs_CAP5, aes(x=nfs, y=adjirr.pte)) +

geom_errorbar(aes(y=adjirr.pte, ymin=adjirr.lci, ymax=adjirr.uci), color="grey")+

geom_line()+

geom_point()+

ggtitle("Bias Adjusted Adolescent Firearm Suicide Rate Ratio Assuming Adolescent

Non-Firearm Suicide Rate Ratio as Negative Control For CAP Law: Criminal Liability

for Child Access or Use")+

theme(plot.title = element_text(hjust = 0.5))+

xlab("Estimated Adolescent Non-Firearm Suicide IRR")+

ylab("Bias-Adjusted Adolescent Firearm Suicide IRR")+

geom_hline(yintercept = 1, color = 'darkgrey')+

geom_vline(xintercept = 1.001,color="azure4")+

theme(panel.background = element_blank(),

strip.background = element_rect(colour=NA, fill=NA),

panel.border = element_rect(fill = NA, color = "black")

)

ggsave("FS_NFS_CAP_5.JPEG")

###############################################################################

#' CAP LAW #6: Owner of gun is criminally liable if a gun is not stored properly, regardless of whether a child actually gains access to the gun

###############################################################################

###########################################

#' Input Kivisto's point estimate, lower CI, and upper CI

#' estimates that come from Table S4 as bias parameters for firearm suicide rates

###########################################

frs_CAP6_irr.pte <- 1.033 # Point estimate

frs_CAP6_irr.lci <- 0.758 # Lower CI

frs_CAP6_irr.uci <- 1.408 # Upper CI

###########################################

#' Create Function For Analysis

###########################################

#######################

#' Key for below function:

#' biasadj.frs_nfs_CAP6 = function name

#' adjirr.pte = bias adjusted IRR using the point estimate as bias parameter

#' adjirr.lci = bias adjusted IRR using the lower confidence limit as the bias parameter

#' adjirr.uci = bias adjusted IRR using the upper confidence limit as the bias parameter

#' dat.frs_nfs_CAP6.pte = come from above input as the estimated point estimate bias parameter for firearm suicide

#' dat.frs_nfs_CAP6.lci = come from above input as the estimated lower confidence limit bias parameter for firearm suicide

#' dat.frs_nfs_CAP6.uci = come from above input as the estimated upper confidence limit bias parameter for firearm suicide

#' nfs = NFS suicide IRR negative control bias parameter

#' all equations comes from bias formula of estimated IRR of firearm suicide rates divided by NFS IRR values

#######################

biasadj.frs_nfs_CAP6 <- function(nfs)

{

adjirr.pte <- frs_CAP6_irr.pte/nfs

adjirr.lci <- frs_CAP6_irr.lci/nfs

adjirr.uci <- frs_CAP6_irr.uci/nfs

return(c(nfs,adjirr.pte,adjirr.lci,adjirr.uci))

}

#######################

#' Create blank data set in order to return values using the above function

#######################

dat.frs_nfs_CAP6 <- as.data.frame(matrix(nrow=0,ncol=4))

colnames(dat.frs_nfs_CAP6) <- c("nfs","adjirr.pte","adjirr.lci","adjirr.uci")

#' Call above function over the range of the NFS IRR estimated 95% CI bias parameter

###' range: 0.866, 1.450 (from Table S4)

biasparam_frs_nfs_CAP6 <- seq(from=0.866,to=1.450,by=0.001) # create vector with all values from NFS IRR estimated 95% CI

for(i in 1:length(biasparam_frs_nfs_CAP6)) # create loop to be ran through function created earlier for each value from NFS IRR estimated 95% CI

{

dat.frs_nfs_CAP6[i,] <- biasadj.frs_nfs_CAP6(biasparam_frs_nfs_CAP6[i])

}

#' Return dataset to examine bias adjusted estimates

print(dat.frs_nfs_CAP6)

###############################################################################

#' Graph results

###############################################################################

ggplot(dat.frs_nfs_CAP6, aes(x=nfs, y=adjirr.pte)) +

geom_errorbar(aes(y=adjirr.pte, ymin=adjirr.lci, ymax=adjirr.uci), color="grey")+

geom_line()+

geom_point()+

ggtitle("Bias Adjusted Adolescent Firearm Suicide Rate RAtio Assuming Adolescent

Non-Firearm Suicide Rate Ratio as Negative Control For CAP Law: Owner of Gun is Criminally

Liable if a Gun is Not Stored Properly, Regardless of Whether a Child Actually Gains

Access to the Gun")+

theme(plot.title = element_text(hjust = 0.5))+

xlab("Estimated Adolescent Non-Firearm Suicide IRR")+

ylab("Bias-Adjusted Adolescent Firearm Suicide IRR")+

geom_hline(yintercept = 1, color = 'darkgrey')+

geom_vline(xintercept = 1.121,color="azure4")+

theme(panel.background = element_blank(),

strip.background = element_rect(colour=NA, fill=NA),

panel.border = element_rect(fill = NA, color = "black")

)

ggsave("FS_NFS_CAP_6.JPEG")

###############################################################################

#' CAP LAW #7: Owner of gun is criminally liable if a gun is not stored properly and a child gains access to the gun

###############################################################################

###########################################

#' Input Kivisto's point estimate, lower CI, and upper CI

#' estimates that come from Table S4 as bias parameters for firearm suicide rates

###########################################

frs_CAP7_irr.pte <- 0.879 # Point estimate

frs_CAP7_irr.lci <- 0.996 # Lower CI

frs_CAP7_irr.uci <- 1.008 # Upper CI

###########################################

#' Create Function For Analysis

###########################################

#######################

#' Key for below function:

#' biasadj.frs_nfs_CAP7 = function name

#' adjirr.pte = bias adjusted IRR using the point estimate as bias parameter

#' adjirr.lci = bias adjusted IRR using the lower confidence limit as the bias parameter

#' adjirr.uci = bias adjusted IRR using the upper confidence limit as the bias parameter

#' dat.frs_nfs_CAP7.pte = come from above input as the estimated point estimate bias parameter for firearm suicide

#' dat.frs_nfs_CAP7.lci = come from above input as the estimated lower confidence limit bias parameter for firearm suicide

#' dat.frs_nfs_CAP7.uci = come from above input as the estimated upper confidence limit bias parameter for firearm suicide

#' nfs = NFS suicide IRR negative control bias parameter

#' all equations comes from bias formula of estimated IRR of firearm suicide rates divided by NFS IRR values

#######################

biasadj.frs_nfs_CAP7 <- function(nfs)

{

adjirr.pte <- frs_CAP7_irr.pte/nfs

adjirr.lci <- frs_CAP7_irr.lci/nfs

adjirr.uci <- frs_CAP7_irr.uci/nfs

return(c(nfs,adjirr.pte,adjirr.lci,adjirr.uci))

}

#######################

#' Create blank data set in order to return values using the above function

#######################

dat.frs_nfs_CAP7 <- as.data.frame(matrix(nrow=0,ncol=4))

colnames(dat.frs_nfs_CAP7) <- c("nfs","adjirr.pte","adjirr.lci","adjirr.uci")

#' Call above function over the range of the NFS IRR estimated 95% CI bias parameter

###' range: 0.850, 1.286 (from Table S4)

biasparam_frs_nfs_CAP7 <- seq(from=0.850,to=1.286,by=0.001) # create vector with all values from NFS IRR estimated 95% CI

for(i in 1:length(biasparam_frs_nfs_CAP7)) # create loop to be ran through function created earlier for each value from NFS IRR estimated 95% CI

{

dat.frs_nfs_CAP7[i,] <- biasadj.frs_nfs_CAP7(biasparam_frs_nfs_CAP7[i])

}

#' Return dataset to examine bias adjusted estimates

print(dat.frs_nfs_CAP7)

###############################################################################

#' Graph results

###############################################################################

ggplot(dat.frs_nfs_CAP7, aes(x=nfs, y=adjirr.pte)) +

geom_errorbar(aes(y=adjirr.pte, ymin=adjirr.lci, ymax=adjirr.uci), color="grey")+

geom_line()+

geom_point()+

ggtitle("Bias Adjusted Adolescent Firearm Suicide Rate Ratio Assuming Adolescent

Non-Firearm Suicide Rate Ratio as Negative Control For CAP Law: Owner of Gun is Criminally

Liable if a Gun is Not Stored Properly and a Child Gains Access to the Gun")+

theme(plot.title = element_text(hjust = 0.5))+

xlab("Estimated Adolescent Non-Firearm Suicide IRR")+

ylab("Bias-Adjusted Adolescent Firearm Suicide IRR")+

geom_hline(yintercept = 1, color = 'darkgrey')+

geom_vline(xintercept = 1.046,color="azure4")+

theme(panel.background = element_blank(),

strip.background = element_rect(colour=NA, fill=NA),

panel.border = element_rect(fill = NA, color = "black")

)

ggsave("FS_NFS_CAP_7.JPEG")

###############################################################################

#' CAP LAW #8: Owner of gun is criminally liable if a gun is not stored properly and the child uses or carries the gun

###############################################################################

###########################################

#' Input Kivisto's point estimate, lower CI, and upper CI

#' estimates that come from Table S4 as bias parameters for firearm suicide rates

###########################################

frs_CAP8_irr.pte <- 0.918 # Point estimate

frs_CAP8_irr.lci <- 0.768 # Lower CI

frs_CAP8_irr.uci <- 1.098 # Upper CI

###########################################

#' Create Function For Analysis

###########################################

#######################

#' Key for below function:

#' biasadj.frs_nfs_CAP8 = function name

#' adjirr.pte = bias adjusted IRR using the point estimate as bias parameter

#' adjirr.lci = bias adjusted IRR using the lower confidence limit as the bias parameter

#' adjirr.uci = bias adjusted IRR using the upper confidence limit as the bias parameter

#' dat.frs_nfs_CAP8.pte = come from above input as the estimated point estimate bias parameter for firearm suicide

#' dat.frs_nfs_CAP8.lci = come from above input as the estimated lower confidence limit bias parameter for firearm suicide

#' dat.frs_nfs_CAP8.uci = come from above input as the estimated upper confidence limit bias parameter for firearm suicide

#' nfs = NFS suicide IRR negative control bias parameter

#' all equations comes from bias formula of estimated IRR of firearm suicide rates divided by NFS IRR values

#######################

biasadj.frs_nfs_CAP8 <- function(nfs)

{

adjirr.pte <- frs_CAP8_irr.pte/nfs

adjirr.lci <- frs_CAP8_irr.lci/nfs

adjirr.uci <- frs_CAP8_irr.uci/nfs

return(c(nfs,adjirr.pte,adjirr.lci,adjirr.uci))

}

#######################

#' Create blank data set in order to return values using the above function

#######################

dat.frs_nfs_CAP8 <- as.data.frame(matrix(nrow=0,ncol=4))

colnames(dat.frs_nfs_CAP8) <- c("nfs","adjirr.pte","adjirr.lci","adjirr.uci")

#' Call above function over the range of the NFS IRR estimated 95% CI bias parameter

###' range: 0.874, 1.273 (from Table S4)

biasparam_frs_nfs_CAP8 <- seq(from=0.874,to=1.273,by=0.001) # create vector with all values from NFS IRR estimated 95% CI

for(i in 1:length(biasparam_frs_nfs_CAP8)) # create loop to be ran through function created earlier for each value from NFS IRR estimated 95% CI

{

dat.frs_nfs_CAP8[i,] <- biasadj.frs_nfs_CAP8(biasparam_frs_nfs_CAP8[i])

}

#' Return dataset to examine bias adjusted estimates

print(dat.frs_nfs_CAP8)

###############################################################################

#' Graph results

###############################################################################

ggplot(dat.frs_nfs_CAP8, aes(x=nfs, y=adjirr.pte)) +

geom_errorbar(aes(y=adjirr.pte, ymin=adjirr.lci, ymax=adjirr.uci), color="grey")+

geom_line()+

geom_point()+

ggtitle("Bias Adjusted Adolescent Firearm Suicide Rate Ratio Assuming Adolescent

Non-Firearm Suicide Rate Ratio as Negative Control For CAP Law: Owner of Gun is

Criminally Liable if a Gun is Not Stored Properly and the Child Uses or Carries the Gun")+

theme(plot.title = element_text(hjust = 0.5))+

xlab("Estimated Adolescent Non-Firearm Suicide IRR")+

ylab("Bias-Adjusted Adolescent Firearm Suicide IRR")+

geom_hline(yintercept = 1, color = 'darkgrey')+

geom_vline(xintercept = 1.054,color="azure4")+

theme(panel.background = element_blank(),

strip.background = element_rect(colour=NA, fill=NA),

panel.border = element_rect(fill = NA, color = "black")

)

ggsave("FS_NFS_CAP_8.JPEG")

###############################################################################

#' CAP LAW #9: Owner of gun is criminally liable regardless of whether gun is loaded or unloaded

###############################################################################

###########################################

#' Input Kivisto's point estimate, lower CI, and upper CI

#' estimates that come from Table S4 as bias parameters for firearm suicide rates

###########################################

frs_CAP9_irr.pte <- 0.515 # Point estimate

frs_CAP9_irr.lci <- 0.316 # Lower CI

frs_CAP9_irr.uci <- 0.839 # Upper CI

###########################################

#' Create Function For Analysis

###########################################

#######################

#' Key for below function:

#' biasadj.frs_nfs_CAP9 = function name

#' adjirr.pte = bias adjusted IRR using the point estimate as bias parameter

#' adjirr.lci = bias adjusted IRR using the lower confidence limit as the bias parameter

#' adjirr.uci = bias adjusted IRR using the upper confidence limit as the bias parameter

#' dat.frs_nfs_CAP9.pte = come from above input as the estimated point estimate bias parameter for firearm suicide

#' dat.frs_nfs_CAP9.lci = come from above input as the estimated lower confidence limit bias parameter for firearm suicide

#' dat.frs_nfs_CAP9.uci = come from above input as the estimated upper confidence limit bias parameter for firearm suicide

#' nfs = NFS suicide IRR negative control bias parameter

#' all equations comes from bias formula of estimated IRR of firearm suicide rates divided by NFS IRR values

#######################

biasadj.frs_nfs_CAP9 <- function(nfs)

{

adjirr.pte <- frs_CAP9_irr.pte/nfs

adjirr.lci <- frs_CAP9_irr.lci/nfs

adjirr.uci <- frs_CAP9_irr.uci/nfs

return(c(nfs,adjirr.pte,adjirr.lci,adjirr.uci))

}

#######################

#' Create blank data set in order to return values using the above function

#######################

dat.frs_nfs_CAP9 <- as.data.frame(matrix(nrow=0,ncol=4))

colnames(dat.frs_nfs_CAP9) <- c("nfs","adjirr.pte","adjirr.lci","adjirr.uci")

#' Call above function over the range of the NFS IRR estimated 95% CI bias parameter

###' range: 0.434, 0.872 (from Table S4)

biasparam_frs_nfs_CAP9 <- seq(from=0.434,to=0.872,by=0.001) # create vector with all values from NFS IRR estimated 95% CI

for(i in 1:length(biasparam_frs_nfs_CAP9)) # create loop to be ran through function created earlier for each value from NFS IRR estimated 95% CI

{

dat.frs_nfs_CAP9[i,] <- biasadj.frs_nfs_CAP9(biasparam_frs_nfs_CAP9[i])

}

#' Return dataset to examine bias adjusted estimates

print(dat.frs_nfs_CAP9)

###############################################################################

#' Graph results

###############################################################################

ggplot(dat.frs_nfs_CAP9, aes(x=nfs, y=adjirr.pte)) +

geom_errorbar(aes(y=adjirr.pte, ymin=adjirr.lci, ymax=adjirr.uci), color="grey")+

geom_line()+

geom_point()+

ggtitle("Bias Adjusted Adolescent Firearm Suicide Rate Ratio Assuming Adolescent

Non-Firearm Suicide Rate Ratio as Negative Control For CAP Law: Owner of Gun is

Criminally Liable Regardless of Whether Gun is Loaded or Unloaded")+

theme(plot.title = element_text(hjust = 0.5))+

xlab("Estimated Adolescent Non-Firearm Suicide IRR")+

ylab("Bias-Adjusted Adolescent Firearm Suicide IRR")+

geom_hline(yintercept = 1, color = 'darkgrey')+

geom_vline(xintercept = 0.615,color="azure4")+

theme(panel.background = element_blank(),

strip.background = element_rect(colour=NA, fill=NA),

panel.border = element_rect(fill = NA, color = "black")

)

ggsave("FS_NFS_CAP_9.JPEG")

###############################################################################

#' CAP LAW #10: Criminal liability based on the age of the child

###############################################################################

###########################################

#' Input Kivisto's point estimate, lower CI, and upper CI

#' estimates that come from Table S4 as bias parameters for firearm suicide rates

###########################################

frs_CAP10_irr.pte <- 0.943 # Point estimate

frs_CAP10_irr.lci <- 0.866 # Lower CI

frs_CAP10_irr.uci <- 1.025 # Upper CI

###########################################

#' Create Function For Analysis

###########################################

#######################

#' Key for below function:

#' biasadj.frs_nfs_CAP10 = function name

#' adjirr.pte = bias adjusted IRR using the point estimate as bias parameter

#' adjirr.lci = bias adjusted IRR using the lower confidence limit as the bias parameter

#' adjirr.uci = bias adjusted IRR using the upper confidence limit as the bias parameter

#' dat.frs_nfs_CAP10.pte = come from above input as the estimated point estimate bias parameter for firearm suicide

#' dat.frs_nfs_CAP10.lci = come from above input as the estimated lower confidence limit bias parameter for firearm suicide

#' dat.frs_nfs_CAP10.uci = come from above input as the estimated upper confidence limit bias parameter for firearm suicide

#' nfs = NFS suicide IRR negative control bias parameter

#' all equations comes from bias formula of estimated IRR of firearm suicide rates divided by NFS IRR values

#######################

biasadj.frs_nfs_CAP10 <- function(nfs)

{

adjirr.pte <- frs_CAP10_irr.pte/nfs

adjirr.lci <- frs_CAP10_irr.lci/nfs

adjirr.uci <- frs_CAP10_irr.uci/nfs

return(c(nfs,adjirr.pte,adjirr.lci,adjirr.uci))

}

#######################

#' Create blank data set in order to return values using the above function

#######################

dat.frs_nfs_CAP10 <- as.data.frame(matrix(nrow=0,ncol=4))

colnames(dat.frs_nfs_CAP10) <- c("nfs","adjirr.pte","adjirr.lci","adjirr.uci")

#' Call above function over the range of the NFS IRR estimated 95% CI bias parameter

###' range: 0.908, 1.084 (from Table S4)

biasparam_frs_nfs_CAP10 <- seq(from=0.908,to=1.084,by=0.001) # create vector with all values from NFS IRR estimated 95% CI

for(i in 1:length(biasparam_frs_nfs_CAP10)) # create loop to be ran through function created earlier for each value from NFS IRR estimated 95% CI

{

dat.frs_nfs_CAP10[i,] <- biasadj.frs_nfs_CAP10(biasparam_frs_nfs_CAP10[i])

}

#' Return dataset to examine bias adjusted estimates

print(dat.frs_nfs_CAP10)

###############################################################################

#' Graph results

###############################################################################

ggplot(dat.frs_nfs_CAP10, aes(x=nfs, y=adjirr.pte)) +

geom_errorbar(aes(y=adjirr.pte, ymin=adjirr.lci, ymax=adjirr.uci), color="grey")+

geom_line()+

geom_point()+

ggtitle("Bias Adjusted Adolescent Firearm Suicide Rate Ratio Assuming Adolescent

Non-Firearm Suicide Rate Ratio as Negative Control For CAP Law: Criminal Liability

Based on the Age of the Child")+

theme(plot.title = element_text(hjust = 0.5))+

xlab("Estimated Adolescent Non-Firearm Suicide IRR")+

ylab("Bias-Adjusted Adolescent Firearm Suicide IRR")+

geom_hline(yintercept = 1, color = 'darkgrey')+

geom_vline(xintercept = 0.992,color="azure4")+

theme(panel.background = element_blank(),

strip.background = element_rect(colour=NA, fill=NA),

panel.border = element_rect(fill = NA, color = "black")

)

ggsave("FS_NFS_CAP_10.JPEG")

###############################################################################

#' CAP LAW #11: Owner of gun is criminally liable if child under age 14 gains access to the gun

###############################################################################

###########################################

#' Input Kivisto's point estimate, lower CI, and upper CI

#' estimates that come from Table S4 as bias parameters for firearm suicide rates

###########################################

frs_CAP11_irr.pte <- 0.918 # Point estimate

frs_CAP11_irr.lci <- 0.768 # Lower CI

frs_CAP11_irr.uci <- 1.098 # Upper CI

###########################################

#' Create Function For Analysis

###########################################

#######################

#' Key for below function:

#' biasadj.frs_nfs_CAP11 = function name

#' adjirr.pte = bias adjusted IRR using the point estimate as bias parameter

#' adjirr.lci = bias adjusted IRR using the lower confidence limit as the bias parameter

#' adjirr.uci = bias adjusted IRR using the upper confidence limit as the bias parameter

#' dat.frs_nfs_CAP11.pte = come from above input as the estimated point estimate bias parameter for firearm suicide

#' dat.frs_nfs_CAP11.lci = come from above input as the estimated lower confidence limit bias parameter for firearm suicide

#' dat.frs_nfs_CAP11.uci = come from above input as the estimated upper confidence limit bias parameter for firearm suicide

#' nfs = NFS suicide IRR negative control bias parameter

#' all equations comes from bias formula of estimated IRR of firearm suicide rates divided by NFS IRR values

#######################

biasadj.frs_nfs_CAP11 <- function(nfs)

{

adjirr.pte <- frs_CAP11_irr.pte/nfs

adjirr.lci <- frs_CAP11_irr.lci/nfs

adjirr.uci <- frs_CAP11_irr.uci/nfs

return(c(nfs,adjirr.pte,adjirr.lci,adjirr.uci))

}

#######################

#' Create blank data set in order to return values using the above function

#######################

dat.frs_nfs_CAP11 <- as.data.frame(matrix(nrow=0,ncol=4))

colnames(dat.frs_nfs_CAP11) <- c("nfs","adjirr.pte","adjirr.lci","adjirr.uci")

#' Call above function over the range of the NFS IRR estimated 95% CI bias parameter

###' range: 0.874, 1.273 (from Table S4)

biasparam_frs_nfs_CAP11 <- seq(from=0.874,to=1.273,by=0.001) # create vector with all values from NFS IRR estimated 95% CI

for(i in 1:length(biasparam_frs_nfs_CAP11)) # create loop to be ran through function created earlier for each value from NFS IRR estimated 95% CI

{

dat.frs_nfs_CAP11[i,] <- biasadj.frs_nfs_CAP11(biasparam_frs_nfs_CAP11[i])

}

#' Return dataset to examine bias adjusted estimates

print(dat.frs_nfs_CAP11)

###############################################################################

#' Graph results

###############################################################################

ggplot(dat.frs_nfs_CAP11, aes(x=nfs, y=adjirr.pte)) +

geom_errorbar(aes(y=adjirr.pte, ymin=adjirr.lci, ymax=adjirr.uci), color="grey")+

geom_line()+

geom_point()+

ggtitle("Bias Adjusted Adolescent Firearm Suicide Rate Ratio Assuming Adolescent

Non-Firearm Suicide Rate Ratio as Negative Control For CAP Law: Owner of Gun is

Criminally Liable if Child Under Age 14 Gains Access to the Gun")+

theme(plot.title = element_text(hjust = 0.5))+

xlab("Estimated Adolescent Non-Firearm Suicide IRR")+

ylab("Bias-Adjusted Adolescent Firearm Suicide IRR")+

geom_hline(yintercept = 1, color = 'darkgrey')+

geom_vline(xintercept = 1.054,color="azure4")+

theme(panel.background = element_blank(),

strip.background = element_rect(colour=NA, fill=NA),

panel.border = element_rect(fill = NA, color = "black")

)

ggsave("FS_NFS_CAP_11.JPEG")

###############################################################################

#' CAP LAW #12: Owner of gun is criminally liable if child under age 16 gains access to the gun

###############################################################################

###########################################

#' Input Kivisto's point estimate, lower CI, and upper CI

#' estimates that come from Table S4 as bias parameters for firearm suicide rates

###########################################

frs_CAP12_irr.pte <- 0.853 # Point estimate

frs_CAP12_irr.lci <- 0.690 # Lower CI

frs_CAP12_irr.uci <- 1.053 # Upper CI

###########################################

#' Create Function For Analysis

###########################################

#######################

#' Key for below function:

#' biasadj.frs_nfs_CAP12 = function name

#' adjirr.pte = bias adjusted IRR using the point estimate as bias parameter

#' adjirr.lci = bias adjusted IRR using the lower confidence limit as the bias parameter

#' adjirr.uci = bias adjusted IRR using the upper confidence limit as the bias parameter

#' dat.frs_nfs_CAP12.pte = come from above input as the estimated point estimate bias parameter for firearm suicide

#' dat.frs_nfs_CAP12.lci = come from above input as the estimated lower confidence limit bias parameter for firearm suicide

#' dat.frs_nfs_CAP12.uci = come from above input as the estimated upper confidence limit bias parameter for firearm suicide

#' nfs = NFS suicide IRR negative control bias parameter

#' all equations comes from bias formula of estimated IRR of firearm suicide rates divided by NFS IRR values

#######################

biasadj.frs_nfs_CAP12 <- function(nfs)

{

adjirr.pte <- frs_CAP12_irr.pte/nfs

adjirr.lci <- frs_CAP12_irr.lci/nfs

adjirr.uci <- frs_CAP12_irr.uci/nfs

return(c(nfs,adjirr.pte,adjirr.lci,adjirr.uci))

}

#######################

#' Create blank data set in order to return values using the above function

#######################

dat.frs_nfs_CAP12 <- as.data.frame(matrix(nrow=0,ncol=4))

colnames(dat.frs_nfs_CAP12) <- c("nfs","adjirr.pte","adjirr.lci","adjirr.uci")

#' Call above function over the range of the NFS IRR estimated 95% CI bias parameter

###' range: 0.751, 1.173 (from Table S4)

biasparam_frs_nfs_CAP12 <- seq(from=0.751,to=1.173,by=0.001) # create vector with all values from NFS IRR estimated 95% CI

for(i in 1:length(biasparam_frs_nfs_CAP12)) # create loop to be ran through function created earlier for each value from NFS IRR estimated 95% CI

{

dat.frs_nfs_CAP12[i,] <- biasadj.frs_nfs_CAP12(biasparam_frs_nfs_CAP12[i])

}

#' Return dataset to examine bias adjusted estimates

print(dat.frs_nfs_CAP12)

###############################################################################

#' Graph results

###############################################################################

ggplot(dat.frs_nfs_CAP12, aes(x=nfs, y=adjirr.pte)) +

geom_errorbar(aes(y=adjirr.pte, ymin=adjirr.lci, ymax=adjirr.uci), color="grey")+

geom_line()+

geom_point()+

ggtitle("Bias Adjusted Adolescent Firearm Suicide Rate Ratio Assuming Adolescent

Non-Firearm Suicide Rate Ratio as Negative Control For CAP Law: Owner of Gun is

Criminally Liable if Child Under Age 16 Gains Access to the Gun")+

theme(plot.title = element_text(hjust = 0.5))+

xlab("Estimated Adolescent Non-Firearm Suicide IRR")+

ylab("Bias-Adjusted Adolescent Firearm Suicide IRR")+

geom_hline(yintercept = 1, color = 'darkgrey')+

geom_vline(xintercept = 0.949,color="azure4")+

theme(panel.background = element_blank(),

strip.background = element_rect(colour=NA, fill=NA),

panel.border = element_rect(fill = NA, color = "black")

)

ggsave("FS_NFS_CAP_12.JPEG")

###############################################################################

#' CAP LAW #13: Owner of gun is criminally liable if child under age 18 gains access to the gun

###############################################################################

###########################################

#' Input Kivisto's point estimate, lower CI, and upper CI

#' estimates that come from Table S4 as bias parameters for firearm suicide rates

###########################################

frs_CAP13_irr.pte <- 0.824 # Point estimate

frs_CAP13_irr.lci <- 0.605 # Lower CI

frs_CAP13_irr.uci <- 1.121 # Upper CI

###########################################

#' Create Function For Analysis

###########################################

#######################

#' Key for below function:

#' biasadj.frs_nfs_CAP13 = function name

#' adjirr.pte = bias adjusted IRR using the point estimate as bias parameter

#' adjirr.lci = bias adjusted IRR using the lower confidence limit as the bias parameter

#' adjirr.uci = bias adjusted IRR using the upper confidence limit as the bias parameter

#' dat.frs_nfs_CAP13.pte = come from above input as the estimated point estimate bias parameter for firearm suicide

#' dat.frs_nfs_CAP13.lci = come from above input as the estimated lower confidence limit bias parameter for firearm suicide

#' dat.frs_nfs_CAP13.uci = come from above input as the estimated upper confidence limit bias parameter for firearm suicide

#' nfs = NFS suicide IRR negative control bias parameter

#' all equations comes from bias formula of estimated IRR of firearm suicide rates divided by NFS IRR values

#######################

biasadj.frs_nfs_CAP13 <- function(nfs)

{

adjirr.pte <- frs_CAP13_irr.pte/nfs

adjirr.lci <- frs_CAP13_irr.lci/nfs

adjirr.uci <- frs_CAP13_irr.uci/nfs

return(c(nfs,adjirr.pte,adjirr.lci,adjirr.uci))

}

#######################

#' Create blank data set in order to return values using the above function

#######################

dat.frs_nfs_CAP13 <- as.data.frame(matrix(nrow=0,ncol=4))

colnames(dat.frs_nfs_CAP13) <- c("nfs","adjirr.pte","adjirr.lci","adjirr.uci")

#' Call above function over the range of the NFS IRR estimated 95% CI bias parameter

###' range: 0.639, 1.214 (from Table S4)

biasparam_frs_nfs_CAP13 <- seq(from=0.639,to=1.214,by=0.001) # create vector with all values from NFS IRR estimated 95% CI

for(i in 1:length(biasparam_frs_nfs_CAP13)) # create loop to be ran through function created earlier for each value from NFS IRR estimated 95% CI

{

dat.frs_nfs_CAP13[i,] <- biasadj.frs_nfs_CAP13(biasparam_frs_nfs_CAP13[i])

}

#' Return dataset to examine bias adjusted estimates

print(dat.frs_nfs_CAP13)

###############################################################################

#' Graph results

###############################################################################

ggplot(dat.frs_nfs_CAP13, aes(x=nfs, y=adjirr.pte)) +

geom_errorbar(aes(y=adjirr.pte, ymin=adjirr.lci, ymax=adjirr.uci), color="grey")+

geom_line()+

geom_point()+

ggtitle("Bias Adjusted Adolescent Firearm Suicide Rate Ratio Assuming Adolescent

Non-Firearm Suicide Rate Ratio as Negative Control For CAP Law: Owner of Gun is

Criminally Liable if Child Under Age 18 Gains Access to the Gun")+

theme(plot.title = element_text(hjust = 0.5))+

xlab("Estimated Adolescent Non-Firearm Suicide IRR")+

ylab("Bias-Adjusted Adolescent Firearm Suicide IRR")+

geom_hline(yintercept = 1, color = 'darkgrey')+

geom_vline(xintercept = 0.881,color="azure4")+

theme(panel.background = element_blank(),

strip.background = element_rect(colour=NA, fill=NA),

panel.border = element_rect(fill = NA, color = "black")

)

ggsave("FS_NFS_CAP_13.JPEG")

###############################################################################

###############################################################################

#' ADULT FIREARM VS. CHILD FIREARM

###############################################################################

###############################################################################

###############################################################################

#' CAP LAW #1: Safety lock is required for handguns sold through licensed dealers

###############################################################################

###########################################

#' Input Kivisto's point estimate, lower CI, and upper CI

#' estimates as bias parameters for child firearm suicide rates

###########################################

cfs_irr_CAP1.pte <- 0.812 # Point estimate

cfs_irr_CAP1.lci <- 0.649 # Lower CI

cfs_irr_CAP1.uci <- 1.015 # Upper CI

###########################################

#' Create Second Function For Analysis

###########################################

#######################

#' Key for below function:

#' biasadj.cfs_afs_CAP1 = function name

#' adjirr.pte = bias adjusted IRR using the point estimate as bias parameter

#' adjirr.lci = bias adjusted IRR using the lower confidence limit as the bias parameter

#' adjirr.uci = bias adjusted IRR using the upper confidence limit as the bias parameter

#' cfs_irr_CAP1.pte = come from above input as the estimated point estimate bias parameter for child firearm suicide

#' cfs_irr_CAP1.lci = come from above input as the estimated lower confidence limit bias parameter for child firearm suicide

#'cfs_ irr_CAP1.uci = come from above input as the estimated upper confidence limit bias parameter for child firearm suicide

#' afs = afs suicide IRR negative control bias parameter

#' all equations comes from bias formula of estimated IRR of child firearm suicides divided by estimated IRR for adult firearm suicides

#######################

biasadj.cfs_afs_CAP1 <- function(afs)

{

adjirr.pte <- cfs_irr_CAP1.pte/afs

adjirr.lci <- cfs_irr_CAP1.lci/afs

adjirr.uci <- cfs_irr_CAP1.uci/afs

return(c(afs,adjirr.pte,adjirr.lci,adjirr.uci))

}

#######################

#' Create blank data set in order to return values using the above function

#######################

dat.cfs_afs_CAP1 <- as.data.frame(matrix(nrow=0,ncol=4))

colnames(dat.cfs_afs_CAP1) <- c("afs","adjirr.pte","adjirr.lci","adjirr.uci")

#' Call above function over the range of the AFS IRR estimated 95% CI bias parameter

###' range: 0.859, 0.942

biasparam_cfs_afs_CAP1 <- seq(from=0.859,to=0.942,by=0.001) # create vector with all values from AFS IRR estimated 95% CI

for(i in 1:length(biasparam_cfs_afs_CAP1)) # create loop to be ran through function created earlier for each value from AFS IRR estimated 95% CI

{

dat.cfs_afs_CAP1[i,] <- biasadj.cfs_afs_CAP1(biasparam_cfs_afs_CAP1[i])

}

#' Return dataset to examine bias adjusted estimates

print(dat.cfs_afs_CAP1)

###############################################################################

#' Graph results

###############################################################################

ggplot(dat.cfs_afs_CAP1, aes(x=afs, y=adjirr.pte)) +

geom_errorbar(aes(y=adjirr.pte, ymin=adjirr.lci, ymax=adjirr.uci), color="grey")+

geom_line()+

geom_point()+

ggtitle("Bias Adjusted Adolescent Firearm Suicide Rate Ratio Assuming Adult

Firearm Suicide Rate Ratio as Negative Control For CAP Law: Safety Lock

is Required for Handguns Sold Through Licensed Dealers") +

theme(plot.title = element_text(hjust = 0.5))+

xlab("Estimated Adult Firearm Suicide IRR")+

ylab("Bias-Adjusted Adolescent Firearm Suicide IRR")+

geom_hline(yintercept = 1, color = 'darkgrey')+

geom_vline(xintercept = 0.899,color="azure4")+

theme(panel.background = element_blank(),

strip.background = element_rect(colour=NA, fill=NA),

panel.border = element_rect(fill = NA, color = "black")

)

ggsave("AFS_CFS_CAP1.JPEG")

###############################################################################

#' CAP LAW #2: Safety lock is required for handguns sold through all dealers

###############################################################################

###########################################

#' Input Kivisto's point estimate, lower CI, and upper CI

#' estimates as bias parameters for child firearm suicide rates

###########################################

cfs_irr_CAP2.pte <- 0.654 # Point estimate

cfs_irr_CAP2.lci <- 0.453 # Lower CI

cfs_irr_CAP2.uci <- 0.946 # Upper CI

###########################################

#' Create Second Function For Analysis

###########################################

#######################

#' Key for below function:

#' biasadj.cfs_afs_CAP2 = function name

#' adjirr.pte = bias adjusted IRR using the point estimate as bias parameter

#' adjirr.lci = bias adjusted IRR using the lower confidence limit as the bias parameter

#' adjirr.uci = bias adjusted IRR using the upper confidence limit as the bias parameter

#' cfs_irr_CAP2.pte = come from above input as the estimated point estimate bias parameter for child firearm suicide

#' cfs_irr_CAP2.lci = come from above input as the estimated lower confidence limit bias parameter for child firearm suicide

#'cfs_ irr_CAP2.uci = come from above input as the estimated upper confidence limit bias parameter for child firearm suicide

#' afs = afs suicide IRR negative control bias parameter

#' all equations comes from bias formula of estimated IRR of child firearm suicides divided by estimated IRR for adult firearm suicides

#######################

biasadj.cfs_afs_CAP2 <- function(afs)

{

adjirr.pte <- cfs_irr_CAP2.pte/afs

adjirr.lci <- cfs_irr_CAP2.lci/afs

adjirr.uci <- cfs_irr_CAP2.uci/afs

return(c(afs,adjirr.pte,adjirr.lci,adjirr.uci))

}

#######################

#' Create blank data set in order to return values using the above function

#######################

dat.cfs_afs_CAP2 <- as.data.frame(matrix(nrow=0,ncol=4))

colnames(dat.cfs_afs_CAP2) <- c("afs","adjirr.pte","adjirr.lci","adjirr.uci")

#' Call above function over the range of the AFS IRR estimated 95% CI bias parameter

###' range: 0.821, 0.952

biasparam_cfs_afs_CAP2 <- seq(from=0.821,to=0.952,by=0.001) # create vector with all values from AFS IRR estimated 95% CI

for(i in 1:length(biasparam_cfs_afs_CAP2)) # create loop to be ran through function created earlier for each value from AFS IRR estimated 95% CI

{

dat.cfs_afs_CAP2[i,] <- biasadj.cfs_afs_CAP2(biasparam_cfs_afs_CAP2[i])

}

#' Return dataset to examine bias adjusted estimates

print(dat.cfs_afs_CAP2)

###############################################################################

#' Graph results

###############################################################################

ggplot(dat.cfs_afs_CAP2, aes(x=afs, y=adjirr.pte)) +

geom_errorbar(aes(y=adjirr.pte, ymin=adjirr.lci, ymax=adjirr.uci), color="grey")+

geom_line()+

geom_point()+

ggtitle("Bias Adjusted Adolescent Firearm Suicide Rate Ratio Assuming Adult

Firearm Suicide Rate Ratio as Negative Control For CAP Law: Safety Lock is

Required for Handguns Sold Through All Dealers") +

theme(plot.title = element_text(hjust = 0.5))+

xlab("Estimated Adult Firearm Suicide IRR")+

ylab("Bias-Adjusted Adolescent Firearm Suicide IRR")+

geom_hline(yintercept = 1, color = 'darkgrey')+

geom_vline(xintercept = 0.884,color="azure4")+

theme(panel.background = element_blank(),

strip.background = element_rect(colour=NA, fill=NA),

panel.border = element_rect(fill = NA, color = "black")

)

ggsave("AFS_CFS_CAP2.JPEG")

###############################################################################

#' CAP LAW #3: Safety locks must meet state-specified standards or be otherwise approved by the state

###############################################################################

###########################################

#' Input Kivisto's point estimate, lower CI, and upper CI

#' estimates as bias parameters for child firearm suicide rates

###########################################

cfs_irr_CAP3.pte <- 0.680 # Point estimate

cfs_irr_CAP3.lci <- 0.462 # Lower CI

cfs_irr_CAP3.uci <- 1.000 # Upper CI

###########################################

#' Create Second Function For Analysis

###########################################

#######################

#' Key for below function:

#' biasadj.cfs_afs_CAP3 = function name

#' adjirr.pte = bias adjusted IRR using the point estimate as bias parameter

#' adjirr.lci = bias adjusted IRR using the lower confidence limit as the bias parameter

#' adjirr.uci = bias adjusted IRR using the upper confidence limit as the bias parameter

#' cfs_irr_CAP3.pte = come from above input as the estimated point estimate bias parameter for child firearm suicide

#' cfs_irr_CAP3.lci = come from above input as the estimated lower confidence limit bias parameter for child firearm suicide

#'cfs_ irr_CAP3.uci = come from above input as the estimated upper confidence limit bias parameter for child firearm suicide

#' afs = afs suicide IRR negative control bias parameter

#' all equations comes from bias formula of estimated IRR of child firearm suicides divided by estimated IRR for adult firearm suicides

#######################

biasadj.cfs_afs_CAP3 <- function(afs)

{

adjirr.pte <- cfs_irr_CAP3.pte/afs

adjirr.lci <- cfs_irr_CAP3.lci/afs

adjirr.uci <- cfs_irr_CAP3.uci/afs

return(c(afs,adjirr.pte,adjirr.lci,adjirr.uci))

}

#######################

#' Create blank data set in order to return values using the above function

#######################

dat.cfs_afs_CAP3 <- as.data.frame(matrix(nrow=0,ncol=4))

colnames(dat.cfs_afs_CAP3) <- c("afs","adjirr.pte","adjirr.lci","adjirr.uci")

#' Call above function over the range of the AFS IRR estimated 95% CI bias parameter

###' range: 0.676, 0.948

biasparam_cfs_afs_CAP3 <- seq(from=0.676,to=0.948,by=0.001) # create vector with all values from AFS IRR estimated 95% CI

for(i in 1:length(biasparam_cfs_afs_CAP3)) # create loop to be ran through function created earlier for each value from AFS IRR estimated 95% CI

{

dat.cfs_afs_CAP3[i,] <- biasadj.cfs_afs_CAP3(biasparam_cfs_afs_CAP3[i])

}

#' Return dataset to examine bias adjusted estimates

print(dat.cfs_afs_CAP3)

###############################################################################

#' Graph results

###############################################################################

ggplot(dat.cfs_afs_CAP3, aes(x=afs, y=adjirr.pte)) +

geom_errorbar(aes(y=adjirr.pte, ymin=adjirr.lci, ymax=adjirr.uci), color="grey")+

geom_line()+

geom_point()+

ggtitle("Bias Adjusted Adolescent Firearm Suicide Rate Ratio Assuming Adult

Firearm Suicide Rate Ratio as Negative Control For CAP Law: Safety Locks Must Meet

State-Specified Standards or be Otherwise Approved by the State") +

theme(plot.title = element_text(hjust = 0.5))+

xlab("Estimated Adult Firearm Suicide IRR")+

ylab("Bias-Adjusted Adolescent Firearm Suicide IRR")+

geom_hline(yintercept = 1, color = 'darkgrey')+

geom_vline(xintercept = 0.800,color="azure4")+

theme(panel.background = element_blank(),

strip.background = element_rect(colour=NA, fill=NA),

panel.border = element_rect(fill = NA, color = "black")

)

ggsave("AFS_CFS_CAP3.JPEG")

###############################################################################

#' CAP LAW #4: All firearms in a household must be stored securely (locked away) at all times

###############################################################################

###########################################

#' Input Kivisto's point estimate, lower CI, and upper CI

#' estimates as bias parameters for child firearm suicide rates

###########################################

cfs_irr_CAP4.pte <- 0.534 # Point estimate

cfs_irr_CAP4.lci <- 0.240 # Lower CI

cfs_irr_CAP4.uci <- 1.189 # Upper CI

###########################################

#' Create Second Function For Analysis

###########################################

#######################

#' Key for below function:

#' biasadj.cfs_afs_CAP4 = function name

#' adjirr.pte = bias adjusted IRR using the point estimate as bias parameter

#' adjirr.lci = bias adjusted IRR using the lower confidence limit as the bias parameter

#' adjirr.uci = bias adjusted IRR using the upper confidence limit as the bias parameter

#' cfs_irr_CAP4.pte = come from above input as the estimated point estimate bias parameter for child firearm suicide

#' cfs_irr_CAP4.lci = come from above input as the estimated lower confidence limit bias parameter for child firearm suicide

#'cfs_ irr_CAP4.uci = come from above input as the estimated upper confidence limit bias parameter for child firearm suicide

#' afs = afs suicide IRR negative control bias parameter

#' all equations comes from bias formula of estimated IRR of child firearm suicides divided by estimated IRR for adult firearm suicides

#######################

biasadj.cfs_afs_CAP4 <- function(afs)

{

adjirr.pte <- cfs_irr_CAP4.pte/afs

adjirr.lci <- cfs_irr_CAP4.lci/afs

adjirr.uci <- cfs_irr_CAP4.uci/afs

return(c(afs,adjirr.pte,adjirr.lci,adjirr.uci))

}

#######################

#' Create blank data set in order to return values using the above function

#######################

dat.cfs_afs_CAP4 <- as.data.frame(matrix(nrow=0,ncol=4))

colnames(dat.cfs_afs_CAP4) <- c("afs","adjirr.pte","adjirr.lci","adjirr.uci")

#' Call above function over the range of the AFS IRR estimated 95% CI bias parameter

###' range: 0.810, 0.967

biasparam_cfs_afs_CAP4 <- seq(from=0.810,to=0.967,by=0.001) # create vector with all values from AFS IRR estimated 95% CI

for(i in 1:length(biasparam_cfs_afs_CAP4)) # create loop to be ran through function created earlier for each value from AFS IRR estimated 95% CI

{

dat.cfs_afs_CAP4[i,] <- biasadj.cfs_afs_CAP4(biasparam_cfs_afs_CAP4[i])

}

#' Return dataset to examine bias adjusted estimates

print(dat.cfs_afs_CAP4)

###############################################################################

#' Graph results

###############################################################################

ggplot(dat.cfs_afs_CAP4, aes(x=afs, y=adjirr.pte)) +

geom_errorbar(aes(y=adjirr.pte, ymin=adjirr.lci, ymax=adjirr.uci), color="grey")+

geom_line()+

geom_point()+

ggtitle("Bias Adjusted Adolescent Firearm Suicide Rate Ratio Assuming Adult

Firearm Suicide Rate Ratio as Negative Control For CAP Law: All Firearms

in a Household Must be Stored Securely (Locked Away) at All Times") +

theme(plot.title = element_text(hjust = 0.5))+

xlab("Estimated Adult Firearm Suicide IRR")+

ylab("Bias-Adjusted Adolescent Firearm Suicide IRR")+

geom_hline(yintercept = 1, color = 'darkgrey')+

geom_vline(xintercept = 0.885,color="azure4")+

theme(panel.background = element_blank(),

strip.background = element_rect(colour=NA, fill=NA),

panel.border = element_rect(fill = NA, color = "black")

)

ggsave("AFS_CFS_CAP4.JPEG")

###############################################################################

#' CAP LAW #5: Criminal liability for child access or use

###############################################################################

###########################################

#' Input Kivisto's point estimate, lower CI, and upper CI

#' estimates as bias parameters for child firearm suicide rates

###########################################

cfs_irr_CAP5.pte <- 0.949 # Point estimate

cfs_irr_CAP5.lci <- 0.873 # Lower CI

cfs_irr_CAP5.uci <- 1.032 # Upper CI

###########################################

#' Create Second Function For Analysis

###########################################

#######################

#' Key for below function:

#' biasadj.cfs_afs_CAP5 = function name

#' adjirr.pte = bias adjusted IRR using the point estimate as bias parameter

#' adjirr.lci = bias adjusted IRR using the lower confidence limit as the bias parameter

#' adjirr.uci = bias adjusted IRR using the upper confidence limit as the bias parameter

#' cfs_irr_CAP5.pte = come from above input as the estimated point estimate bias parameter for child firearm suicide

#' cfs_irr_CAP5.lci = come from above input as the estimated lower confidence limit bias parameter for child firearm suicide

#'cfs_ irr_CAP5.uci = come from above input as the estimated upper confidence limit bias parameter for child firearm suicide

#' afs = afs suicide IRR negative control bias parameter

#' all equations comes from bias formula of estimated IRR of child firearm suicides divided by estimated IRR for adult firearm suicides

#######################

biasadj.cfs_afs_CAP5 <- function(afs)

{

adjirr.pte <- cfs_irr_CAP5.pte/afs

adjirr.lci <- cfs_irr_CAP5.lci/afs

adjirr.uci <- cfs_irr_CAP5.uci/afs

return(c(afs,adjirr.pte,adjirr.lci,adjirr.uci))

}

#######################

#' Create blank data set in order to return values using the above function

#######################

dat.cfs_afs_CAP5 <- as.data.frame(matrix(nrow=0,ncol=4))

colnames(dat.cfs_afs_CAP5) <- c("afs","adjirr.pte","adjirr.lci","adjirr.uci")

#' Call above function over the range of the AFS IRR estimated 95% CI bias parameter

###' range: 0.964, 1.001

biasparam_cfs_afs_CAP5 <- seq(from=0.964,to=1.001,by=0.001) # create vector with all values from AFS IRR estimated 95% CI

for(i in 1:length(biasparam_cfs_afs_CAP5)) # create loop to be ran through function created earlier for each value from AFS IRR estimated 95% CI

{

dat.cfs_afs_CAP5[i,] <- biasadj.cfs_afs_CAP5(biasparam_cfs_afs_CAP5[i])

}

#' Return dataset to examine bias adjusted estimates

print(dat.cfs_afs_CAP5)

###############################################################################

#' Graph results

###############################################################################

ggplot(dat.cfs_afs_CAP5, aes(x=afs, y=adjirr.pte)) +

geom_errorbar(aes(y=adjirr.pte, ymin=adjirr.lci, ymax=adjirr.uci), color="grey")+

geom_line()+

geom_point()+

ggtitle("Bias Adjusted Adolescent Firearm Suicide Rate Ratio Assuming Adult

Firearm Suicide Rate Ratio as Negative Control For CAP Law: Criminal Liability

for Child Access or Use") +

theme(plot.title = element_text(hjust = 0.5))+

xlab("Estimated Adult Firearm Suicide IRR")+

ylab("Bias-Adjusted Adolescent Firearm Suicide IRR")+

geom_hline(yintercept = 1, color = 'darkgrey')+

geom_vline(xintercept = 0.983,color="azure4")+

theme(panel.background = element_blank(),

strip.background = element_rect(colour=NA, fill=NA),

panel.border = element_rect(fill = NA, color = "black")

)

ggsave("AFS_CFS_CAP5.JPEG")

###############################################################################

#' CAP LAW #6: Owner of gun is criminally liable if a gun is not stored properly, regardless of whether a child actually gains access to the gun

###############################################################################

###########################################

#' Input Kivisto's point estimate, lower CI, and upper CI

#' estimates as bias parameters for child firearm suicide rates

###########################################

cfs_irr_CAP6.pte <- 1.033 # Point estimate

cfs_irr_CAP6.lci <- 0.758 # Lower CI

cfs_irr_CAP6.uci <- 1.408 # Upper CI

###########################################

#' Create Second Function For Analysis

###########################################

#######################

#' Key for below function:

#' biasadj.cfs_afs_CAP6 = function name

#' adjirr.pte = bias adjusted IRR using the point estimate as bias parameter

#' adjirr.lci = bias adjusted IRR using the lower confidence limit as the bias parameter

#' adjirr.uci = bias adjusted IRR using the upper confidence limit as the bias parameter

#' cfs_irr_CAP6.pte = come from above input as the estimated point estimate bias parameter for child firearm suicide

#' cfs_irr_CAP6.lci = come from above input as the estimated lower confidence limit bias parameter for child firearm suicide

#'cfs_ irr_CAP6.uci = come from above input as the estimated upper confidence limit bias parameter for child firearm suicide

#' afs = afs suicide IRR negative control bias parameter

#' all equations comes from bias formula of estimated IRR of child firearm suicides divided by estimated IRR for adult firearm suicides

#######################

biasadj.cfs_afs_CAP6 <- function(afs)

{

adjirr.pte <- cfs_irr_CAP6.pte/afs

adjirr.lci <- cfs_irr_CAP6.lci/afs

adjirr.uci <- cfs_irr_CAP6.uci/afs

return(c(afs,adjirr.pte,adjirr.lci,adjirr.uci))

}

#######################

#' Create blank data set in order to return values using the above function

#######################

dat.cfs_afs_CAP6 <- as.data.frame(matrix(nrow=0,ncol=4))

colnames(dat.cfs_afs_CAP6) <- c("afs","adjirr.pte","adjirr.lci","adjirr.uci")

#' Call above function over the range of the AFS IRR estimated 95% CI bias parameter

###' range: 0.923, 1.070

biasparam_cfs_afs_CAP6 <- seq(from=0.923,to=1.070,by=0.001) # create vector with all values from AFS IRR estimated 95% CI

for(i in 1:length(biasparam_cfs_afs_CAP6)) # create loop to be ran through function created earlier for each value from AFS IRR estimated 95% CI

{

dat.cfs_afs_CAP6[i,] <- biasadj.cfs_afs_CAP6(biasparam_cfs_afs_CAP6[i])

}

#' Return dataset to examine bias adjusted estimates

print(dat.cfs_afs_CAP6)

###############################################################################

#' Graph results

###############################################################################

ggplot(dat.cfs_afs_CAP6, aes(x=afs, y=adjirr.pte)) +

geom_errorbar(aes(y=adjirr.pte, ymin=adjirr.lci, ymax=adjirr.uci), color="grey")+

geom_line()+

geom_point()+

ggtitle("Bias Adjusted Adolescent Firearm Suicide Rate Ratio Assuming Adult

Firearm Suicide Rate Ratio as Negative Control For CAP Law: Owner of Gun is Criminally

Liable if a Gun is Not Stored Properly, Regardless of Whether a Child Actually Gains

Access to the Gun") +

theme(plot.title = element_text(hjust = 0.5))+

xlab("Estimated Adult Firearm Suicide IRR")+

ylab("Bias-Adjusted Adolescent Firearm Suicide IRR")+

geom_hline(yintercept = 1, color = 'darkgrey')+

geom_vline(xintercept = 0.994,color="azure4")+

theme(panel.background = element_blank(),

strip.background = element_rect(colour=NA, fill=NA),

panel.border = element_rect(fill = NA, color = "black")

)

ggsave("AFS_CFS_CAP6.JPEG")

###############################################################################

#' CAP LAW #7: Owner of gun is criminally liable if a gun is not stored properly and a child gains access to the gun

###############################################################################

###########################################

#' Input Kivisto's point estimate, lower CI, and upper CI

#' estimates as bias parameters for child firearm suicide rates

###########################################

cfs_irr_CAP7.pte <- 0.879 # Point estimate

cfs_irr_CAP7.lci <- 0.996 # Lower CI

cfs_irr_CAP7.uci <- 1.008 # Upper CI

###########################################

#' Create Second Function For Analysis

###########################################

#######################

#' Key for below function:

#' biasadj.cfs_afs_CAP7 = function name

#' adjirr.pte = bias adjusted IRR using the point estimate as bias parameter

#' adjirr.lci = bias adjusted IRR using the lower confidence limit as the bias parameter

#' adjirr.uci = bias adjusted IRR using the upper confidence limit as the bias parameter

#' cfs_irr_CAP7.pte = come from above input as the estimated point estimate bias parameter for child firearm suicide

#' cfs_irr_CAP7.lci = come from above input as the estimated lower confidence limit bias parameter for child firearm suicide

#'cfs_ irr_CAP7.uci = come from above input as the estimated upper confidence limit bias parameter for child firearm suicide

#' afs = afs suicide IRR negative control bias parameter

#' all equations comes from bias formula of estimated IRR of child firearm suicides divided by estimated IRR for adult firearm suicides

#######################

biasadj.cfs_afs_CAP7 <- function(afs)

{

adjirr.pte <- cfs_irr_CAP7.pte/afs

adjirr.lci <- cfs_irr_CAP7.lci/afs

adjirr.uci <- cfs_irr_CAP7.uci/afs

return(c(afs,adjirr.pte,adjirr.lci,adjirr.uci))

}

#######################

#' Create blank data set in order to return values using the above function

#######################

dat.cfs_afs_CAP7 <- as.data.frame(matrix(nrow=0,ncol=4))

colnames(dat.cfs_afs_CAP7) <- c("afs","adjirr.pte","adjirr.lci","adjirr.uci")

#' Call above function over the range of the AFS IRR estimated 95% CI bias parameter

###' range: 0.911, 1.070

biasparam_cfs_afs_CAP7 <- seq(from=0.911,to=1.070,by=0.001) # create vector with all values from AFS IRR estimated 95% CI

for(i in 1:length(biasparam_cfs_afs_CAP7)) # create loop to be ran through function created earlier for each value from AFS IRR estimated 95% CI

{

dat.cfs_afs_CAP7[i,] <- biasadj.cfs_afs_CAP7(biasparam_cfs_afs_CAP7[i])

}

#' Return dataset to examine bias adjusted estimates

print(dat.cfs_afs_CAP7)

###############################################################################

#' Graph results

###############################################################################

ggplot(dat.cfs_afs_CAP7, aes(x=afs, y=adjirr.pte)) +

geom_errorbar(aes(y=adjirr.pte, ymin=adjirr.lci, ymax=adjirr.uci), color="grey")+

geom_line()+

geom_point()+

ggtitle("Bias Adjusted Adolescent Firearm Suicide Rate Ratio Assuming Adult

Firearm Suicide Rate Ratio as Negative Control For CAP Law: Owner of Gun is Criminally

Liable if a Gun is Not Stored Properly and a Child Gains Access to the Gun") +

theme(plot.title = element_text(hjust = 0.5))+

xlab("Estimated Adult Firearm Suicide IRR")+

ylab("Bias-Adjusted Adolescent Firearm Suicide IRR")+

geom_hline(yintercept = 1, color = 'darkgrey')+

geom_vline(xintercept = 0.966,color="azure4")+

theme(panel.background = element_blank(),

strip.background = element_rect(colour=NA, fill=NA),

panel.border = element_rect(fill = NA, color = "black")

)

ggsave("AFS_CFS_CAP7.JPEG")

###############################################################################

#' CAP LAW #8: Owner of gun is criminally liable if a gun is not stored properly and the child uses or carries the gun

###############################################################################

###########################################

#' Input Kivisto's point estimate, lower CI, and upper CI

#' estimates as bias parameters for child firearm suicide rates

###########################################

cfs_irr_CAP8.pte <- 0.918 # Point estimate

cfs_irr_CAP8.lci <- 0.768 # Lower CI

cfs_irr_CAP8.uci <- 1.098 # Upper CI

###########################################

#' Create Second Function For Analysis

###########################################

#######################

#' Key for below function:

#' biasadj.cfs_afs_CAP8 = function name

#' adjirr.pte = bias adjusted IRR using the point estimate as bias parameter

#' adjirr.lci = bias adjusted IRR using the lower confidence limit as the bias parameter

#' adjirr.uci = bias adjusted IRR using the upper confidence limit as the bias parameter

#' cfs_irr_CAP8.pte = come from above input as the estimated point estimate bias parameter for child firearm suicide

#' cfs_irr_CAP8.lci = come from above input as the estimated lower confidence limit bias parameter for child firearm suicide

#'cfs_ irr_CAP8.uci = come from above input as the estimated upper confidence limit bias parameter for child firearm suicide

#' afs = afs suicide IRR negative control bias parameter

#' all equations comes from bias formula of estimated IRR of child firearm suicides divided by estimated IRR for adult firearm suicides

#######################

biasadj.cfs_afs_CAP8 <- function(afs)

{

adjirr.pte <- cfs_irr_CAP8.pte/afs

adjirr.lci <- cfs_irr_CAP8.lci/afs

adjirr.uci <- cfs_irr_CAP8.uci/afs

return(c(afs,adjirr.pte,adjirr.lci,adjirr.uci))

}

#######################

#' Create blank data set in order to return values using the above function

#######################

dat.cfs_afs_CAP8 <- as.data.frame(matrix(nrow=0,ncol=4))

colnames(dat.cfs_afs_CAP8) <- c("afs","adjirr.pte","adjirr.lci","adjirr.uci")

#' Call above function over the range of the AFS IRR estimated 95% CI bias parameter

###' range: 0.916, 0.989

biasparam_cfs_afs_CAP8 <- seq(from=0.916,to=0.989,by=0.001) # create vector with all values from AFS IRR estimated 95% CI

for(i in 1:length(biasparam_cfs_afs_CAP8)) # create loop to be ran through function created earlier for each value from AFS IRR estimated 95% CI

{

dat.cfs_afs_CAP8[i,] <- biasadj.cfs_afs_CAP8(biasparam_cfs_afs_CAP8[i])

}

#' Return dataset to examine bias adjusted estimates

print(dat.cfs_afs_CAP8)

###############################################################################

#' Graph results

###############################################################################

ggplot(dat.cfs_afs_CAP8, aes(x=afs, y=adjirr.pte)) +

geom_errorbar(aes(y=adjirr.pte, ymin=adjirr.lci, ymax=adjirr.uci), color="grey")+

geom_line()+

geom_point()+

ggtitle("Bias Adjusted Adolescent Firearm Suicide Rate Ratio Assuming Adult

Firearm Suicide Rate Ratio as Negative Control For CAP Law: Owner of Gun is Criminally

Liable if a Gun is Not Stored Properly and the Child Uses or Carries the Gun") +

theme(plot.title = element_text(hjust = 0.5))+

xlab("Estimated Adult Firearm Suicide IRR")+

ylab("Bias-Adjusted Adolescent Firearm Suicide IRR")+

geom_hline(yintercept = 1, color = 'darkgrey')+

geom_vline(xintercept = 0.952,color="azure4")+

theme(panel.background = element_blank(),

strip.background = element_rect(colour=NA, fill=NA),

panel.border = element_rect(fill = NA, color = "black")

)

ggsave("AFS_CFS_CAP8.JPEG")

###############################################################################

#' CAP LAW #9: Owner of gun is criminally liable regardless of whether gun is loaded or unloaded

###############################################################################

###########################################

#' Input Kivisto's point estimate, lower CI, and upper CI

#' estimates as bias parameters for child firearm suicide rates

###########################################

cfs_irr_CAP9.pte <- 0.515 # Point estimate

cfs_irr_CAP9.lci <- 0.316 # Lower CI

cfs_irr_CAP9.uci <- 0.839 # Upper CI

###########################################

#' Create Second Function For Analysis

###########################################

#######################

#' Key for below function:

#' biasadj.cfs_afs_CAP9 = function name

#' adjirr.pte = bias adjusted IRR using the point estimate as bias parameter

#' adjirr.lci = bias adjusted IRR using the lower confidence limit as the bias parameter

#' adjirr.uci = bias adjusted IRR using the upper confidence limit as the bias parameter

#' cfs_irr_CAP9.pte = come from above input as the estimated point estimate bias parameter for child firearm suicide

#' cfs_irr_CAP9.lci = come from above input as the estimated lower confidence limit bias parameter for child firearm suicide

#'cfs_ irr_CAP9.uci = come from above input as the estimated upper confidence limit bias parameter for child firearm suicide

#' afs = afs suicide IRR negative control bias parameter

#' all equations comes from bias formula of estimated IRR of child firearm suicides divided by estimated IRR for adult firearm suicides

#######################

biasadj.cfs_afs_CAP9 <- function(afs)

{

adjirr.pte <- cfs_irr_CAP9.pte/afs

adjirr.lci <- cfs_irr_CAP9.lci/afs

adjirr.uci <- cfs_irr_CAP9.uci/afs

return(c(afs,adjirr.pte,adjirr.lci,adjirr.uci))

}

#######################

#' Create blank data set in order to return values using the above function

#######################

dat.cfs_afs_CAP9 <- as.data.frame(matrix(nrow=0,ncol=4))

colnames(dat.cfs_afs_CAP9) <- c("afs","adjirr.pte","adjirr.lci","adjirr.uci")

#' Call above function over the range of the AFS IRR estimated 95% CI bias parameter

###' range: 0.863, 1.068

biasparam_cfs_afs_CAP9 <- seq(from=0.863,to=1.068,by=0.001) # create vector with all values from AFS IRR estimated 95% CI

for(i in 1:length(biasparam_cfs_afs_CAP9)) # create loop to be ran through function created earlier for each value from AFS IRR estimated 95% CI

{

dat.cfs_afs_CAP9[i,] <- biasadj.cfs_afs_CAP9(biasparam_cfs_afs_CAP9[i])

}

#' Return dataset to examine bias adjusted estimates

print(dat.cfs_afs_CAP9)

###############################################################################

#' Graph results

###############################################################################

ggplot(dat.cfs_afs_CAP9, aes(x=afs, y=adjirr.pte)) +

geom_errorbar(aes(y=adjirr.pte, ymin=adjirr.lci, ymax=adjirr.uci), color="grey")+

geom_line()+

geom_point()+

ggtitle("Bias Adjusted Adolescent Firearm Suicide Rate Ratio Assuming Adult

Firearm Suicide Rate Ratio as Negative Control For CAP Law: Owner of Gun is Criminally

Liable Regardless of Whether Gun is Loaded or Unloaded") +

theme(plot.title = element_text(hjust = 0.5))+

xlab("Estimated Adult Firearm Suicide IRR")+

ylab("Bias-Adjusted Adolescent Firearm Suicide IRR")+

geom_hline(yintercept = 1, color = 'darkgrey')+

geom_vline(xintercept = 0.960,color="azure4")+

theme(panel.background = element_blank(),

strip.background = element_rect(colour=NA, fill=NA),

panel.border = element_rect(fill = NA, color = "black")

)

ggsave("AFS_CFS_CAP9.JPEG")

###############################################################################

#' CAP LAW #10: Criminal liability based on the age of the child

###############################################################################

###########################################

#' Input Kivisto's point estimate, lower CI, and upper CI

#' estimates as bias parameters for child firearm suicide rates

###########################################

cfs_irr_CAP10.pte <- 0.943 # Point estimate

cfs_irr_CAP10.lci <- 0.866 # Lower CI

cfs_irr_CAP10.uci <- 1.025 # Upper CI

###########################################

#' Create Second Function For Analysis

###########################################

#######################

#' Key for below function:

#' biasadj.cfs_afs_CAP10 = function name

#' adjirr.pte = bias adjusted IRR using the point estimate as bias parameter

#' adjirr.lci = bias adjusted IRR using the lower confidence limit as the bias parameter

#' adjirr.uci = bias adjusted IRR using the upper confidence limit as the bias parameter

#' cfs_irr_CAP10.pte = come from above input as the estimated point estimate bias parameter for child firearm suicide

#' cfs_irr_CAP10.lci = come from above input as the estimated lower confidence limit bias parameter for child firearm suicide

#'cfs_ irr_CAP10.uci = come from above input as the estimated upper confidence limit bias parameter for child firearm suicide

#' afs = afs suicide IRR negative control bias parameter

#' all equations comes from bias formula of estimated IRR of child firearm suicides divided by estimated IRR for adult firearm suicides

#######################

biasadj.cfs_afs_CAP10 <- function(afs)

{

adjirr.pte <- cfs_irr_CAP10.pte/afs

adjirr.lci <- cfs_irr_CAP10.lci/afs

adjirr.uci <- cfs_irr_CAP10.uci/afs

return(c(afs,adjirr.pte,adjirr.lci,adjirr.uci))

}

#######################

#' Create blank data set in order to return values using the above function

#######################

dat.cfs_afs_CAP10 <- as.data.frame(matrix(nrow=0,ncol=4))

colnames(dat.cfs_afs_CAP10) <- c("afs","adjirr.pte","adjirr.lci","adjirr.uci")

#' Call above function over the range of the AFS IRR estimated 95% CI bias parameter

###' range: 0.959, 0.993

biasparam_cfs_afs_CAP10 <- seq(from=0.959,to=0.993,by=0.001) # create vector with all values from AFS IRR estimated 95% CI

for(i in 1:length(biasparam_cfs_afs_CAP10)) # create loop to be ran through function created earlier for each value from AFS IRR estimated 95% CI

{

dat.cfs_afs_CAP10[i,] <- biasadj.cfs_afs_CAP10(biasparam_cfs_afs_CAP10[i])

}

#' Return dataset to examine bias adjusted estimates

print(dat.cfs_afs_CAP10)

###############################################################################

#' Graph results

###############################################################################

ggplot(dat.cfs_afs_CAP10, aes(x=afs, y=adjirr.pte)) +

geom_errorbar(aes(y=adjirr.pte, ymin=adjirr.lci, ymax=adjirr.uci), color="grey")+

geom_line()+

geom_point()+

ggtitle("Bias Adjusted Adolescent Firearm Suicide Rate Ratio Assuming Adult

Firearm Suicide Rate Ratio as Negative Control For CAP Law: Criminal Liability

Based on the Age of the Child") +

theme(plot.title = element_text(hjust = 0.5))+

xlab("Estimated Adult Firearm Suicide IRR")+

ylab("Bias-Adjusted Adolescent Firearm Suicide IRR")+

geom_hline(yintercept = 1, color = 'darkgrey')+

geom_vline(xintercept = 0.976,color="azure4")+

theme(panel.background = element_blank(),

strip.background = element_rect(colour=NA, fill=NA),

panel.border = element_rect(fill = NA, color = "black")

)

ggsave("AFS_CFS_CAP10.JPEG")

###############################################################################

#' CAP LAW #11: Owner of gun is criminally liable if child under age 14 gains access to the gun

###############################################################################

###########################################

#' Input Kivisto's point estimate, lower CI, and upper CI

#' estimates as bias parameters for child firearm suicide rates

###########################################

cfs_irr_CAP11.pte <- 0.918 # Point estimate

cfs_irr_CAP11.lci <- 0.768 # Lower CI

cfs_irr_CAP11.uci <- 1.098 # Upper CI

###########################################

#' Create Second Function For Analysis

###########################################

#######################

#' Key for below function:

#' biasadj.cfs_afs_CAP11 = function name

#' adjirr.pte = bias adjusted IRR using the point estimate as bias parameter

#' adjirr.lci = bias adjusted IRR using the lower confidence limit as the bias parameter

#' adjirr.uci = bias adjusted IRR using the upper confidence limit as the bias parameter

#' cfs_irr_CAP11.pte = come from above input as the estimated point estimate bias parameter for child firearm suicide

#' cfs_irr_CAP11.lci = come from above input as the estimated lower confidence limit bias parameter for child firearm suicide

#'cfs_ irr_CAP11.uci = come from above input as the estimated upper confidence limit bias parameter for child firearm suicide

#' afs = afs suicide IRR negative control bias parameter

#' all equations comes from bias formula of estimated IRR of child firearm suicides divided by estimated IRR for adult firearm suicides

#######################

biasadj.cfs_afs_CAP11 <- function(afs)

{

adjirr.pte <- cfs_irr_CAP11.pte/afs

adjirr.lci <- cfs_irr_CAP11.lci/afs

adjirr.uci <- cfs_irr_CAP11.uci/afs

return(c(afs,adjirr.pte,adjirr.lci,adjirr.uci))

}

#######################

#' Create blank data set in order to return values using the above function

#######################

dat.cfs_afs_CAP11 <- as.data.frame(matrix(nrow=0,ncol=4))

colnames(dat.cfs_afs_CAP11) <- c("afs","adjirr.pte","adjirr.lci","adjirr.uci")

#' Call above function over the range of the AFS IRR estimated 95% CI bias parameter

###' range: 0.865, 0.982

biasparam_cfs_afs_CAP11 <- seq(from=0.865,to=0.982,by=0.001) # create vector with all values from AFS IRR estimated 95% CI

for(i in 1:length(biasparam_cfs_afs_CAP11)) # create loop to be ran through function created earlier for each value from AFS IRR estimated 95% CI

{

dat.cfs_afs_CAP11[i,] <- biasadj.cfs_afs_CAP11(biasparam_cfs_afs_CAP11[i])

}

#' Return dataset to examine bias adjusted estimates

print(dat.cfs_afs_CAP11)

###############################################################################

#' Graph results

###############################################################################

ggplot(dat.cfs_afs_CAP11, aes(x=afs, y=adjirr.pte)) +

geom_errorbar(aes(y=adjirr.pte, ymin=adjirr.lci, ymax=adjirr.uci), color="grey")+

geom_line()+

geom_point()+

ggtitle("Bias Adjusted Adolescent Firearm Suicide Rate Ratio Assuming Adult

Firearm Suicide Rate Ratio as Negative Control For CAP Law: Owner of Gun is Criminally

Liable if Child Under Age 14 Gains Access to the Gun") +

theme(plot.title = element_text(hjust = 0.5))+

xlab("Estimated Adult Firearm Suicide IRR")+

ylab("Bias-Adjusted Adolescent Firearm Suicide IRR")+

geom_hline(yintercept = 1, color = 'darkgrey')+

geom_vline(xintercept = 0.9219,color="azure4")+

theme(panel.background = element_blank(),

strip.background = element_rect(colour=NA, fill=NA),

panel.border = element_rect(fill = NA, color = "black")

)

ggsave("AFS_CFS_CAP11.JPEG")

###############################################################################

#' CAP LAW #12: Owner of gun is criminally liable if child under age 16 gains access to the gun

###############################################################################

###########################################

#' Input Kivisto's point estimate, lower CI, and upper CI

#' estimates as bias parameters for child firearm suicide rates

###########################################

cfs_irr_CAP12.pte <- 0.853 # Point estimate

cfs_irr_CAP12.lci <- 0.690 # Lower CI

cfs_irr_CAP12.uci <- 1.053 # Upper CI

###########################################

#' Create Second Function For Analysis

###########################################

#######################

#' Key for below function:

#' biasadj.cfs_afs_CAP12 = function name

#' adjirr.pte = bias adjusted IRR using the point estimate as bias parameter

#' adjirr.lci = bias adjusted IRR using the lower confidence limit as the bias parameter

#' adjirr.uci = bias adjusted IRR using the upper confidence limit as the bias parameter

#' cfs_irr_CAP12.pte = come from above input as the estimated point estimate bias parameter for child firearm suicide

#' cfs_irr_CAP12.lci = come from above input as the estimated lower confidence limit bias parameter for child firearm suicide

#'cfs_ irr_CAP12.uci = come from above input as the estimated upper confidence limit bias parameter for child firearm suicide

#' afs = afs suicide IRR negative control bias parameter

#' all equations comes from bias formula of estimated IRR of child firearm suicides divided by estimated IRR for adult firearm suicides

#######################

biasadj.cfs_afs_CAP12 <- function(afs)

{

adjirr.pte <- cfs_irr_CAP12.pte/afs

adjirr.lci <- cfs_irr_CAP12.lci/afs

adjirr.uci <- cfs_irr_CAP12.uci/afs

return(c(afs,adjirr.pte,adjirr.lci,adjirr.uci))

}

#######################

#' Create blank data set in order to return values using the above function

#######################

dat.cfs_afs_CAP12 <- as.data.frame(matrix(nrow=0,ncol=4))

colnames(dat.cfs_afs_CAP12) <- c("afs","adjirr.pte","adjirr.lci","adjirr.uci")

#' Call above function over the range of the AFS IRR estimated 95% CI bias parameter

###' range: 0.909, 0.994

biasparam_cfs_afs_CAP12 <- seq(from=0.909,to=0.994,by=0.001) # create vector with all values from AFS IRR estimated 95% CI

for(i in 1:length(biasparam_cfs_afs_CAP12)) # create loop to be ran through function created earlier for each value from AFS IRR estimated 95% CI

{

dat.cfs_afs_CAP12[i,] <- biasadj.cfs_afs_CAP12(biasparam_cfs_afs_CAP12[i])

}

#' Return dataset to examine bias adjusted estimates

print(dat.cfs_afs_CAP12)

###############################################################################

#' Graph results

###############################################################################

ggplot(dat.cfs_afs_CAP12, aes(x=afs, y=adjirr.pte)) +

geom_errorbar(aes(y=adjirr.pte, ymin=adjirr.lci, ymax=adjirr.uci), color="grey")+

geom_line()+

geom_point()+

ggtitle("Bias Adjusted Adolescent Firearm Suicide Rate Ratio Assuming Adult

Firearm Suicide Rate Ratio as Negative Control For CAP Law: Owner of Gun is Criminally

Liable if Child Under Age 16 Gains Access to the Gun") +

theme(plot.title = element_text(hjust = 0.5))+

xlab("Estimated Adult Firearm Suicide IRR")+

ylab("Bias-Adjusted Adolescent Firearm Suicide IRR")+

geom_hline(yintercept = 1, color = 'darkgrey')+

geom_vline(xintercept = 0.951,color="azure4")+

theme(panel.background = element_blank(),

strip.background = element_rect(colour=NA, fill=NA),

panel.border = element_rect(fill = NA, color = "black")

)

ggsave("AFS_CFS_CAP12.JPEG")

###############################################################################

#' CAP LAW #13: Owner of gun is criminally liable if child under age 18 gains access to the gun

###############################################################################

###########################################

#' Input Kivisto's point estimate, lower CI, and upper CI

#' estimates as bias parameters for child firearm suicide rates

###########################################

cfs_irr_CAP13.pte <- 0.824 # Point estimate

cfs_irr_CAP13.lci <- 0.605 # Lower CI

cfs_irr_CAP13.uci <- 1.121 # Upper CI

###########################################

#' Create Second Function For Analysis

###########################################

#######################

#' Key for below function:

#' biasadj.cfs_afs_CAP13 = function name

#' adjirr.pte = bias adjusted IRR using the point estimate as bias parameter

#' adjirr.lci = bias adjusted IRR using the lower confidence limit as the bias parameter

#' adjirr.uci = bias adjusted IRR using the upper confidence limit as the bias parameter

#' cfs_irr_CAP13.pte = come from above input as the estimated point estimate bias parameter for child firearm suicide

#' cfs_irr_CAP13.lci = come from above input as the estimated lower confidence limit bias parameter for child firearm suicide

#'cfs_ irr_CAP13.uci = come from above input as the estimated upper confidence limit bias parameter for child firearm suicide

#' afs = afs suicide IRR negative control bias parameter

#' all equations comes from bias formula of estimated IRR of child firearm suicides divided by estimated IRR for adult firearm suicides

#######################

biasadj.cfs_afs_CAP13 <- function(afs)

{

adjirr.pte <- cfs_irr_CAP13.pte/afs

adjirr.lci <- cfs_irr_CAP13.lci/afs

adjirr.uci <- cfs_irr_CAP13.uci/afs

return(c(afs,adjirr.pte,adjirr.lci,adjirr.uci))

}

#######################

#' Create blank data set in order to return values using the above function

#######################

dat.cfs_afs_CAP13 <- as.data.frame(matrix(nrow=0,ncol=4))

colnames(dat.cfs_afs_CAP13) <- c("afs","adjirr.pte","adjirr.lci","adjirr.uci")

#' Call above function over the range of the AFS IRR estimated 95% CI bias parameter

###' range: 0.916, 0.989

biasparam_cfs_afs_CAP13 <- seq(from=0.916,to=0.989,by=0.001) # create vector with all values from AFS IRR estimated 95% CI

for(i in 1:length(biasparam_cfs_afs_CAP13)) # create loop to be ran through function created earlier for each value from AFS IRR estimated 95% CI

{

dat.cfs_afs_CAP13[i,] <- biasadj.cfs_afs_CAP13(biasparam_cfs_afs_CAP13[i])

}

#' Return dataset to examine bias adjusted estimates

print(dat.cfs_afs_CAP13)

###############################################################################

#' Graph results

###############################################################################

ggplot(dat.cfs_afs_CAP13, aes(x=afs, y=adjirr.pte)) +

geom_errorbar(aes(y=adjirr.pte, ymin=adjirr.lci, ymax=adjirr.uci), color="grey")+

geom_line()+

geom_point()+

ggtitle("Bias Adjusted Adolescent Firearm Suicide Rate Ratio Assuming Adult

Firearm Suicide Rate Ratio as Negative Control For CAP Law: Owner of Gun is Criminally

Liable if Child Under Age 18 Gains Access to the Gun") +

theme(plot.title = element_text(hjust = 0.5))+

xlab("Estimated Adult Firearm Suicide IRR")+

ylab("Bias-Adjusted Adolescent Firearm Suicide IRR")+

geom_hline(yintercept = 1, color = 'darkgrey')+

geom_vline(xintercept = 0.952,color="azure4")+

theme(panel.background = element_blank(),

strip.background = element_rect(colour=NA, fill=NA),

panel.border = element_rect(fill = NA, color = "black")

)

ggsave("AFS_CFS_CAP13.JPEG")
