# Supplementary material for: The effect of child access prevention laws on adolescent suicide: a negative control approach
Source: Inj Epidemiol. 2025 Apr 23;12:21. doi: 10.1186/s40621-025-00577-x (PMC12020014; doi:10.1186/s40621-025-00577-x)
Supplement: Supplementary file 2 — Supplementary Material 2 [file 40621_2025_577_MOESM2_ESM.docx]

**Supplementary Material 2**

**Supplement 1**

Formula for risk ratios in the presence of a binary unmeasured confounder:

$$\frac{{RR}_{k}^{est}}{{RR}_{k}^{true}}= \frac{1+\left( \gamma_{k}-1 \right)\Pr\left[ U=1 \right|A=1, L=l]}{1+\left( \gamma_{k}-1 \right)\Pr\left[ U=1 \right|A=0, L=l]}$$

such that *k* indexes that this formula is specific to a particular outcome Y_k_, *A* denotes CAP law status, *L* denotes the measured variables adjusted for in the current study, *U* denotes a binary unmeasured confounder, and 𝛾_k_ is defined as follows:

$$\gamma_{k}= \frac{E\left[ Y_{k} \right|A=a, L=l, U=1]}{E\left[ Y_{k} \right|A=a, L=l, U=0]}$$

and is assumed to be the same for levels of baseline CAP law status. In the conjecture below, we consider scenarios for, adolescent firearm suicide and adolescent non-firearm suicide which we index with *k* = *cfs*, *cnfs*, respectively as well as adult firearm suicide and adolescent firearm suicide which we index with *k* = a*fs*, *cfs*, respectively.

**Conjecture**

Consider an arbitrary binary unmeasured confounder such that the conditional relative risk

of the confounder with child non-firearm (cnfs) and child firearm (cfs), as well as adult (afs) and child firearm suicide (cfs) is constant (i.e., 𝛾_cf𝑠_ = 𝛾_cn𝑓𝑠_ and 𝛾_afs =_ 𝛾_cfs_). If the true effect of CAP laws on non-firearm suicide risk is null (i.e., ${RR}_{cnfs}^{true}=1$), and failing to adjust for this confounder fully explains the non-null estimates, then the effect of CAP laws on adolescent firearm suicide risk would be $\frac{{RR}_{cfs}^{est}}{{RR}_{cnfs}^{est}}$. If the true effect of CAP laws on adult firearm suicide is null (i.e., ${RR}_{afs}^{true}=1$), and failing to adjust for this confounder fully explains the non-null estimates, the effect of CAP laws on adolescent firearm suicide risk would be $\frac{{RR}_{cfs}^{est}}{{RR}_{afs}^{est}}$.

**Proof**

Assuming 𝛾_cf𝑠_ = 𝛾_cn𝑓𝑠_ and 𝛾_afs =_ 𝛾_cfs_, trivially implies that the following ratios are equal to one another and equal to the estimated relative risk for non-firearm suicide when ${RR}_{cnfs}^{true}=1$ as well as the estimated relative risk for adult firearm suicide when ${RR}_{afs}^{true}=1$:

$\frac{{RR}_{cfs}^{est}}{{RR}_{cfs}^{true}}=\frac{{RR}_{cnfs}^{est}}{{RR}_{cnfs}^{true}}= {RR}_{cnfs}^{est}$ and $\frac{{RR}_{afs}^{est}}{{RR}_{afs}^{true}}=\frac{{RR}_{cfs}^{est}}{{RR}_{cfs}^{true}}= {RR}_{afs}^{est}$

then,

$\frac{{RR}_{cfs}^{est}}{{RR}_{cfs}^{true}}= {RR}_{cnfs}^{est}$ and $\frac{{RR}_{cfs}^{est}}{{RR}_{cfs}^{true}}= {RR}_{afs}^{est}$

Multiple by ${RR}_{cfs}^{true}$ and ${RR}_{cfs}^{true}$

$${RR}_{cfs}^{true}\left( \frac{{RR}_{cfs}^{est}}{{RR}_{cfs}^{true}}= {RR}_{cnfs}^{est} \right) \mathrm{and}{RR}_{cfs}^{true}\left( \frac{{RR}_{cfs}^{est}}{{RR}_{cfs}^{true}}= {RR}_{afs}^{est} \right)$$

${RR}_{cfs}^{true} \mathrm{and} {RR}_{cfs}^{true}$ cancels

$${RR}_{cfs}^{est}={RR}_{cnfs}^{est} \times{RR}_{cfs}^{true} \mathrm{and}{RR}_{cfs}^{est}={RR}_{afs}^{est} \times{RR}_{cfs}^{true}$$

Divide by ${RR}_{cnfs}^{est}$ and ${RR}_{afs}^{est}$

$$\frac{{RR}_{cfs}^{est}={RR}_{cnfs}^{est} \times{RR}_{cfs}^{true}}{{RR}_{cnfs}^{est}} \mathrm{and}\frac{{RR}_{cfs}^{est}={RR}_{afs}^{est} \times{RR}_{cfs}^{true}}{{RR}_{afs}^{est}}$$

Thus,

$${RR}_{cfs}^{true}=\frac{{RR}_{cfs}^{est}}{{RR}_{cnfs}^{est}} \mathrm{and}{RR}_{cfs}^{true}=\frac{{RR}_{cfs}^{est}}{{RR}_{afs}^{est}}$$

**Supplement 2-Supplemental bias analyses for specific versions of CAP laws using adolescent non-firearm suicide as the negative control:**

**
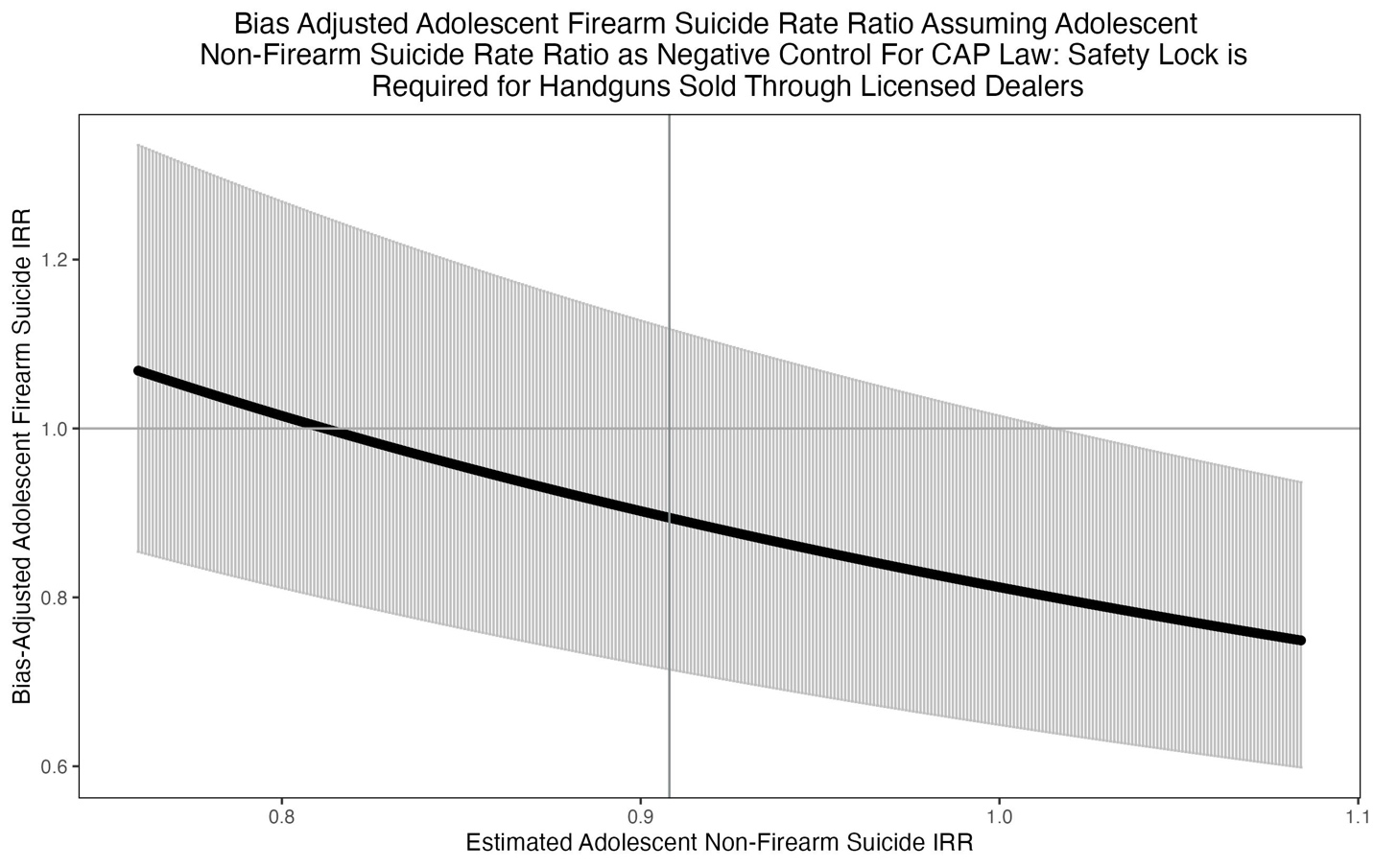
Figure S1**-Safety Lock is Required for Handguns Sold Through Licensed Dealers:

Black dots represent the bias-adjusted point estimates. Error bars represent the bias-adjusted 95% confidence intervals. The vertical line originating at 0.908 on the x-axis represents the Kivisto and colleagues estimated adolescent non-firearm suicide IRR. The horizontal line originating from the y-axis represents the null value. IRR=Incident Rate Ratio

**
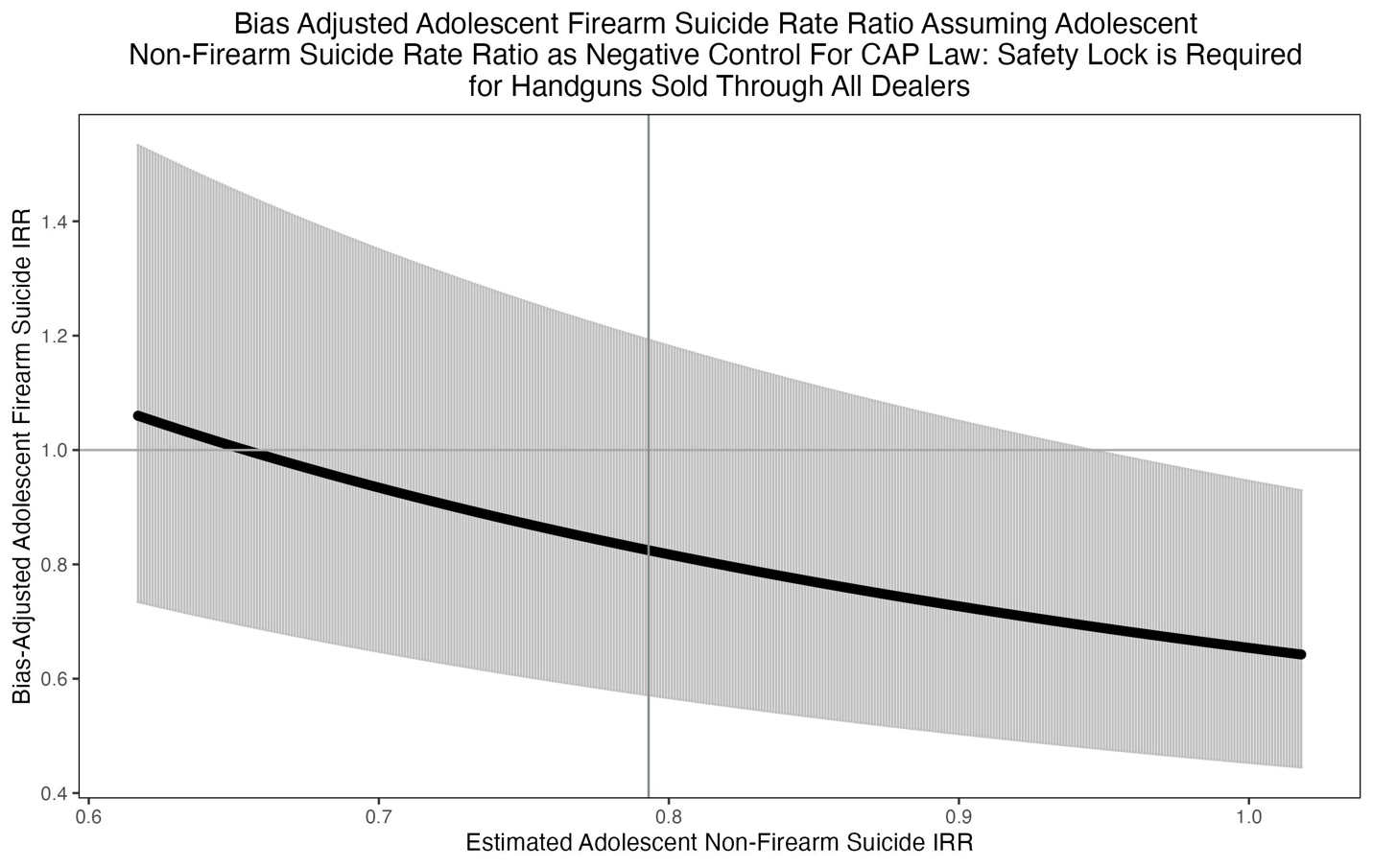
Figure S2**- Safety Lock is Required for Handguns Sold Through All Dealers:

Black dots represent the bias-adjusted point estimates. Error bars represent the bias-adjusted 95% confidence intervals. The vertical line originating at 0.793 on the x-axis represents the Kivisto and colleagues estimated adolescent non-firearm suicide IRR. The horizontal line originating from the y-axis represents the null value. IRR=Incident Rate Ratio.

**
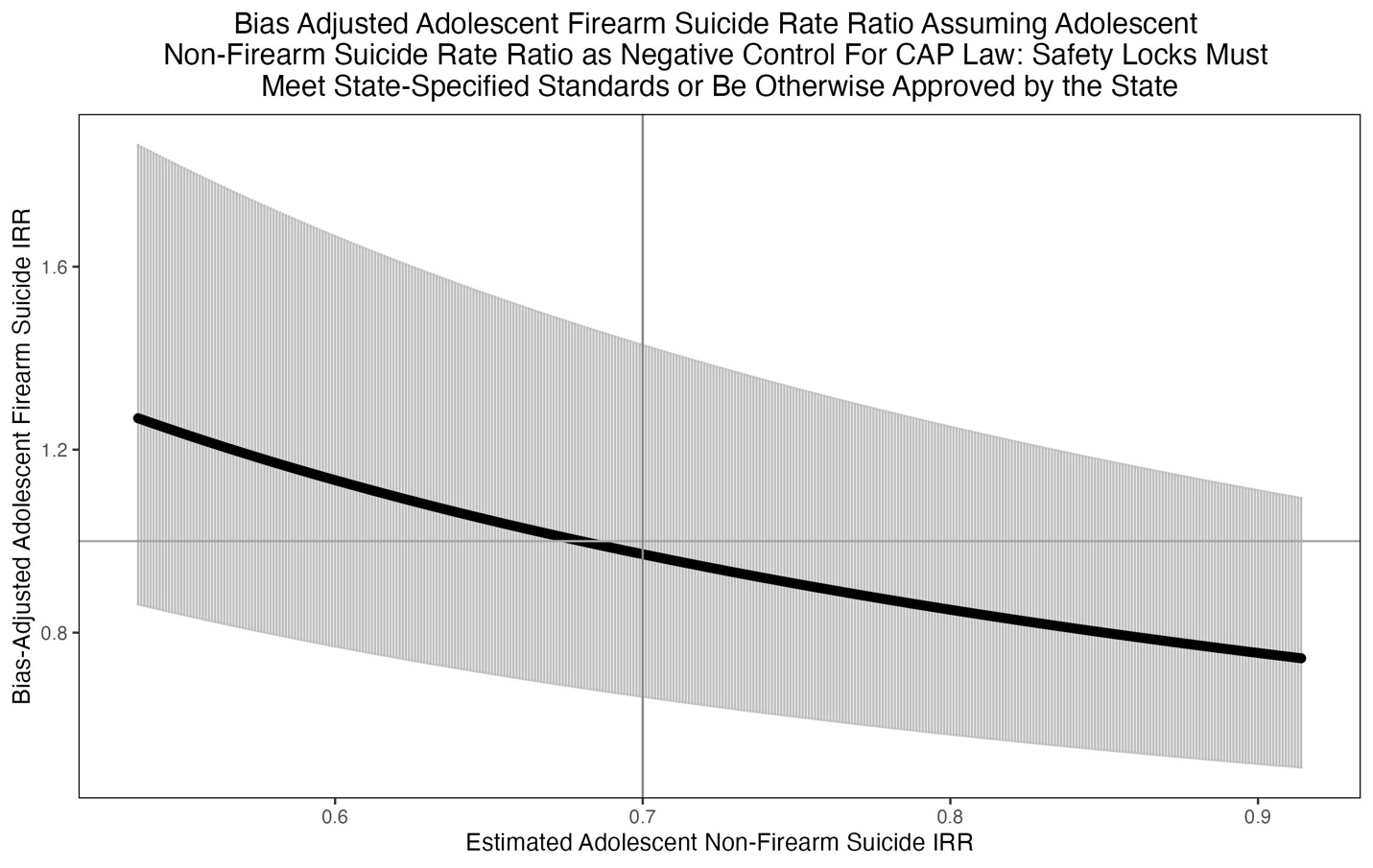
Figure S3**-Safety Locks Must Meet State-Specified Standards or be Otherwise Approved by the State:

Black dots represent the bias-adjusted point estimates. Error bars represent the bias-adjusted 95% confidence intervals. The vertical line originating at 0.700 on the x-axis represents the Kivisto and colleagues estimated adolescent non-firearm suicide IRR. The horizontal line originating from the y-axis represents the null value. IRR=Incident Rate Ratio.

**
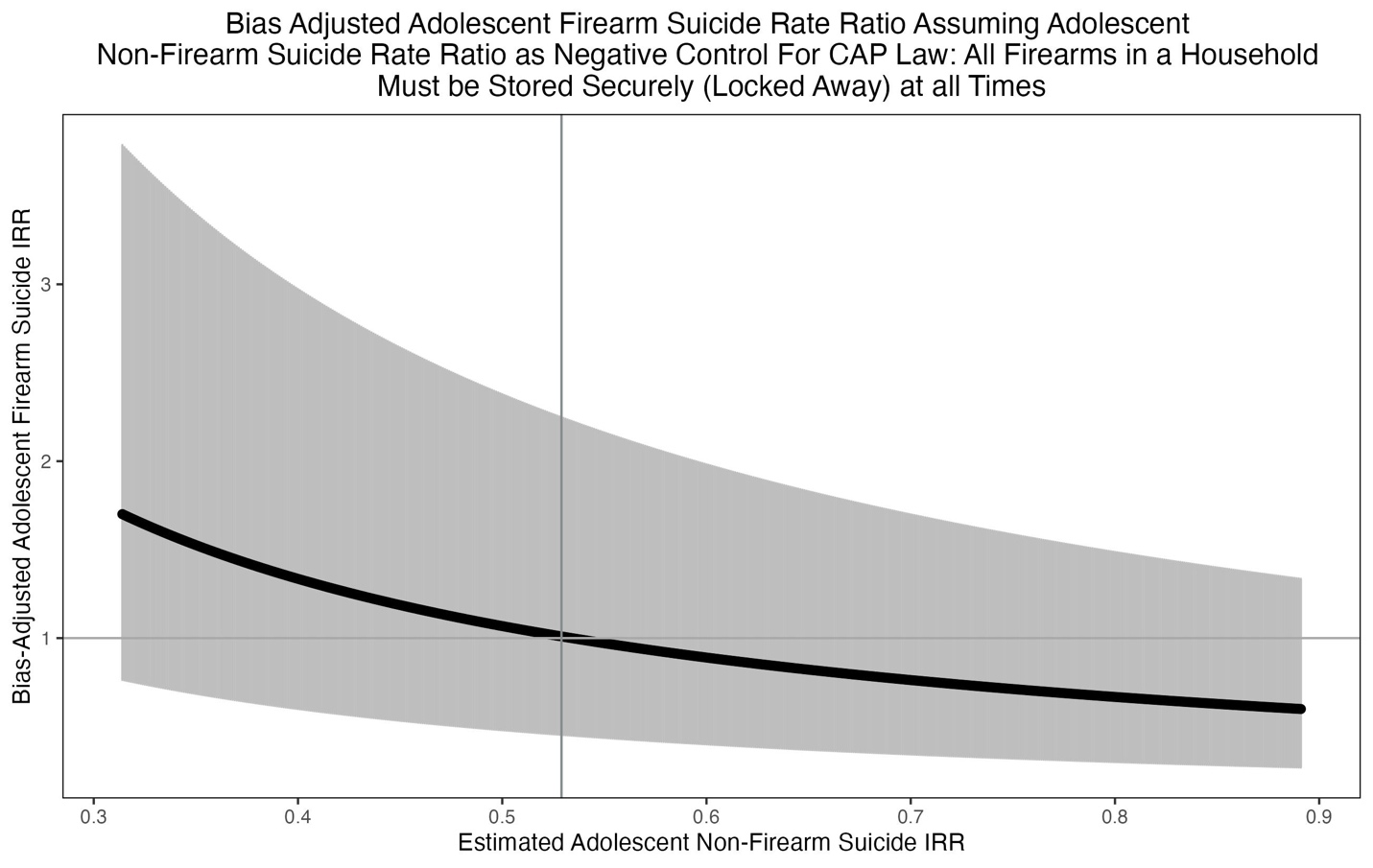
Figure S4**-All Firearms in a Household Must be Stored Securely (Locked Away) at All Times:

Black dots represent the bias-adjusted point estimates. Error bars represent the bias-adjusted 95% confidence intervals. The vertical line originating at 0.529 on the x-axis represents the Kivisto and colleagues estimated adolescent non-firearm suicide IRR. The horizontal line originating from the y-axis represents the null value. IRR=Incident Rate Ratio.

**
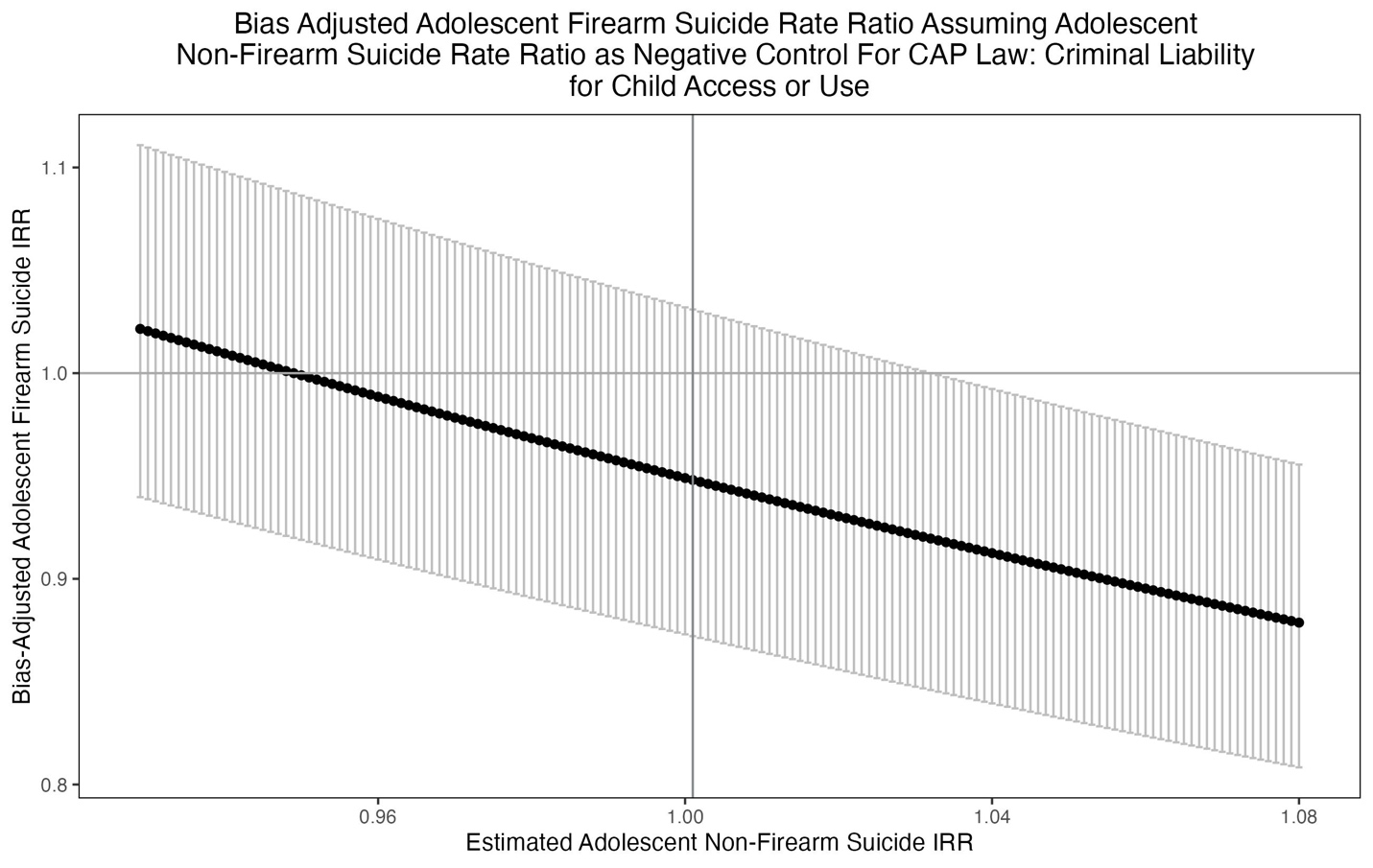
Figure S5**-Criminal Liability for Child Access or Use:

Black dots represent the bias-adjusted point estimates. Error bars represent the bias-adjusted 95% confidence intervals. The vertical line originating at 1.001 on the x-axis represents the Kivisto and colleagues estimated adolescent non-firearm suicide IRR. The horizontal line originating from the y-axis represents the null value. IRR=Incident Rate Ratio.

**
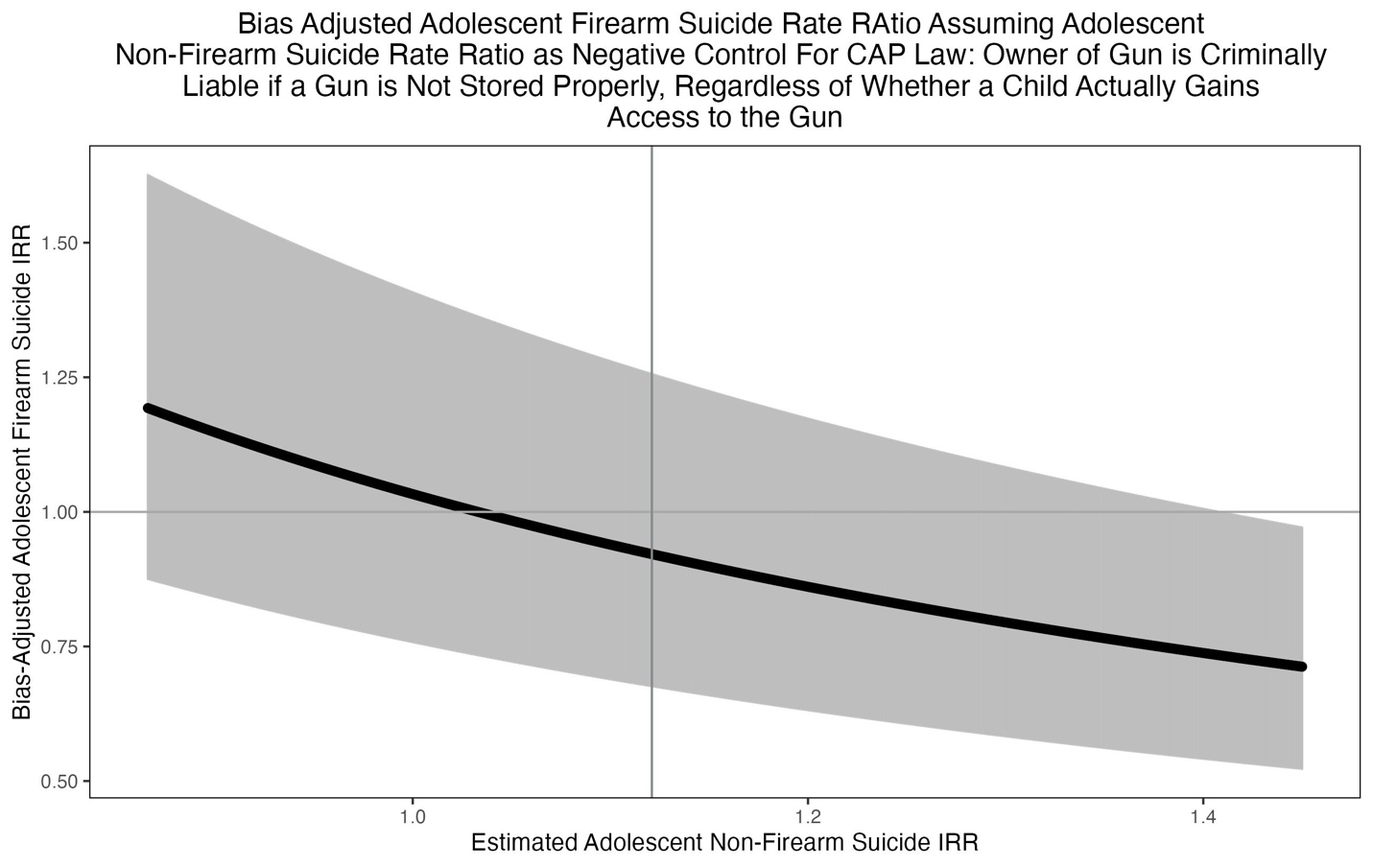
Figure S6**-Owner of Gun is Criminally Liable if a Gun is Not Stored Properly, Regardless of Whether a Child Actually Gains Access to the Gun:

Black dots represent the bias-adjusted point estimates. Error bars represent the bias-adjusted 95% confidence intervals. The vertical line originating at 1.121 on the x-axis represents the Kivisto and colleagues estimated adolescent non-firearm suicide IRR. The horizontal line originating from the y-axis represents the null value. IRR=Incident Rate Ratio.

**
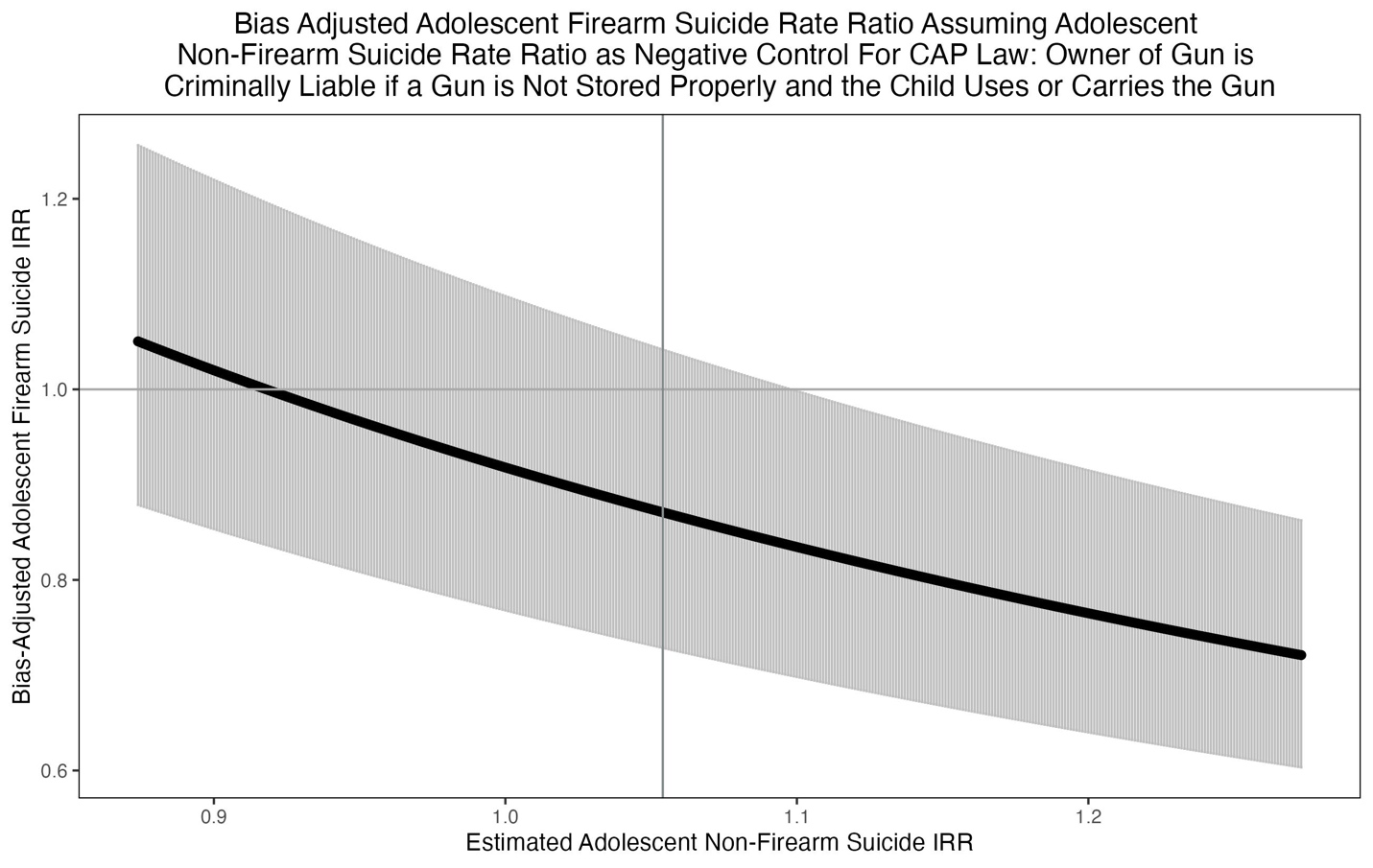
Figure S7**-Owner of Gun is Criminally Liable if a Gun is Not Stored Properly and the Child Uses or Carries the Gun:

Black dots represent the bias-adjusted point estimates. Error bars represent the bias-adjusted 95% confidence intervals. The vertical line originating at 1.054 on the x-axis represents the Kivisto and colleagues estimated adolescent non-firearm suicide IRR. The horizontal line originating from the y-axis represents the null value. IRR=Incident Rate Ratio.


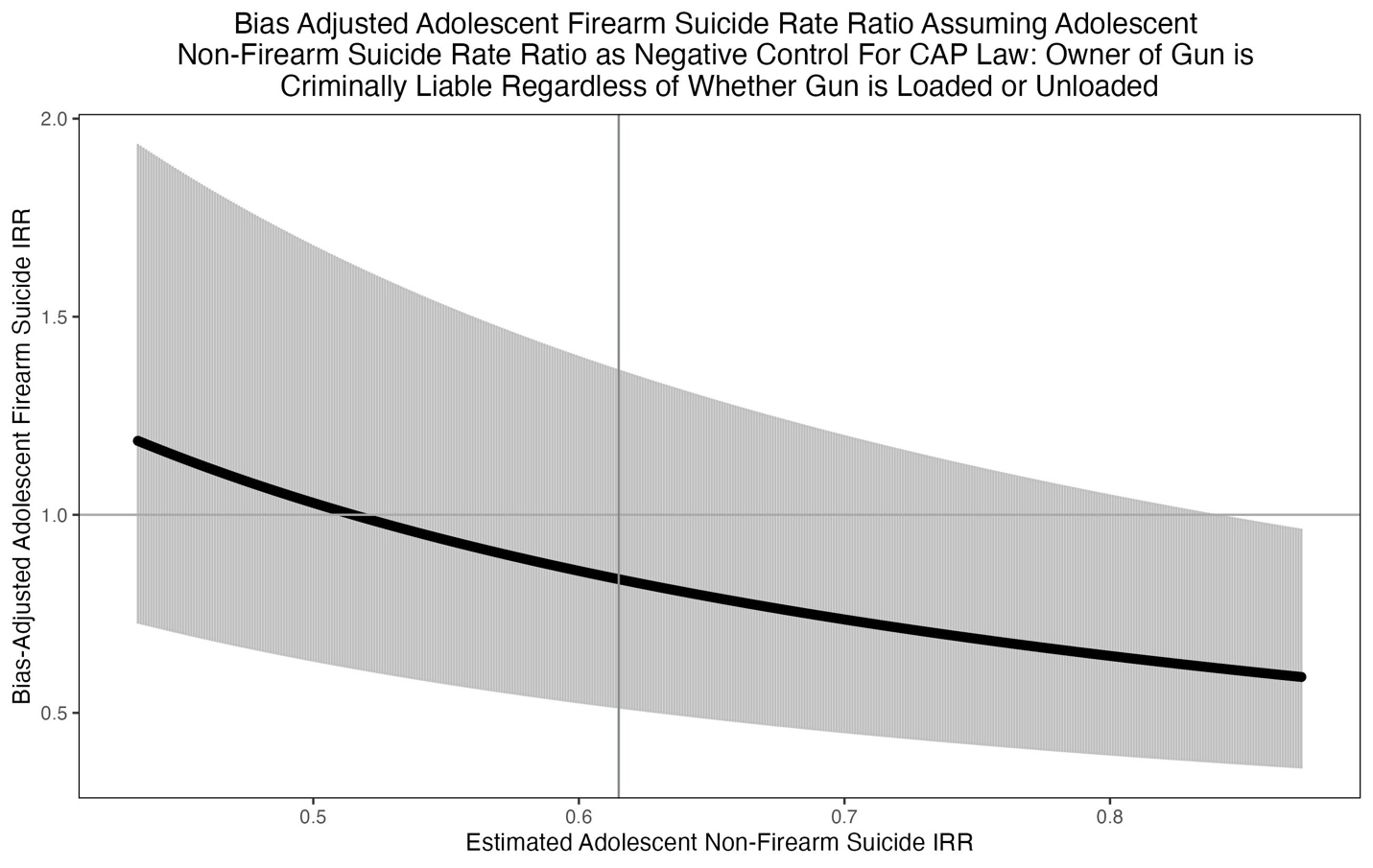
**Figure S8**-Owner of Gun is Criminally Liable Regardless of Whether Gun is Loaded or Unloaded:

Black dots represent the bias-adjusted point estimates. Error bars represent the bias-adjusted 95% confidence intervals. The vertical line originating at 0.615 on the x-axis represents the Kivisto and colleagues estimated adolescent non-firearm suicide IRR. The horizontal line originating from the y-axis represents the null value. IRR=Incident Rate Ratio.

**
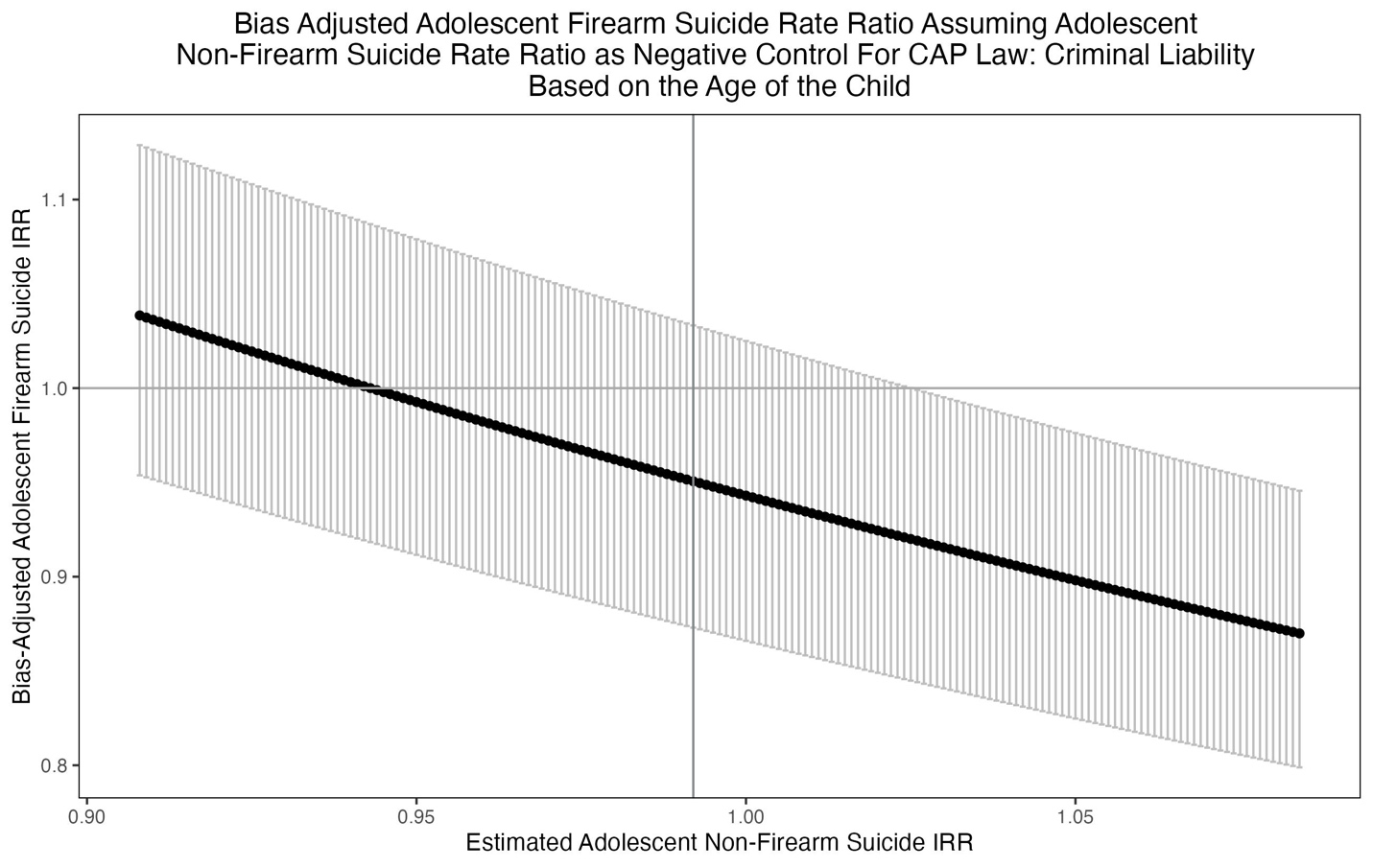
Figure S9**-Criminal Liability Based on the Age of the Child:

Black dots represent the bias-adjusted point estimates. Error bars represent the bias-adjusted 95% confidence intervals. The vertical line originating at 0.992 on the x-axis represents the Kivisto and colleagues estimated adolescent non-firearm suicide IRR. The horizontal line originating from the y-axis represents the null value. IRR=Incident Rate Ratio.


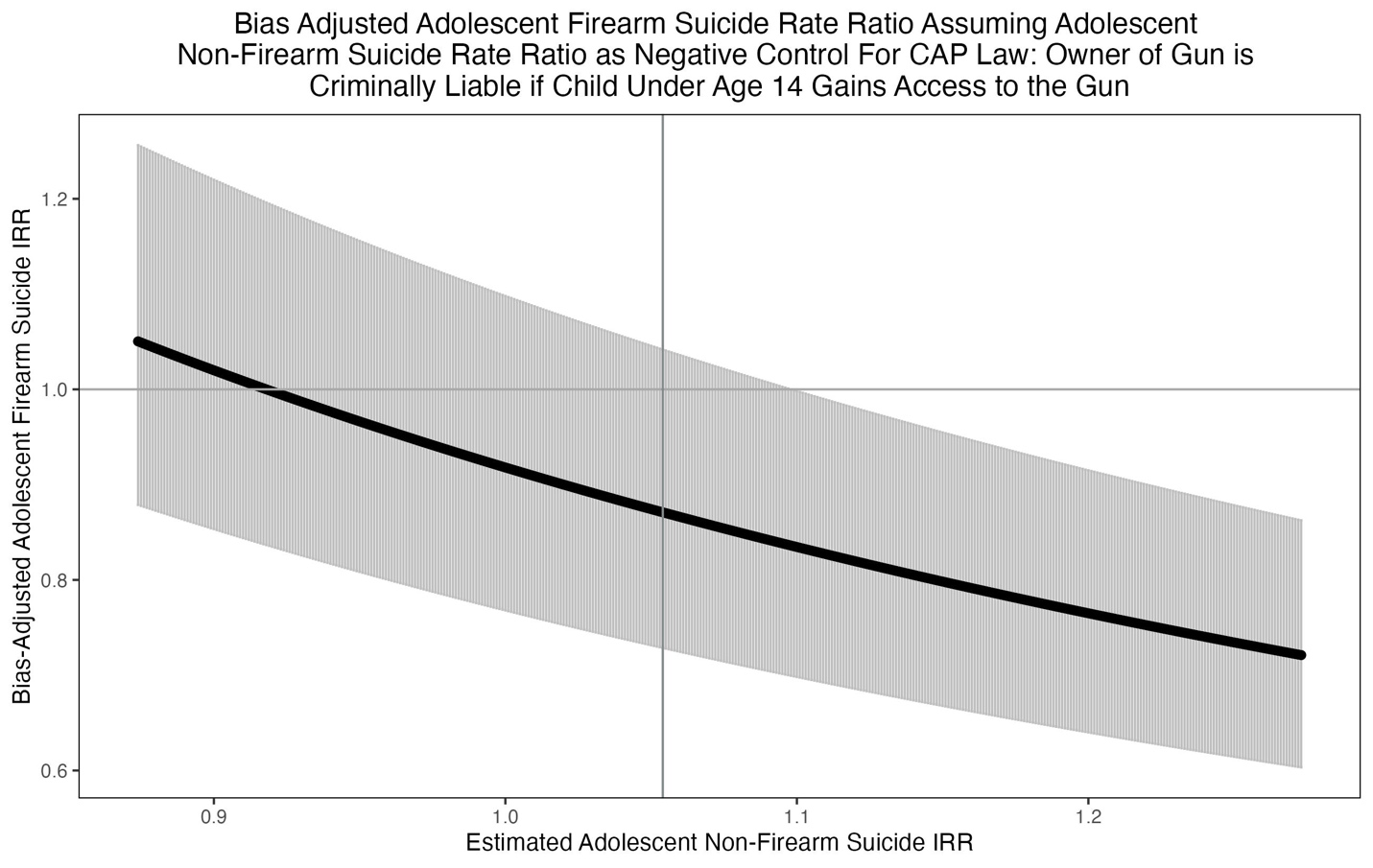
**Figure S10**-Owner of Gun is Criminally Liable if Child Under Age 14 Gains Access to the Gun:

Black dots represent the bias-adjusted point estimates. Error bars represent the bias-adjusted 95% confidence intervals. The vertical line originating at 1.054 on the x-axis represents the Kivisto and colleagues estimated adolescent non-firearm suicide IRR. The horizontal line originating from the y-axis represents the null value. IRR=Incident Rate Ratio.

**
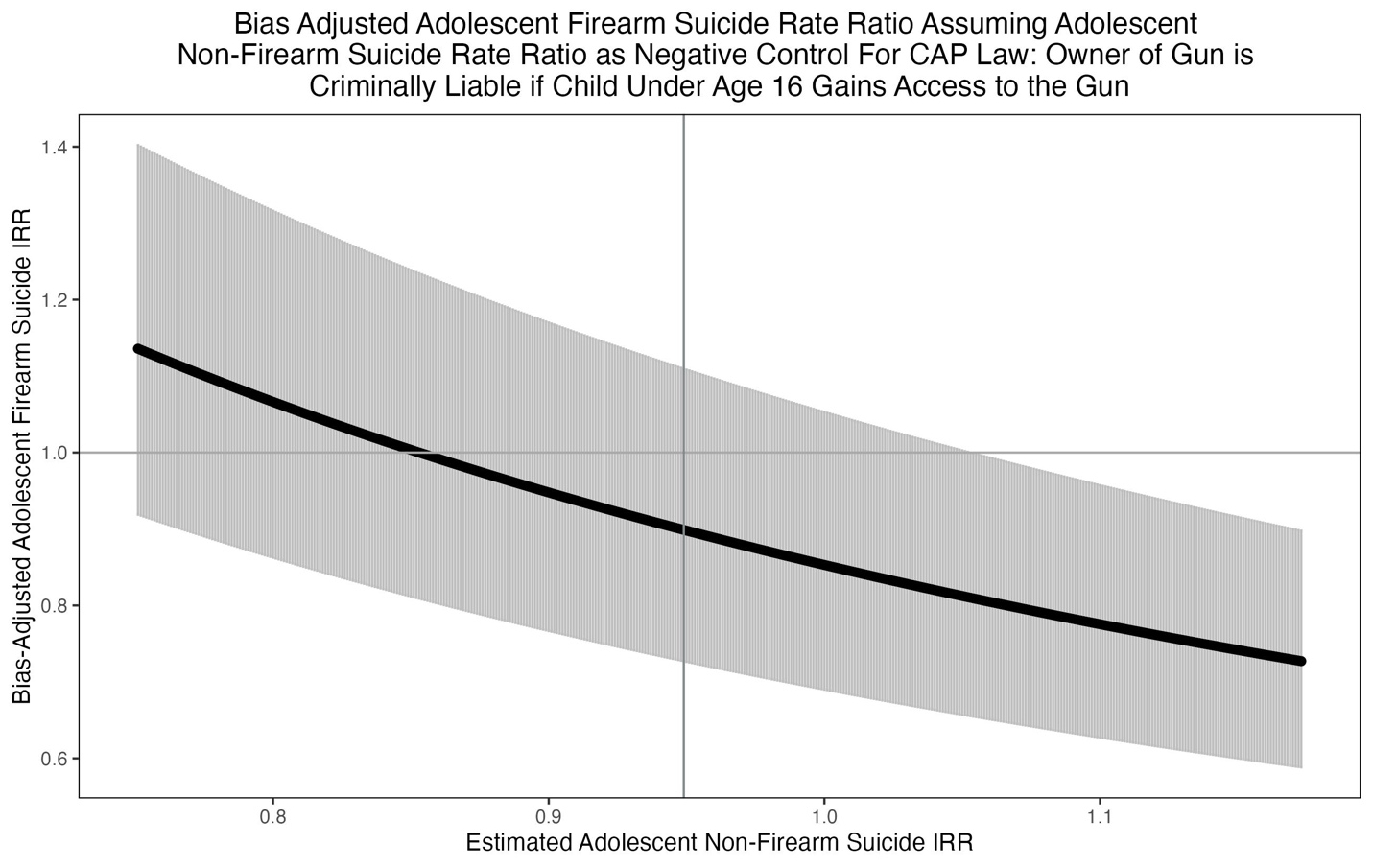
Figure S11**-Owner of Gun is Criminally Liable if Child Under Age 16 Gains Access to the Gun:

Black dots represent the bias-adjusted point estimates. Error bars represent the bias-adjusted 95% confidence intervals. The vertical line originating at 0.949 on the x-axis represents the Kivisto and colleagues estimated adolescent non-firearm suicide IRR. The horizontal line originating from the y-axis represents the null value. IRR=Incident Rate Ratio.

**Figure S12**-Owner of Gun is Criminally Liable if Child Under Age 18 Gains Access to the Gun:
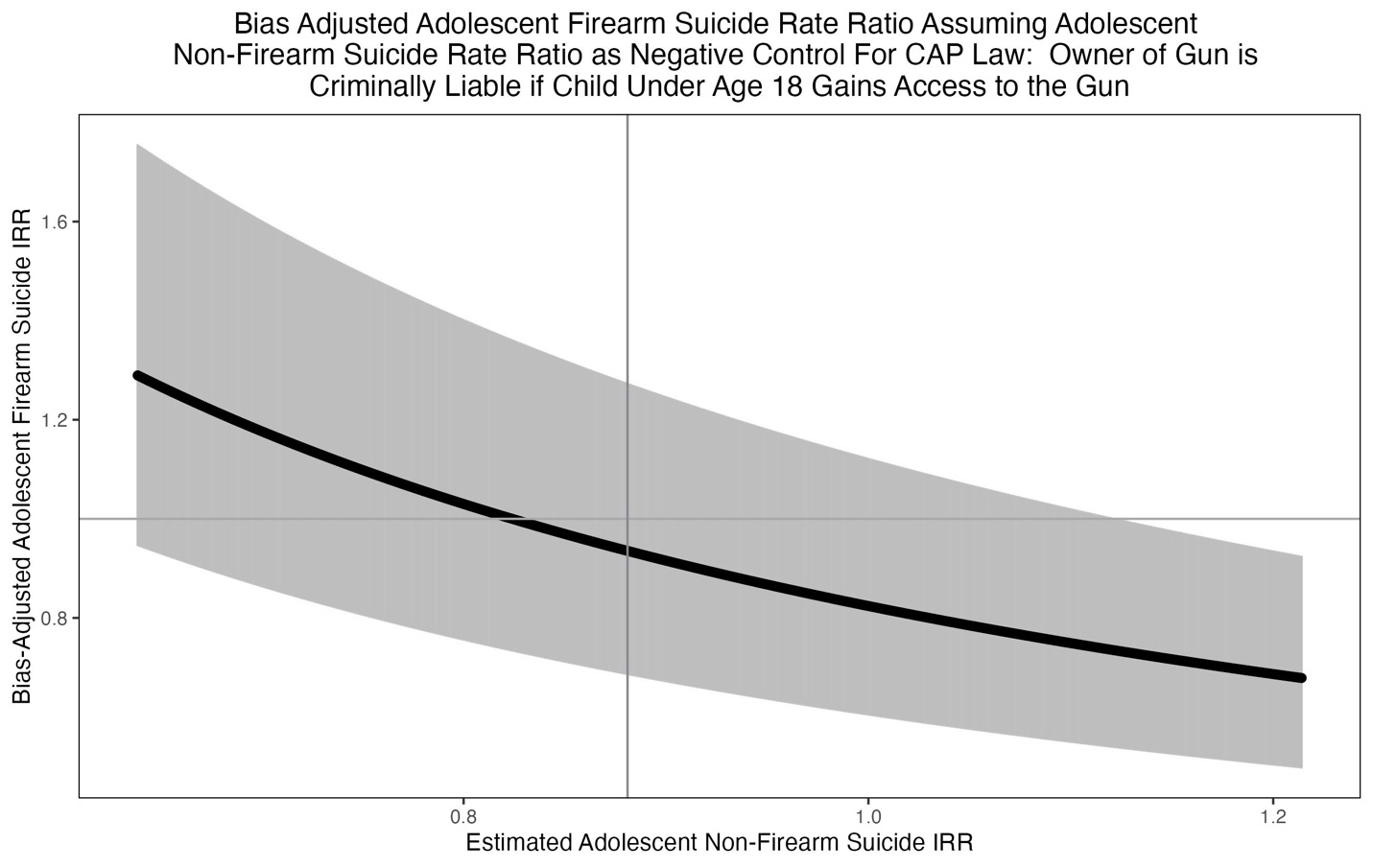
Black dots represent the bias-adjusted point estimates. Error bars represent the bias-adjusted 95% confidence intervals. The vertical line originating at 0.881 on the x-axis represents the Kivisto and colleagues estimated adolescent non-firearm suicide IRR. The horizontal line originating from the y-axis represents the null value. IRR=Incident Rate Ratio.

**Supplement 3-Supplemental bias analyses for specific versions of CAP laws using adult firearm suicide as the negative control:**

**
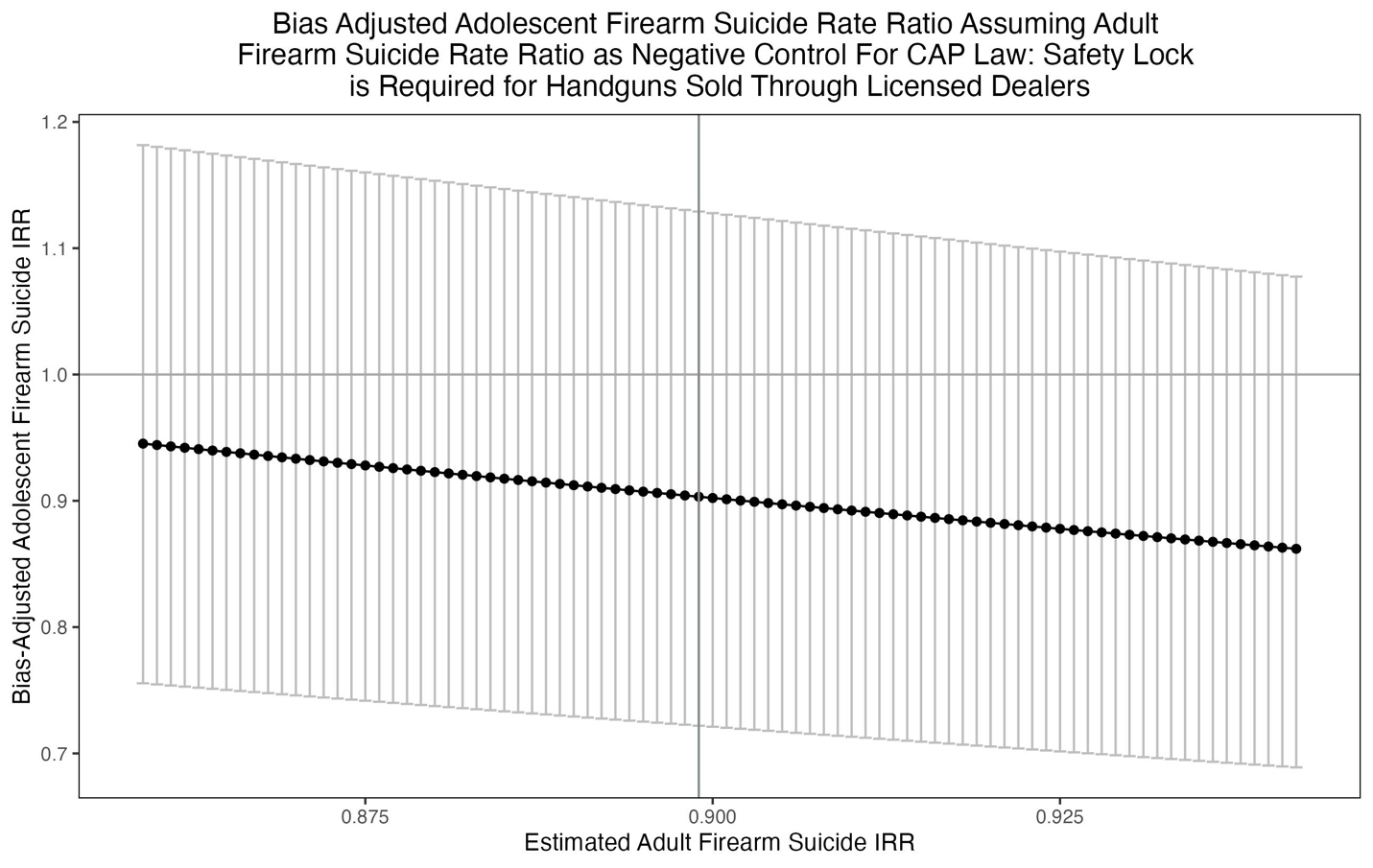
Figure S13**-Safety Lock is Required for Handguns Sold Through Licensed Dealers:

Black dots represent the bias-adjusted point estimates. Error bars represent the bias-adjusted 95% confidence intervals. The vertical line originating at 0.899 on the x-axis represents the Kivisto and colleagues estimated adult firearm suicide IRR. The horizontal line originating from the y-axis represents the null value. IRR=Incident Rate Ratio.


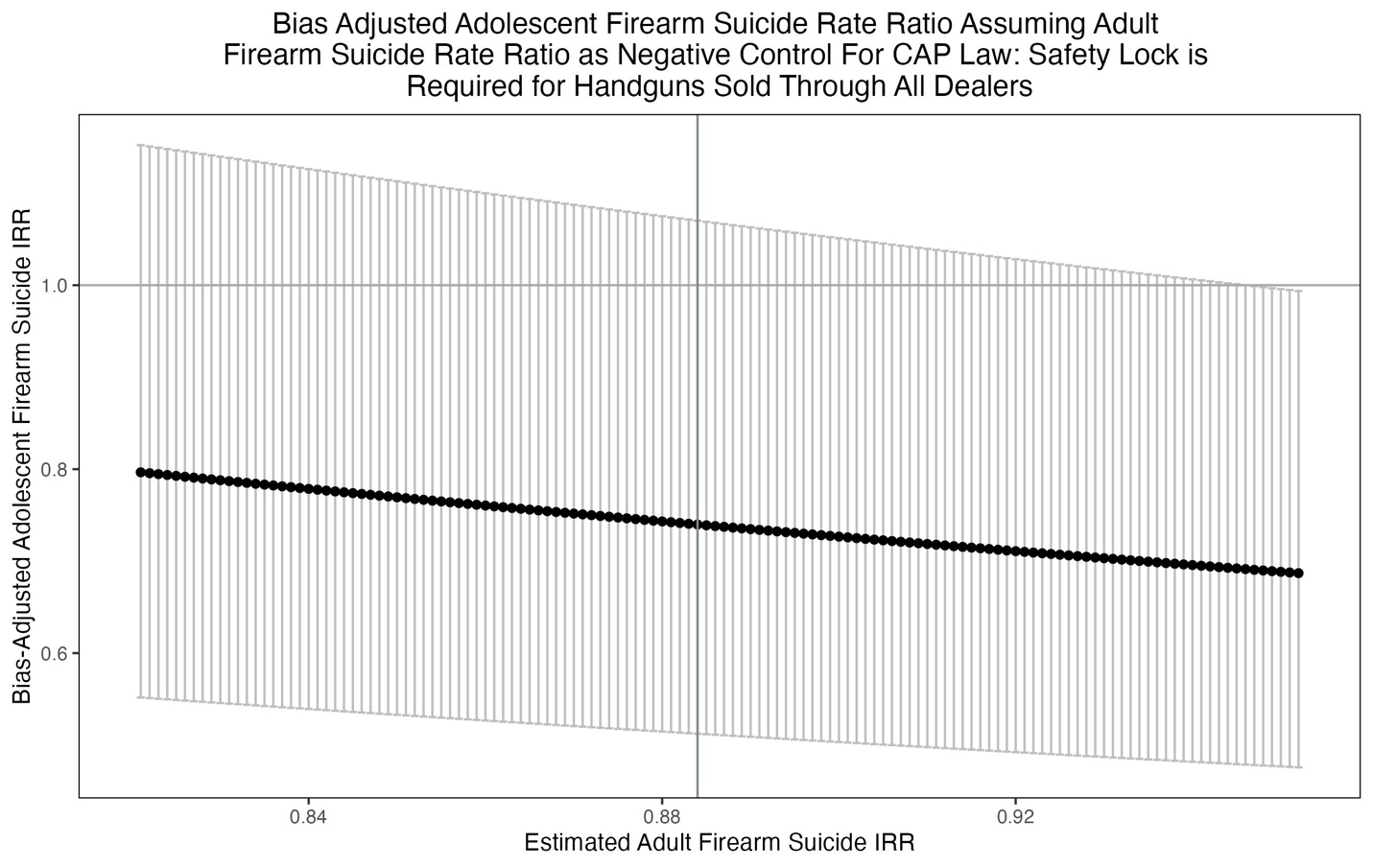
**Figure S14**-Safety Lock is Required for Handguns Sold Through All Dealers:

Black dots represent the bias-adjusted point estimates. Error bars represent the bias-adjusted 95% confidence intervals. The vertical line originating at 0.884 on the x-axis represents the Kivisto and colleagues estimated adult firearm suicide IRR. The horizontal line originating from the y-axis represents the null value. IRR=Incident Rate Ratio.

**
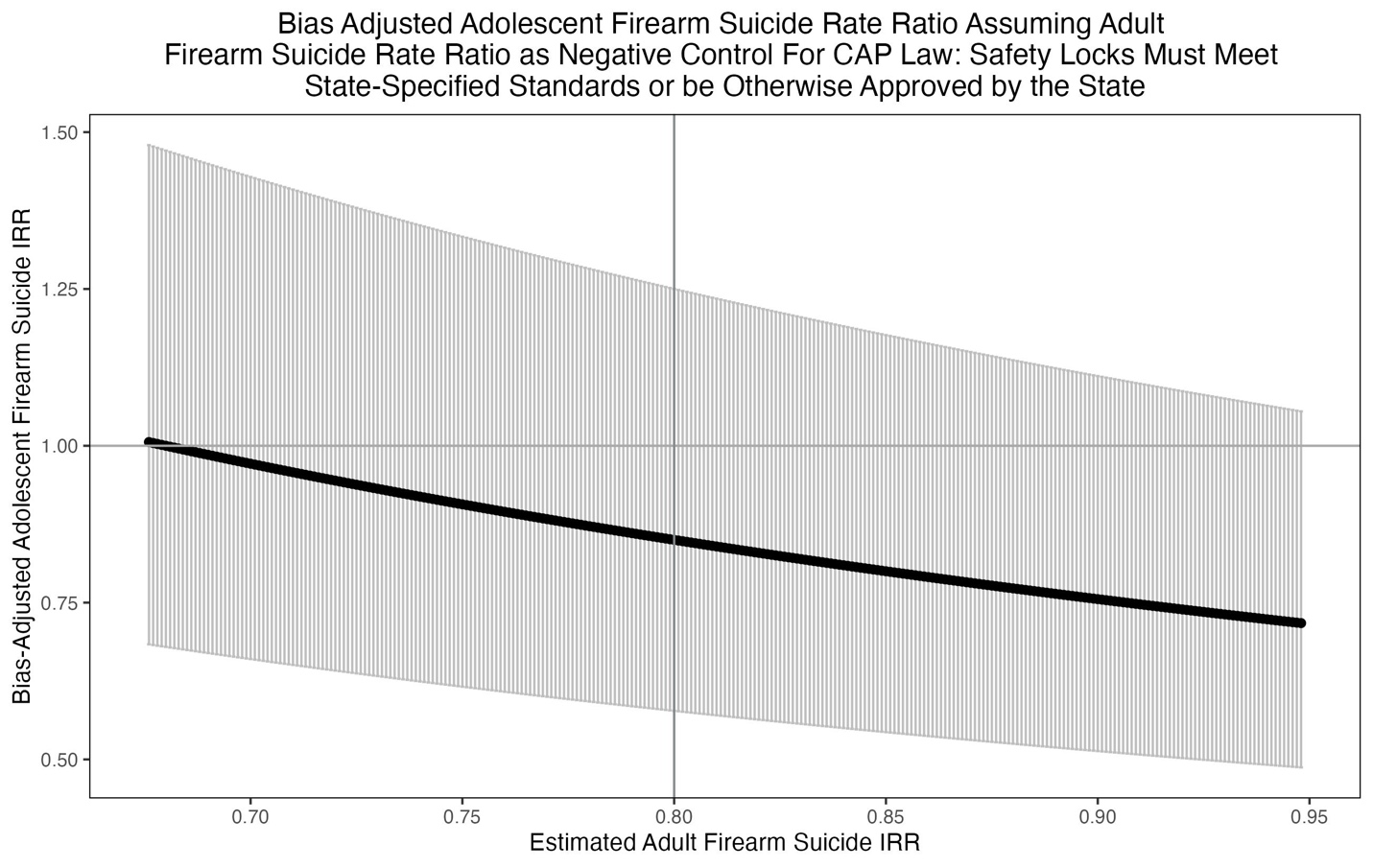
Figure S15**-Safety Locks Must Meet State-Specified Standards or be Otherwise Approved by the State:

Black dots represent the bias-adjusted point estimates. Error bars represent the bias-adjusted 95% confidence intervals. The vertical line originating at 0.800 on the x-axis represents the Kivisto and colleagues estimated adult firearm suicide IRR. The horizontal line originating from the y-axis represents the null value. IRR=Incident Rate Ratio.

**
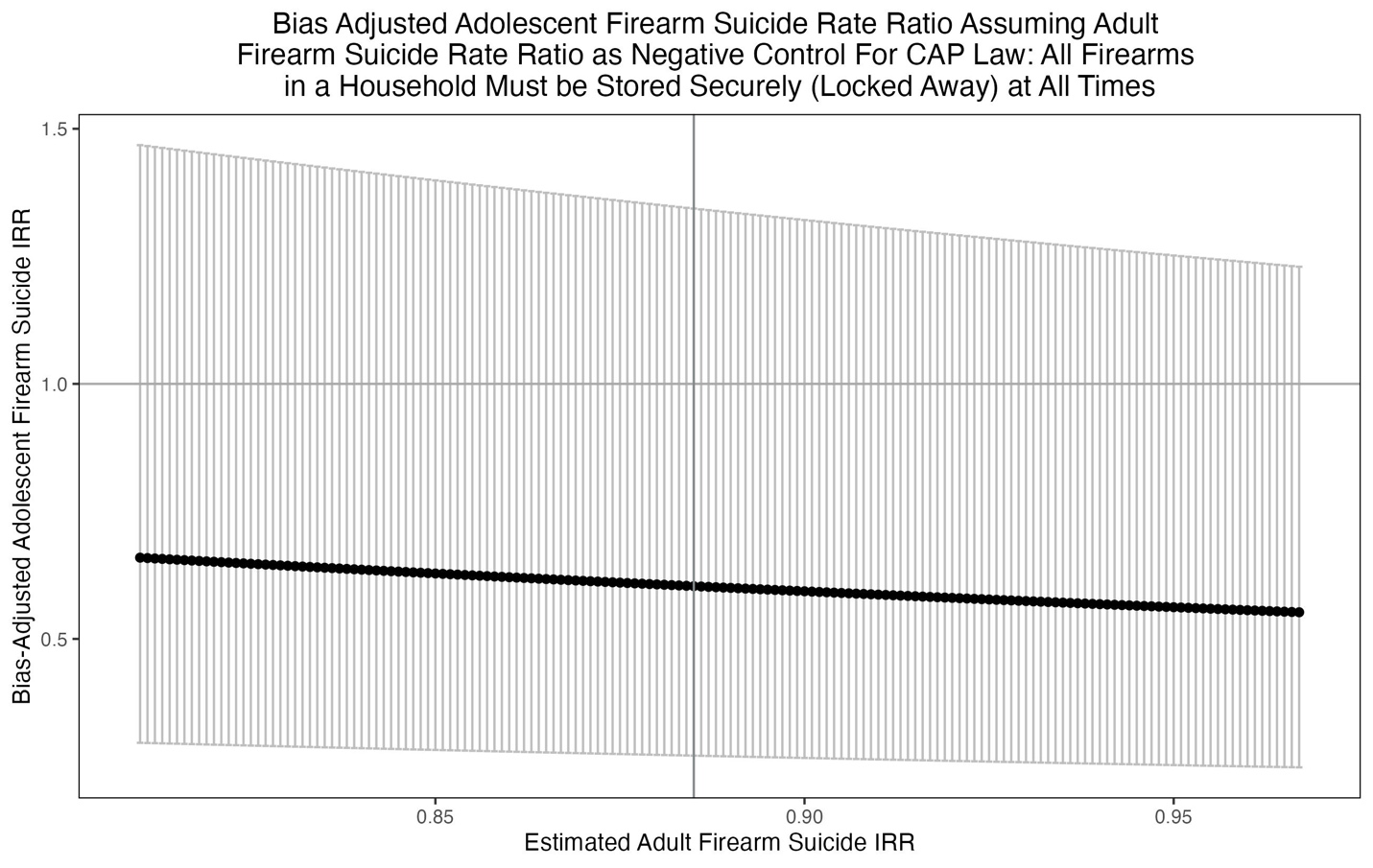
Figure S16**-All Firearms in a Household Must be Stored Securely (Locked Away) at All Times:

Black dots represent the bias-adjusted point estimates. Error bars represent the bias-adjusted 95% confidence intervals. The vertical line originating at 0.885 on the x-axis represents the Kivisto and colleagues estimated adult firearm suicide IRR. The horizontal line originating from the y-axis represents the null value. IRR=Incident Rate Ratio.


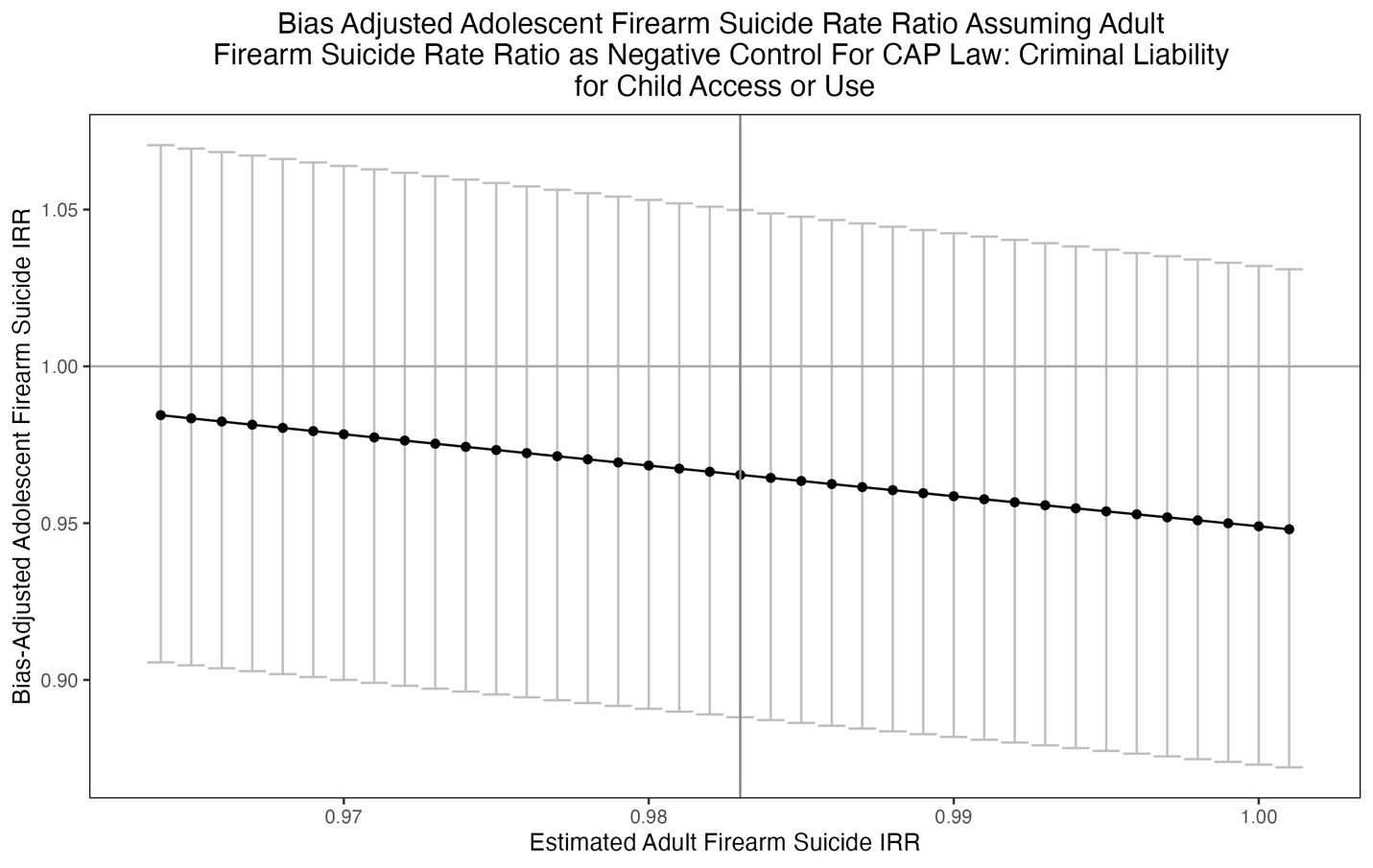
**Figure S17**-Criminal Liability for Child Access or Use:

Black dots represent the bias-adjusted point estimates. Error bars represent the bias-adjusted 95% confidence intervals. The vertical line originating at 0.983 on the x-axis represents the Kivisto and colleagues estimated adult firearm suicide IRR. The horizontal line originating from the y-axis represents the null value. IRR=Incident Rate Ratio.


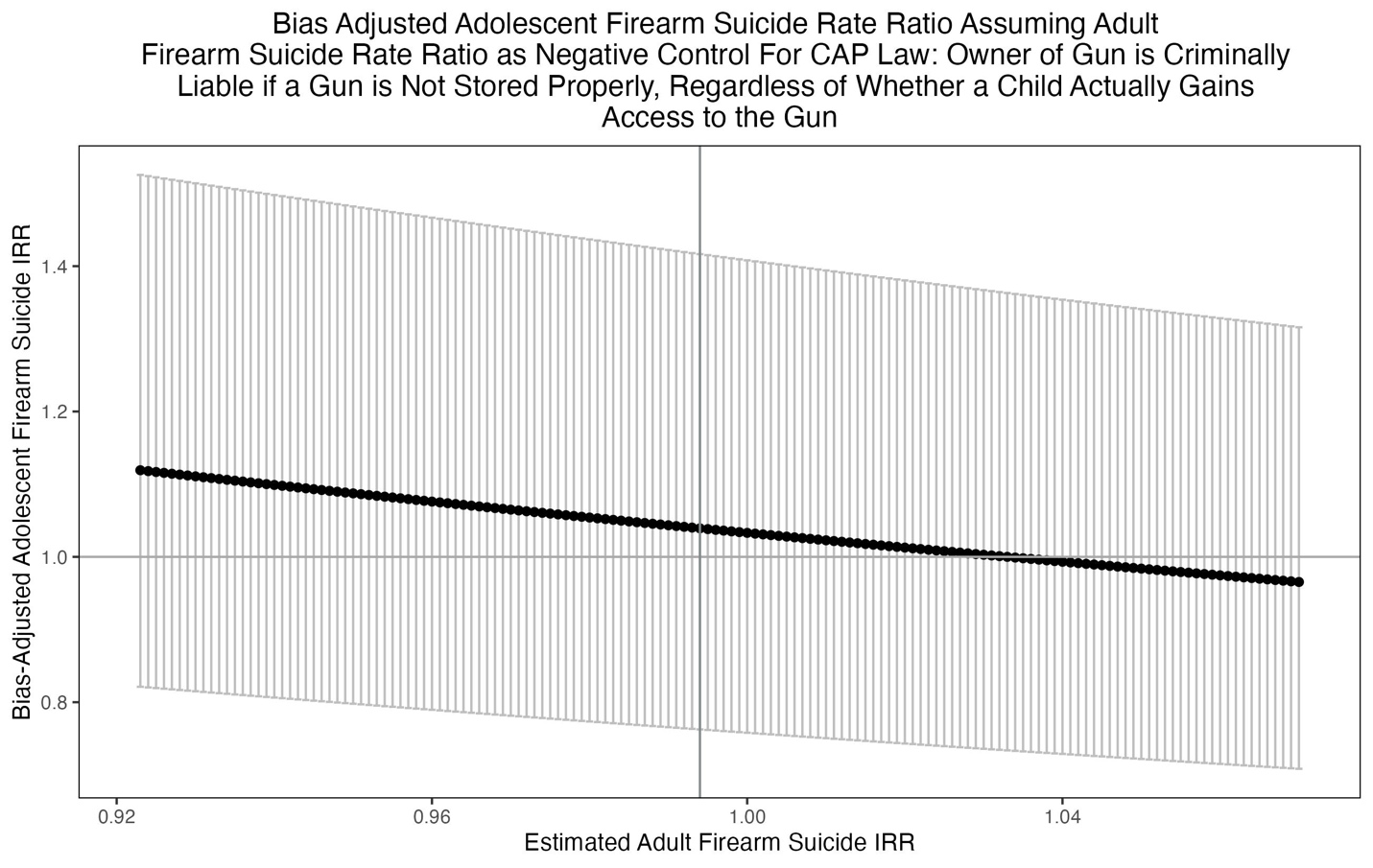
**Figure S18**-Owner of Gun is Criminally Liable if a Gun is Not Stored Properly, Regardless of Whether a Child Actually Gains Access to the Gun:

Black dots represent the bias-adjusted point estimates. Error bars represent the bias-adjusted 95% confidence intervals. The vertical line originating at 0.994 on the x-axis represents the Kivisto and colleagues estimated adult firearm suicide IRR. The horizontal line originating from the y-axis represents the null value. IRR=Incident Rate Rat


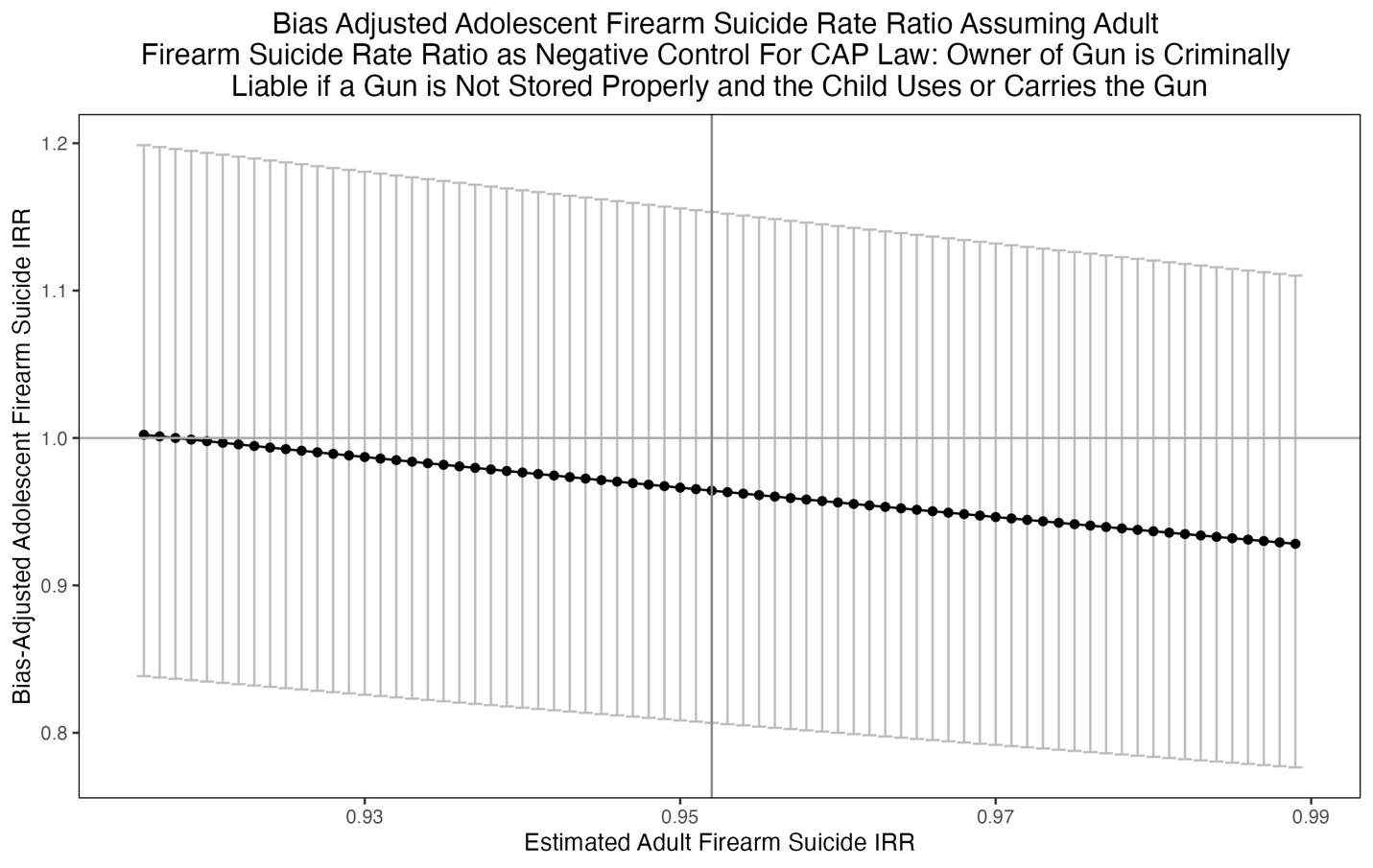
**Figure S19**-Owner of Gun is Criminally Liable if a Gun is Not Stored Properly and the Child Uses or Carries the Gun:

Black dots represent the bias-adjusted point estimates. Error bars represent the bias-adjusted 95% confidence intervals. The vertical line originating at 0.952 on the x-axis represents the Kivisto and colleagues estimated adult firearm suicide IRR. The horizontal line originating from the y-axis represents the null value. IRR=Incident Rate Ratio.


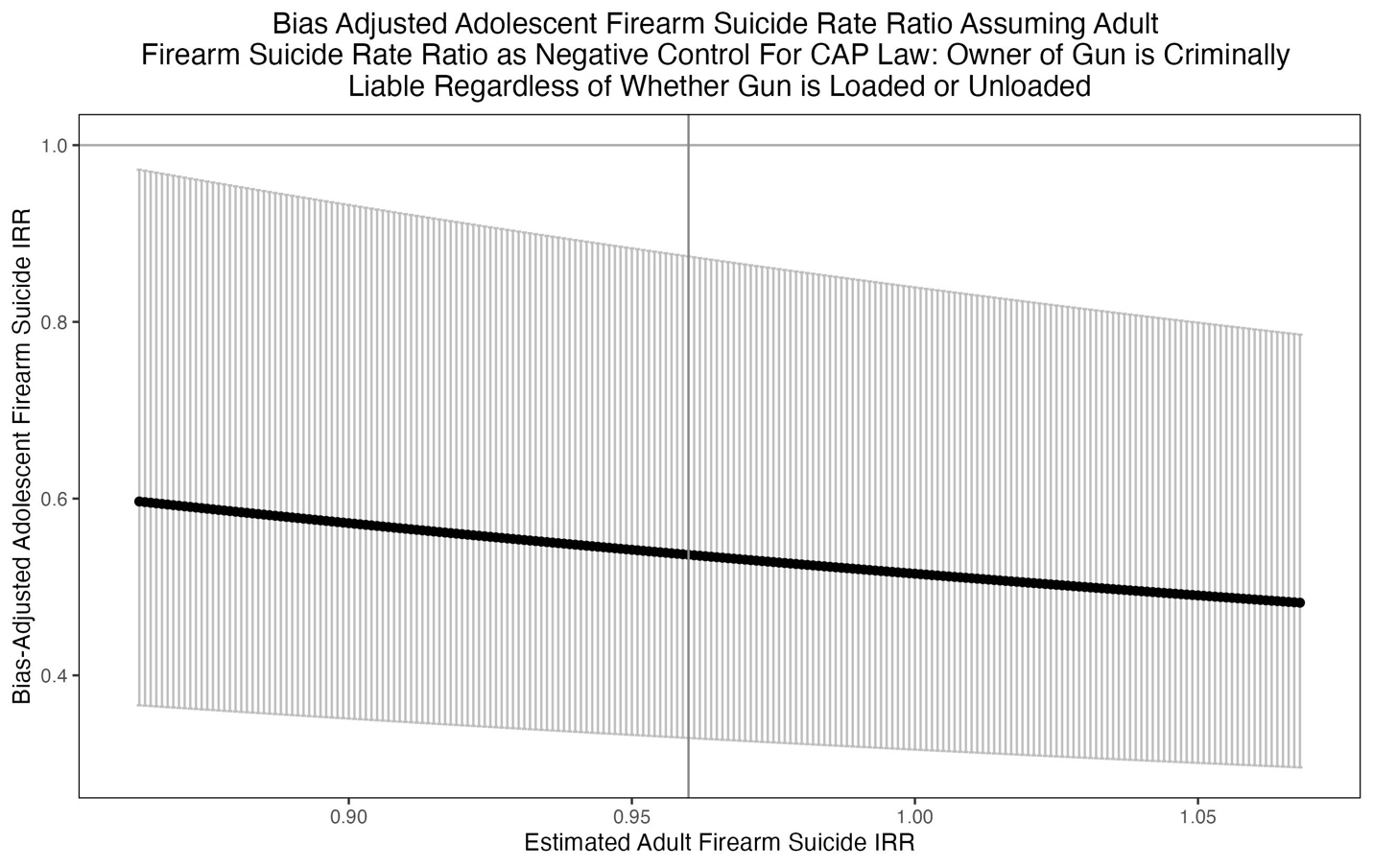
**Figure S20**-Owner of Gun is Criminally Liable Regardless of Whether Gun is Loaded or Unloaded:

Black dots represent the bias-adjusted point estimates. Error bars represent the bias-adjusted 95% confidence intervals. The vertical line originating at 0.960 on the x-axis represents the Kivisto and colleagues estimated adult firearm suicide IRR. The horizontal line originating from the y-axis represents the null value. IRR=Incident Rate Ratio.


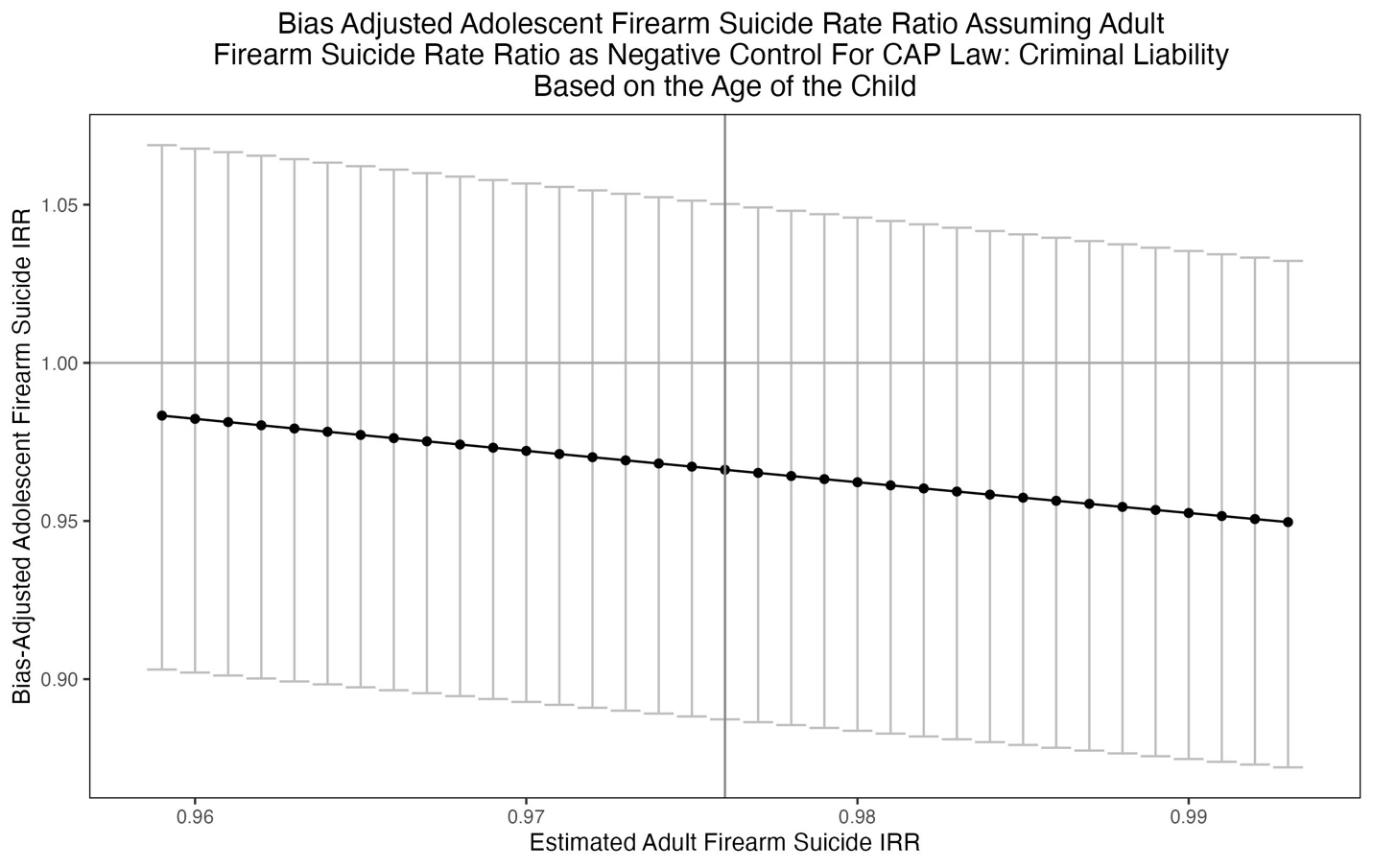
**Figure S21**-Criminal Liability Based on the Age of the Child:

Black dots represent the bias-adjusted point estimates. Error bars represent the bias-adjusted 95% confidence intervals. The vertical line originating at 0.976 on the x-axis represents the Kivisto and colleagues estimated adult firearm suicide IRR. The horizontal line originating from the y-axis represents the null value. IRR=Incident Rate Ratio.


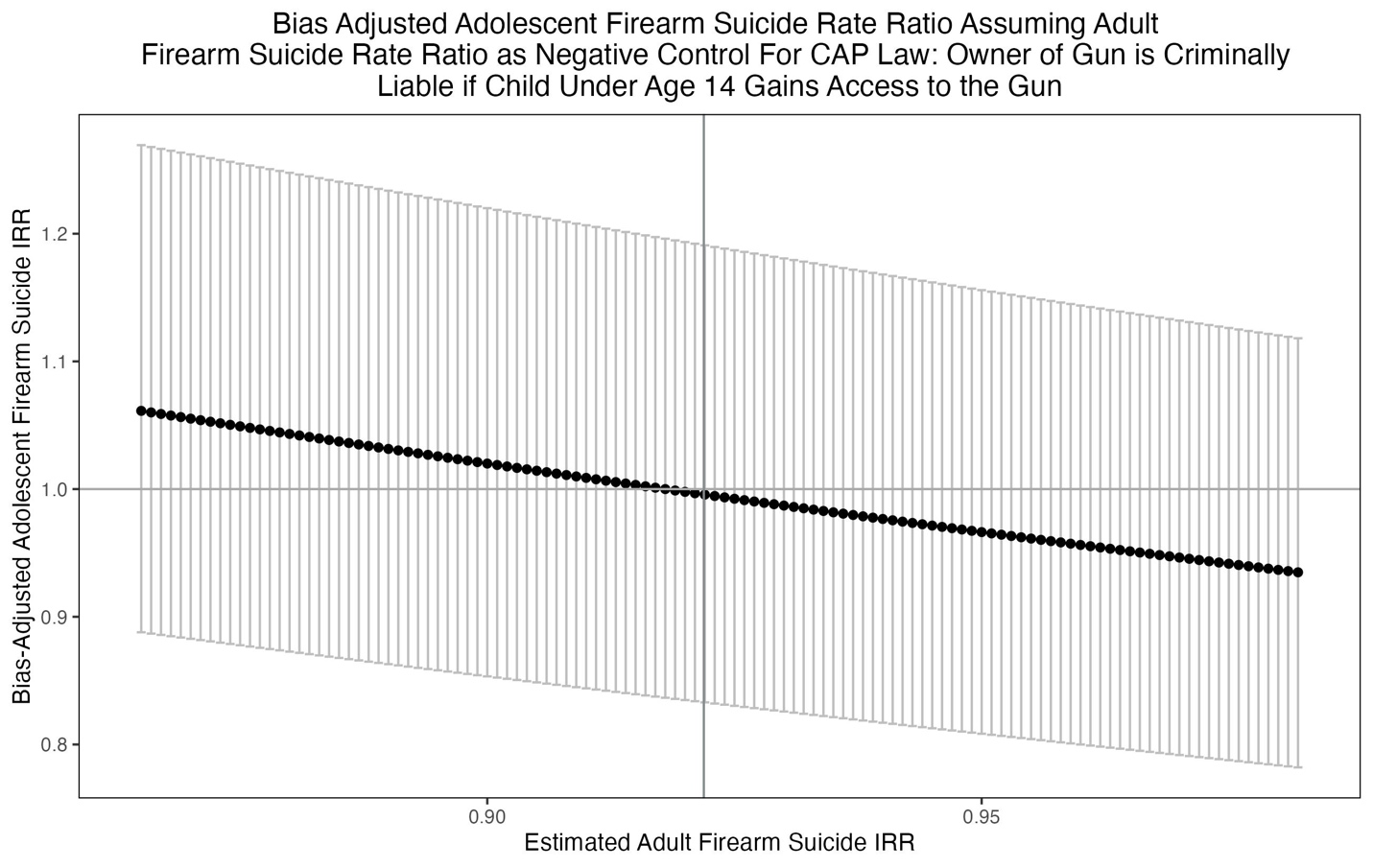
**Figure S22**-Owner of Gun is Criminally Liable if Child Under Age 14 Gains Access to the Gun:

Black dots represent the bias-adjusted point estimates. Error bars represent the bias-adjusted 95% confidence intervals. The vertical line originating at 0.922 on the x-axis represents the Kivisto and colleagues estimated adult firearm suicide IRR. The horizontal line originating from the y-axis represents the null value. IRR=Incident Rate Ratio.


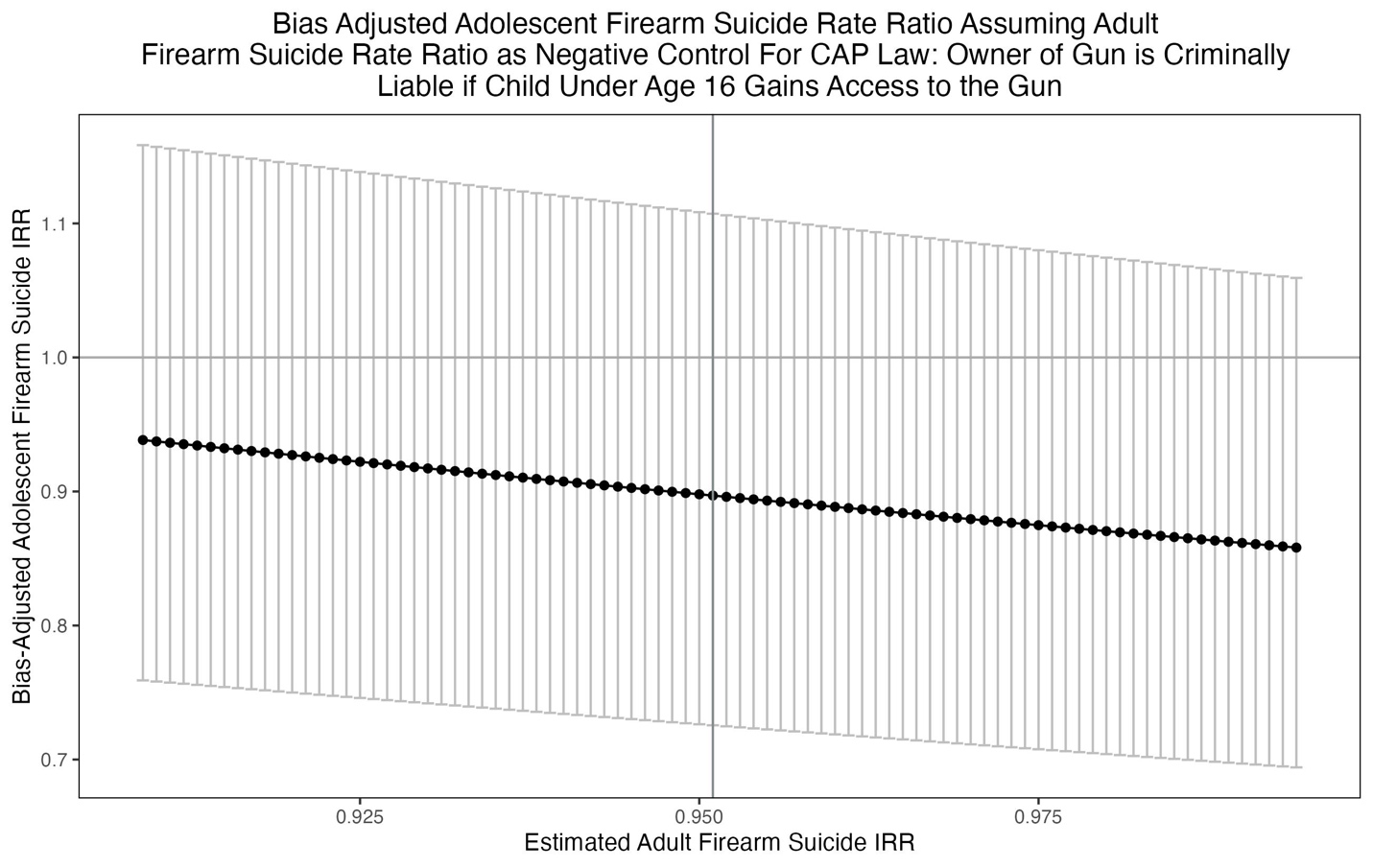
**Figure S23**-Owner of Gun is Criminally Liable if Child Under Age 16 Gains Access to the Gun:

Black dots represent the bias-adjusted point estimates. Error bars represent the bias-adjusted 95% confidence intervals. The vertical line originating at 0.951 on the x-axis represents the Kivisto and colleagues estimated adult firearm suicide IRR. The horizontal line originating from the y-axis represents the null value. IRR=Incident Rate Ratio.


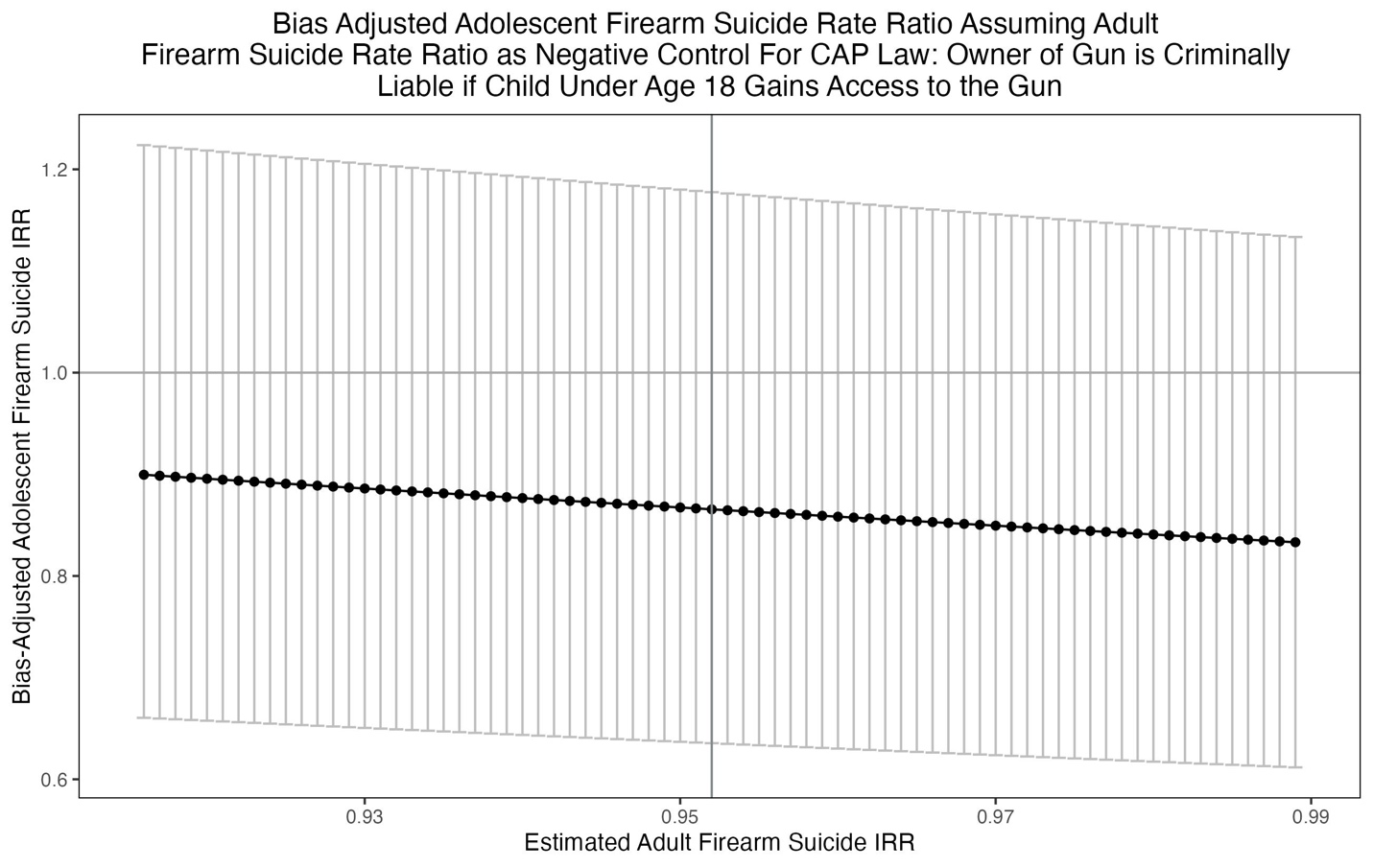
**Figure S24**-Owner of Gun is Criminally Liable if Child Under Age 18 Gains Access to the Gun:

Black dots represent the bias-adjusted point estimates. Error bars represent the bias-adjusted 95% confidence intervals. The vertical line originating at 0.952 on the x-axis represents the Kivisto and colleagues estimated adult firearm suicide IRR. The horizontal line originating from the y-axis represents the null value. IRR=Incident Rate Ratio.
